# Chiral hydroxymethyl-1*H*,3*H*-pyrrolo[1,2-*c*]thiazoles: the search for selective p53-activating agents for colorectal cancer therapy†

**DOI:** 10.1039/d4md00076e

**Published:** 2024-04-12

**Authors:** Mees M. Hendrikx, Adelino M. R. Pereira, Ana B. Pereira, Carla S. C. Carvalho, João L. P. Ribeiro, Maria I. L. Soares, Lucília Saraiva, Teresa M. V. D. Pinho e Melo

**Affiliations:** a University of Coimbra, Coimbra Chemistry Centre – Institute of Molecular Sciences and Department of Chemistry 3004-535 Coimbra Portugal tmelo@ci.uc.pt; b LAQV/REQUIMTE, Laboratório de Microbiologia, Departamento de Ciências Biológicas, Faculdade de Farmácia, Universidade do Porto Porto Portugal

## Abstract

MANIO is an efficient p53-activating anticancer agent with remarkable selectivity to the p53 pathway and promising antitumor activity against colorectal cancer (CRC). Herein, a library of novel MANIO derivatives, including hydroxymethyl- and bis(hydroxymethyl)-1*H*,3*H*-pyrrolo[1,2-*c*]thiazoles, was synthesized by rational structural modulation. The antiproliferative activity of twenty derivatives was evaluated in a panel of human CRC cells with different p53 status. From this library, five compounds with *R*- and *S*-configuration and with aromatic or heteroaromatic groups at position 3, including the enantiomer of MANIO, were identified as selective towards p53-expressing cancer cells. On the other hand, two compounds with *S*-configuration, 6-hydroxymethyl- and 7-hydroxymethyl-5-methyl-3-phenyl-1*H*,3*H*-pyrrolo[1,2-*c*]thiazoles, showed high cytotoxicity against WTp53-expressing HCT116 colon cells but, unlike MANIO, exhibited p53-independent inhibitory activity in CRC. The results described provide relevant structural and pharmacophoric data for the design of new p53-activating agents for precision therapy of CRC or other p53-related cancers harboring both wild-type or mutated p53 forms.

## Abstract 2

Structural modulation of a lead p53-activating anticancer molecule was performed, leading to the synthesis of new bioactive molecules. Activity against human colorectal cancer cells with different p53 status provided in-depth SAR data.

## Graphical abstract

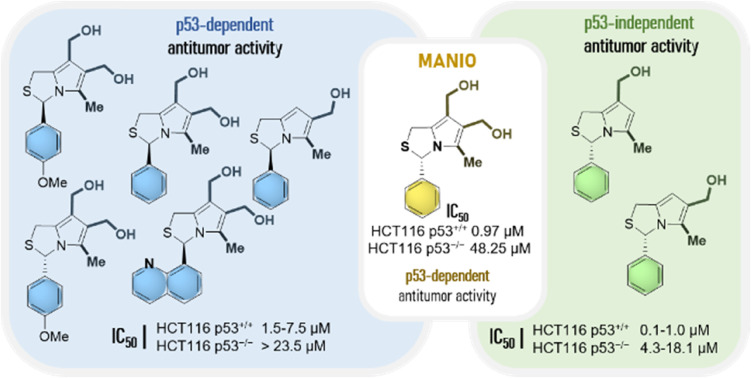

## Introduction

Cancer is currently a major public health concern, with both incidence and mortality rates expected to increase in the coming decades.^1,2^ Among the various types of cancer, colorectal cancer (CRC) is the third most common cancer worldwide and the second leading cause of cancer-related deaths.^3,4^ The high mortality rates, often associated with treatment resistance and metastasization, highlight the limited effectiveness of existing therapies in treating CRC.^4^ Progress in understanding the development of CRC has revealed that p53 dysfunction is a key event in both localized and advanced cases of CRC. Impairment of the p53 pathway, either by TP53 mutation or inhibition of p53 by its negative regulators, is a significant event in both localized and advanced CRC. Thus, restoration of p53 activity has emerged as one of the most attractive anticancer therapeutic strategies.

**Fig. 1:**
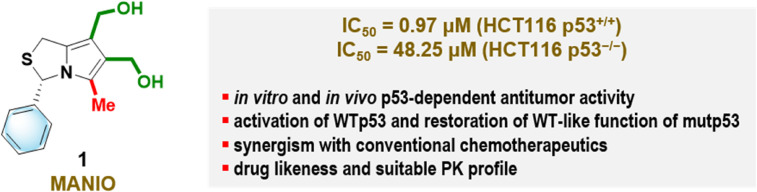
Chemical structure and key features of (3*S*)-6,7-bis(hydroxymethyl)-5-methyl-3-phenyl-1*H*,3*H*-pyrrolo[1,2-*c*]thiazole (MANIO) (1).

Recently, our research team disclosed a new p53-activating anticancer drug, (3*S*)-6,7-bis(hydroxymethyl)-5-methyl-3-phenyl-1*H*,3*H*-pyrrolo[1,2-*c*]thiazole (MANIO) (Fig. 1).^5^ MANIO represents a privileged anticancer drug compared to other currently available p53-activating agents regarding its antitumor activity in several cancer types, including CRC. MANIO showed a remarkable selectivity to the p53 pathway, activating wild-type (WT)p53 and restoring WT-like function to mutant (mut)p53 in human cancer cells. The half maximal inhibitory concentration (IC_50_) value of MANIO in WTp53-expressing HCT116 colon cells, HCT116 p53^+/+^ (0.97 μM), was approximately 50-fold lower than that obtained in HCT116 p53^−/−^cells (48.25 μM), indicating a significant p53-dependent growth inhibitory effect. MANIO has been shown to directly interact with the p53 DNA-binding domain (DBD), leading to activation of the p53 pathway. In fact, MANIO works as a bridging molecule between p53 and DNA. Ramos *et al.* have demonstrated that MANIO binds to a pocket formed between one dimer of the WTp53 DBD protein and the minor groove of the DNA molecule.^5^ MANIO interacts with the WTp53 DBD protein backbone through hydrogen bonds between its hydroxyl groups and the amide backbone groups of methionine 243 of chain A (M243A) and methionine 243 of chain B (M243B) (each belong to a different monomer). The remaining interactions of MANIO are stacking interactions made with the bases of DNA (DG8, DG12). In the specific case of mutp53 R248W, a highly frequent p53 mutation, in most clusters, the ligand makes a hydrogen bond between one of its hydroxyl oxygen atoms and the W248 side-chain nitrogen. The ligand phenyl group is close to the DNA bases adenine 6 and thymine 7. These interactions may eventually compensate for the loss of direct contacts between lysine 248 residue and the DNA. The latter interaction results in increased p53 stability, enhanced DNA-binding capacity and increased transcriptional activity. MANIO synergizes with conventional chemotherapeutics (*e.g.*, doxorubicin (DOXO), cisplatin (CISP) and 5-fluorouracil (5-FU)) in patient-derived and immortalized CRC cells expressing WTp53 or mutp53. In addition, MANIO demonstrated *in vivo* p53-dependent anti-tumor activity in xenograft mouse models of CRC with no adverse side effects. It also demonstrated favorable drug-likeness and pharmacokinetic (PK) properties for a clinical candidate.^5^

In this study, structural modulation of MANIO, a lead p53-activating anticancer molecule, was performed with two main goals: to synthesize new bioactive 1*H*,3*H*-pyrrolo[1,2-*c*]thiazoles and to gain in-depth knowledge of structure–activity relationships (SAR).

## Results and discussion

### Synthesis of new MANIO-like derivatives

The structural modulation strategy focused on structural changes of MANIO at position 3 of the 1*H*,3*H*-pyrrolo[1,2-*c*]thiazole core, namely the introduction of new aromatic, heteroaromatic, and alkyl substituents and the modification of the absolute configuration. These changes included the introduction of *p*-fluorophenyl, *p*-(trifluoromethoxy)phenyl, *p*-methoxyphenyl, bulky naphthyl and quinolinyl groups, and alkyl groups such as benzyl and methyl. Modifications in other positions of the bicyclic system involved the removal of one hydroxymethyl group or the oxidation of the sulphur atom.

**Scheme 1:**
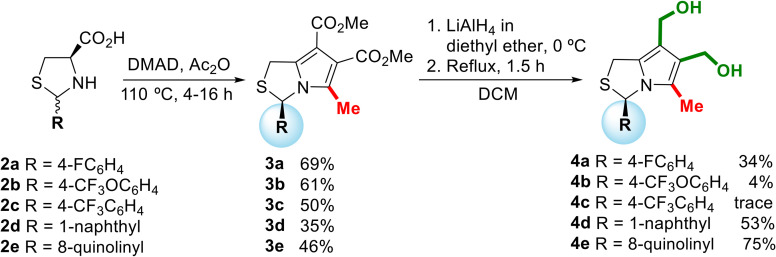
Synthesis of chiral 6,7-bis(hydroxymethyl)-1*H*,3*H*-pyrrolo[1,2-*c*]thiazoles 4.

Chiral 1*H*,3*H*-pyrrolo[1,2-*c*]thiazoles with *R*-configuration 3 were synthesized in moderate yields (35–69%) according to a known synthetic procedure (Scheme 1).^6^ Thiazolidines 2 were obtained from the reaction of l-cysteine with the corresponding aromatic aldehydes as mixtures of 2*R*,4*R*- and 2*S*,4*R*-diastereoisomers. The synthetic sequence proceeds *via* 1,3-dipolar cycloaddition of dimethyl acetylenedicarboxylate (DMAD) with the bicyclic münchnone generated *in situ* from thiazolidine 2, followed by elimination of carbon dioxide. The acylation of the diastereoisomeric mixtures of thiazolidines with acetic anhydride or acyl chlorides can lead the to the selective synthesis of *N*-acetyl-thiazolidine-4-carboxylic acids as pure stereoisomers with 2*S*,4*R*- or 2*R*,4*R*-configuration, depending on the reaction conditions.^7–9^ Thiazolidine 2 was heated in a solution of acetic anhydride in the presence of the dipolarophile. Under these reaction conditions the *N*-acylation occurs *in situ* giving selectively *N*-acetyl thiazolidine-4-carboxylic acids with 2*R*,4*R*-configuration. Thus, starting from thiazolidine-4-carboxylic acids 2 as a mixture of 2*R*,4*R*- and 2*S*,4*R*-diastereoisomers, chiral 1*H*-pyrrolo[1,2-*c*]thiazoles 3 were obtained as single enantiomer with *R*-configuration. In this process the chirality at C-4 of the thiazolidine is lost and the chirality at C-2 (C-3 in the product) is retained. The reduction of 3 was carried out with lithium aluminium hydride to afford the target 6,7-bis(hydroxymethyl)-1*H*,3*H*-pyrrolo[1,2-*c*]thiazoles 4 in moderate to good yields (34–75% for alcohols 4a, 4d and 4e). The formation of alcohols 4b and 4c bearing an OCF_3_ or CF_3_ group in the *para* position of the phenyl group was confirmed by proton NMR spectroscopy. However, we decided not to proceed with the biological evaluation studies due to the lack of stability of these compounds.

**Scheme 2:**
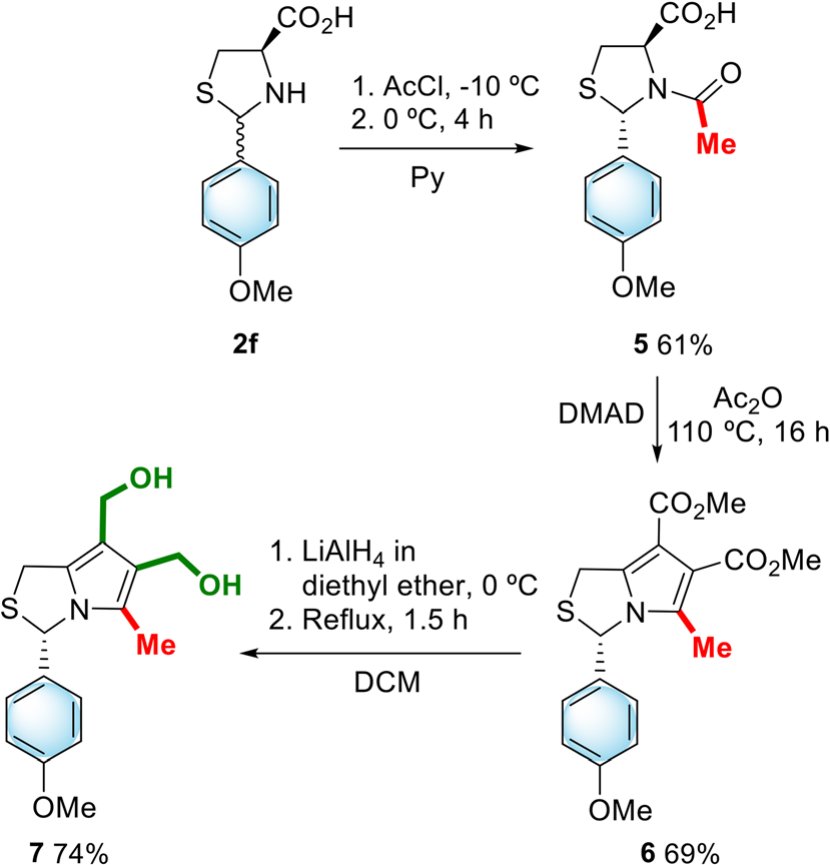
Synthesis of (3*S*)-6,7-bis(hydroxymethyl)-1*H*,3*H*-pyrrolo[1,2-*c*]thiazole 7.

**Scheme 3:**
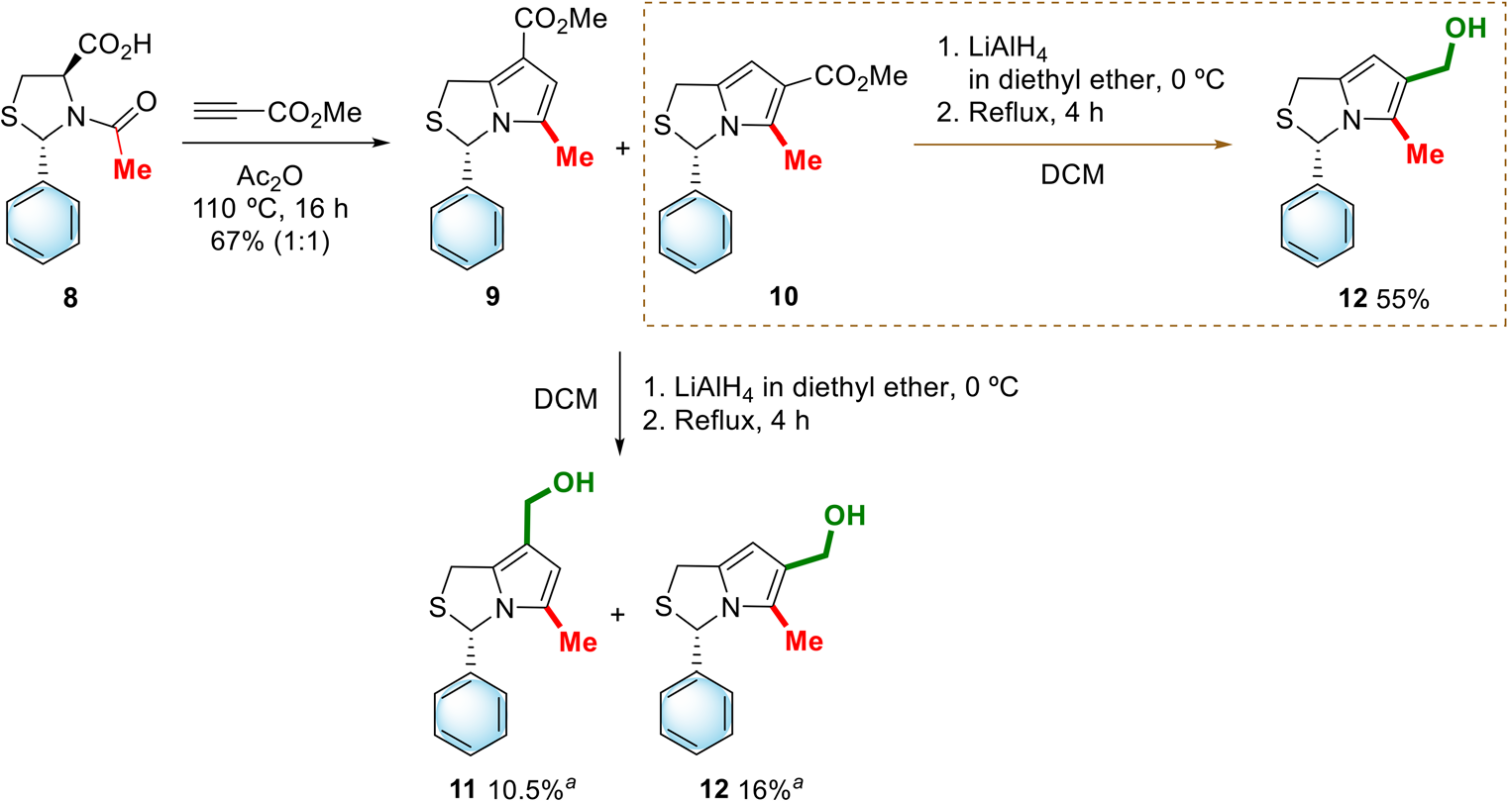
Synthesis of hydroxymethyl-1*H*,3*H*-pyrrolo[1,2-*c*]thiazoles 11 and 12. ^*a*^yield over two steps.

Chiral 6,7-bis(hydroxymethyl)-1*H*,3*H*-pyrrolo[1,2-*c*]thiazole 7 with *S*-configuration was also synthesized (Scheme 2). In order to obtain *N*-acetyl-thiazolidine-4-carboxylic acid with (2*S*,4*R*) stereochemistry we used an experimental procedure previously described in the literature.^10^ The reaction of thiazolidine 2f with acetyl chloride in dry pyridine carried out at 0 °C allowed the selective formation of (2*S*,4*R*)-*N*-acetyl-thiazolidine-4-carboxylic acid 5. Reaction of 5 with DMAD afforded the 1*H*,3*H*-pyrrolo[1,2-*c*]thiazole-6,7-dicarboxylate 6 with *S*-configuration in 69% yield. Further reduction with lithium aluminium hydride afforded 6,7-bis(hydroxymethyl)-1*H*,3*H*-pyrrolo[1,2-*c*]thiazole 7 in good yield (74%).

(3*S*)-7-Hydroxymethyl-5-methyl-3-phenyl-1*H*,3*H*-pyrrolo[1,2-*c*]thiazole (11) and (3*S*)-6-hydroxymethyl-5-methyl-3-phenyl-1*H*,3*H*-pyrrolo[1,2-*c*]thiazole (12), which differ in the position of the hydroxymethyl group, were also synthesized (Scheme 3). Chiral 1*H*,3*H*-pyrrolo[1,2-*c*]thiazole carboxylates 9 and 10 were synthesized in moderate yield (67%) as a regioisomeric mixture (1 : 1) following a known synthetic procedure.^6^ Diastereoisomerically pure (2*S*,4*R*)-*N*-acetyl-thiazolidine-4-carboxylic acid 8^10^ was used to generate the corresponding bicyclic münchnone *in situ*, which reacted with methyl propiolate *via* 1,3-dipolar cycloaddition. The mixture of products could not be separated by chromatography. However, 1*H*,3*H*-pyrrolo[1,2-*c*]thiazole-6-carboxylate 10 was isolated in pure form by selective crystallization from diethyl ether/hexane. The structural assignment of compound 10, made by comparison with the characterization data of the corresponding *R*-enantiomer,^11^ which differed only in the optical rotation, allowed us to conclude that we were in the presence of a 1*H*,3*H*-pyrrolo[1,2-*c*]thiazole-6-carboxylate with *S*-configuration.

The reduction of (3*S*)-1*H*,3*H*-pyrrolo[1,2-*c*]thiazole-6-carboxylate 10 was carried out with lithium aluminium hydride to afford the corresponding alcohol 12 in 55% yield. A mixture of the regioisomeric alcohols 7-hydroxymethyl-1*H*,3*H*-pyrrolo[1,2-*c*]thiazole 11 and 6-hydroxymethyl-1*H*,3*H*-pyrrolo[1,2-*c*]thiazole 12 was obtained by reduction of the crude mixture of the corresponding carboxylate derivatives 9 and 10 with lithium aluminium hydride. Compounds 11 and 12 were successfully separated by column chromatography. This two-step synthetic approach afforded alcohols 11 and 12 in 10.5% and 16% overall yields, respectively.

**Scheme 4:**
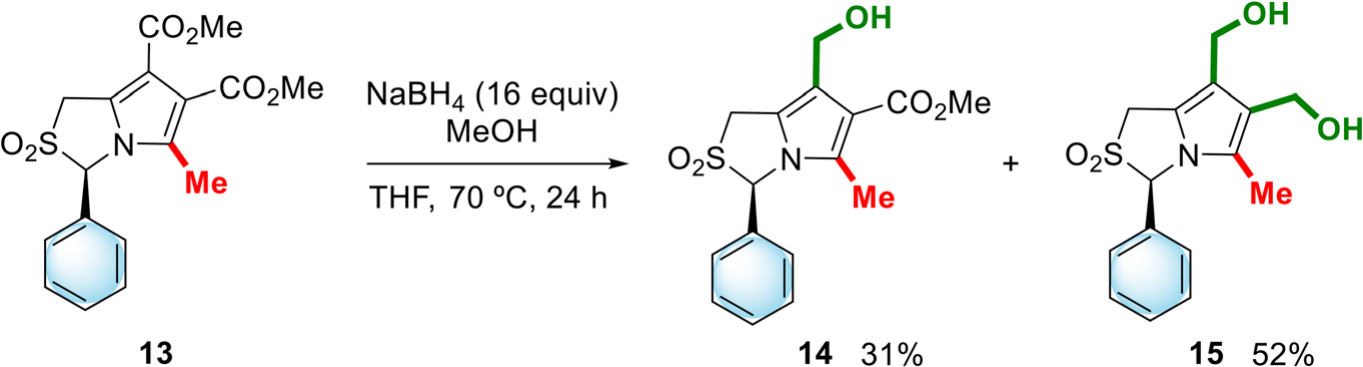
Reduction of dimethyl (3*R*)-5-methyl-2,2-dioxo-3-phenyl-1*H*,3*H*-pyrrolo[1,2-*c*]thiazole-6,7-dicarboxylate (13).

The sulfone functional group is known to impart polarity to molecules, reducing their lipophilicity and improving their solubility in aqueous media, and the cyclic sulfone nucleus in particular is commonly found in biologically active compounds.^12^ Thus, the sulfone of the enantiomer of MANIO was prepared starting from dimethyl (3*R*)-5-methyl-3-phenyl-1*H*,3*H*-pyrrolo[1,2-*c*]thiazole-6,7-dicarboxylate (13), whose synthesis was previously described by our group.^13^ In this case, the reduction was carried out with a sodium borohydride-methanol system instead of LiAlH_4_, as the latter was found to reduce both the ester and the sulfone groups. The reaction of sulfone 13 with the NaBH_4_-methanol system in refluxing THF for 24 h allowed the synthesis of the target sulfone 15 in 52% yield, which was obtained together with the mono-reduced derivative, compound 14, in 31% yield (Scheme 4). Attempts to favor the exclusive formation of compound 15, by prolonging the reaction time, were unsuccessful.

**Scheme 5:**
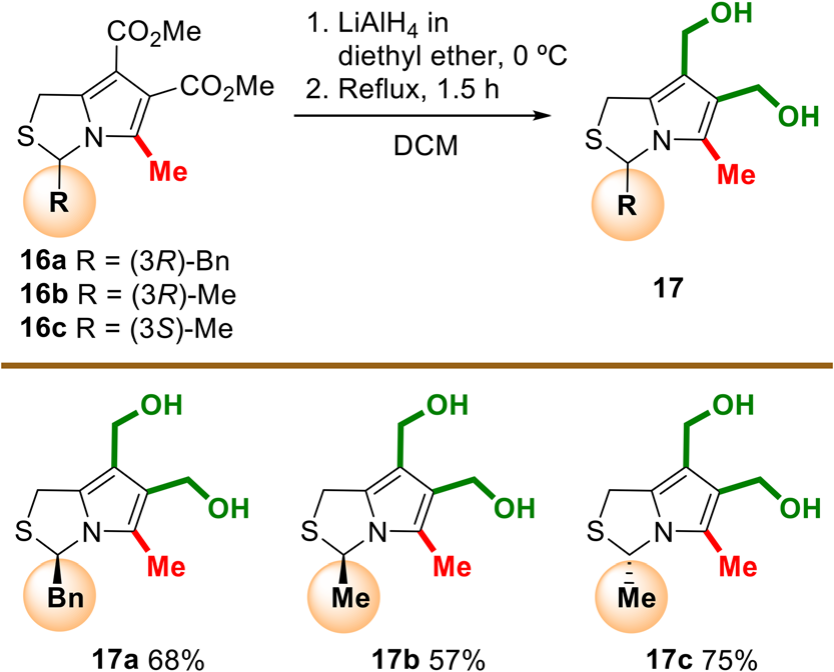
Synthesis of chiral 3-methyl- and 3-benzyl-6,7-bis(hydroxymethyl)-1*H*,3*H*-pyrrolo[1,2-*c*]thiazoles.

6,7-Bis(hydroxymethyl)-1*H*,3*H*-pyrrolo[1,2-*c*]thiazoles 17 bearing methyl or benzyl groups at position 3 were also prepared from the corresponding 1*H*,3*H*-pyrrolo[1,2-*c*]thiazole-6,7-dicarboxylates 16^14,15^ as outlined in Scheme 5. The target chiral molecules 17 were obtained in yields ranging from 57 to 75%.

### Antiproliferative effect on CRC cells of MANIO-like derivatives

**Fig. 2:**
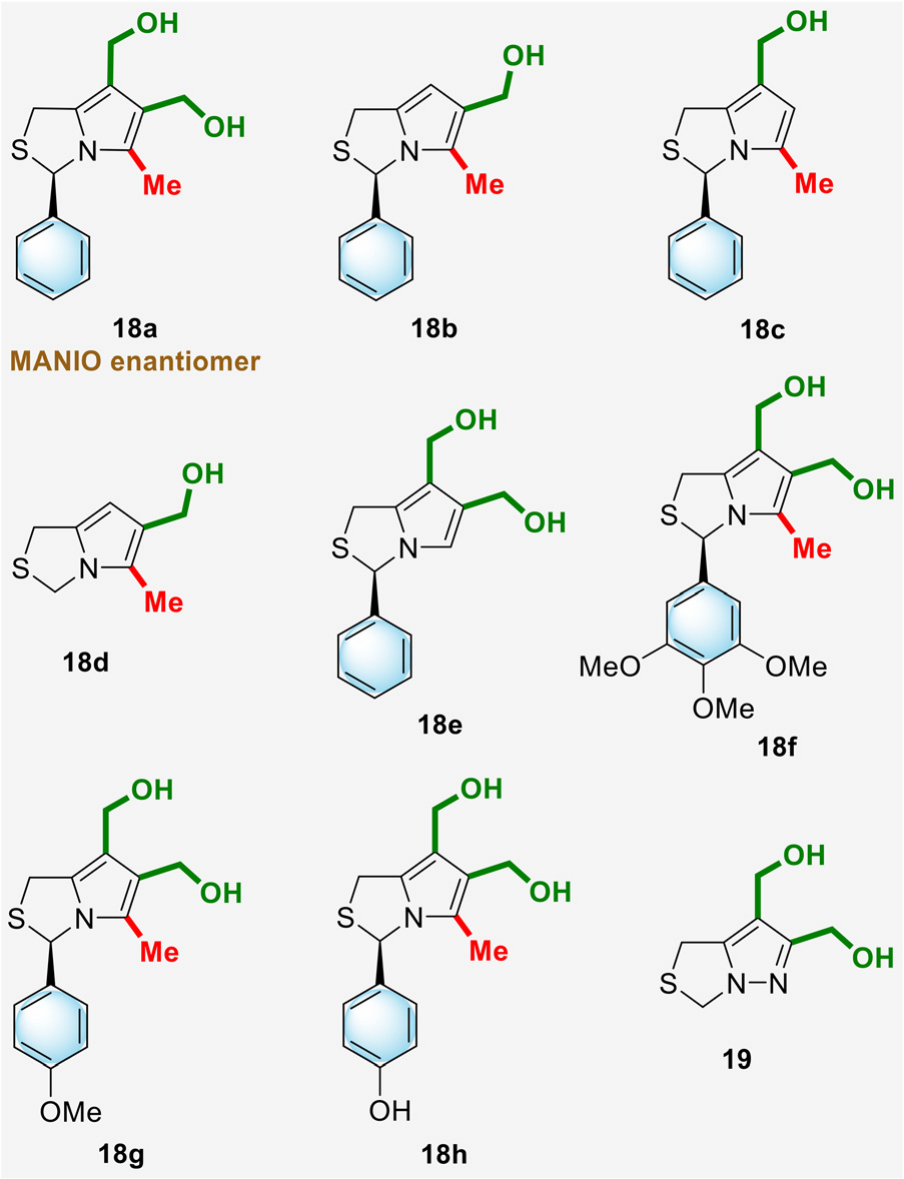
Chemical structures of previously synthesized 1*H*,3*H*-pyrrolo[1,2-*c*]thiazoles and 1*H*,3*H*-pyrazolo[1,5-*c*]thiazole.^10,11,16^

A small library of hydroxymethyl- and bis(hydroxymethyl)-1*H*,3*H*-pyrrolo[1,2-*c*]thiazoles and a bis(hydroxymethyl)-1*H*,3*H*-pyrazolo[1,5-*c*]thiazole were screened for p53-dependent anticancer activity. These included 1*H*,3*H*-pyrrolo[1,2-*c*]thiazoles, the synthesis of which has been described above, as well as hydroxymethyl- and bis(hydroxymethyl)-1*H*,3*H*-pyrrolo[1,2-*c*]thiazoles 18 and 6,7-bis(hydroxymethyl)-1*H*,3*H*-pyrazolo[1,5-*c*]thiazole 19 previously described by our research group (Fig. 2).^10,11,16^

*In vitro* studies regarding the antiproliferative activity of compounds 18 and 19 have been carried out in a panel of human colorectal cancer cells with different p53 status, WTp53-expressing HCT116 colon cells (HCT116 p53^+/+^) and p53 null isogenic derivative (HCT116 p53^−/−^). The comparison of the activity of the compounds was made by analyzing the corresponding IC_50_ values (Table 1). 1*H*,3*H*-Pyrrolo[1,2-*c*]thiazole 18a, the enantiomer of MANIO, was characterized by its high selectivity for HCT116 colon cells expressing p53 HCT116 p53^+/+^ with IC_50_ = 5.11 μM, whereas the IC_50_ value obtained for the p53 null isogenic derivative (HCT116 p53^−/−^) was higher than 50 μM. Nevertheless, the IC_50_ value for 18a against HCT116 p53^+/+^ was 5-fold higher than that obtained for MANIO in the same cells. Compound 18b, containing a hydroxymethyl group at C-6, and 6,7-bis(hydroxymethyl) derivative 18g, containing a *p*-methoxyphenyl group at position 3, both with *R*-configuration, also showed a marked selectivity to p53-expressing cancer cells, with IC_50_ values of 6.68 and 4.61 μM, respectively, compared to p53-null cells (>50 μM). Among the other compounds tested, none of them showed relevant selectivity for the WTp53-expressing HCT116 colon cells. Compound 18c, which differs from 18b only in the position of the hydroxymethyl group, shows a marked loss of selectivity for HCT116 colon cells expressing p53 HCT116 p53^+/+^. A similar loss of selectivity was observed for compound 18d, which differs from 18b only in having no substituents at C-3. Compound 18e, without substituents at the 5-position, showed a loss of selectivity of the same order of magnitude compared to compound 18a. Although the introduction of a *p*-methoxyphenyl group at position 3 resulted in good performance, as observed for compound 18g, the introduction of a bulkier trimethoxyphenyl group (compound 18f) resulted in a complete loss of activity against HCT116 colon cells. On the other hand, compound 18h, with a *p*-hydroxyphenyl group at C-3, showed lower selectivity and an IC_50_ against HCT116 p53^+/+^ 3-fold higher than that observed for 18a. Finally, compound 19 with a 1*H*,3*H*-pyrazolo[1,5-*c*]thiazole core showed no activity against HCT116 colon cells.

**Table 1:** IC_50_ values of compounds 18 and 19 in WTp53-expressing HCT116 colon cells (HCT116 p53^+/+^) and p53-null isogenic derivative (HCT116 p53^−/−^)

Compound	IC_50_ (μM)	
	HCT116 p53^+/+^	HCT116 p53^−/−^
1 (MANIO)	0.97 ± 0.04	48.25 ± 1.97
18a (MANIO enantiomer)	5.11 ± 0.42	51.83 ± 2.73
18b	6.68 ± 1.22	>50
18c	29.34 ± 2.08	>50
18d	28.32 ± 3.51	>50
18e	20.30 ± 1.53	>50
18f	>50	>50
18g	4.61 ± 0.59	>50
18h	16.65 ± 2.31	>50
19	>50	>50

**Fig. 3:**
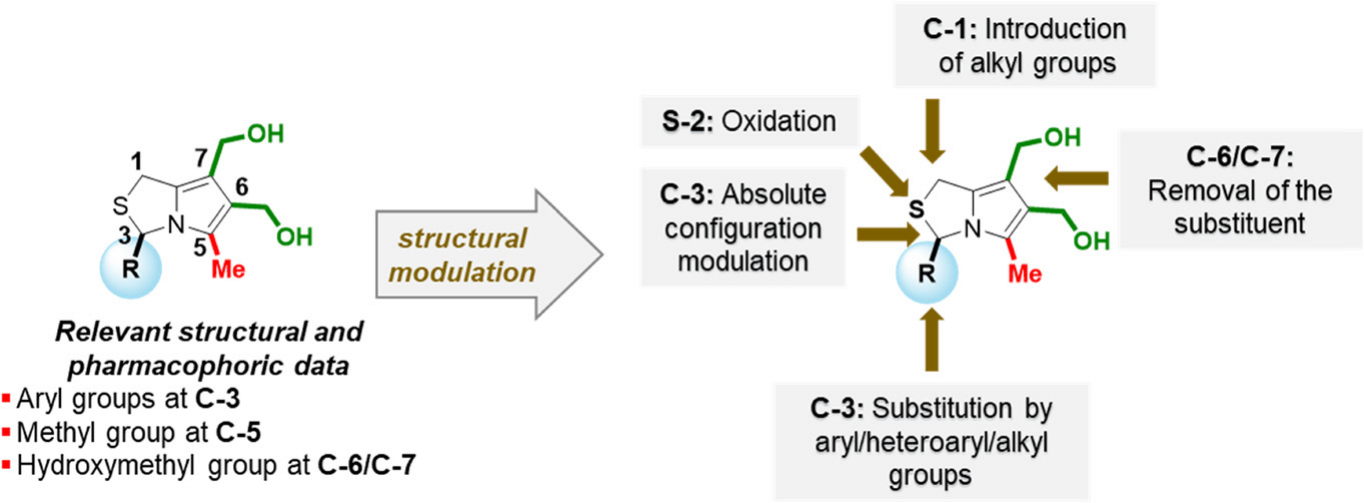
Structural modulation approaches based on SAR data.

SAR data allowed the identification of potential pharmacophores in the molecule and the definition of new MANIO-like derivatives to be studied (Fig. 3). It has been shown that the presence of an aryl group at C-3, a methyl group at C-5 and the presence of an hydroxymethyl group at position 6 may be crucial to ensure good antiproliferative activity in HCT116 colon cells expressing WTp53. Thus, the design of new 1*H*,3*H*-pyrazolo[1,5-*c*]thiazoles involved the structural modulation at position 3 through the introduction of fluorine containing aromatic substituents, naphthyl or quinolinyl groups. Among the new synthesized compounds, enantiomers of the most promising compounds studied (compounds 18b and 18g) were also prepared, as well as the sulfone of the enantiomer of MANIO. To confirm the importance of an aryl group at C-3 in ensuring good anticancer activity, the replacement of this type of functional group by alkyl groups was also carried out, resulting in novel 3-alkylated-6,7-bis(hydroxymethyl)-1*H*,3*H*-pyrrolo[1,2-*c*]thiazole derivatives.

**Fig. 4:**
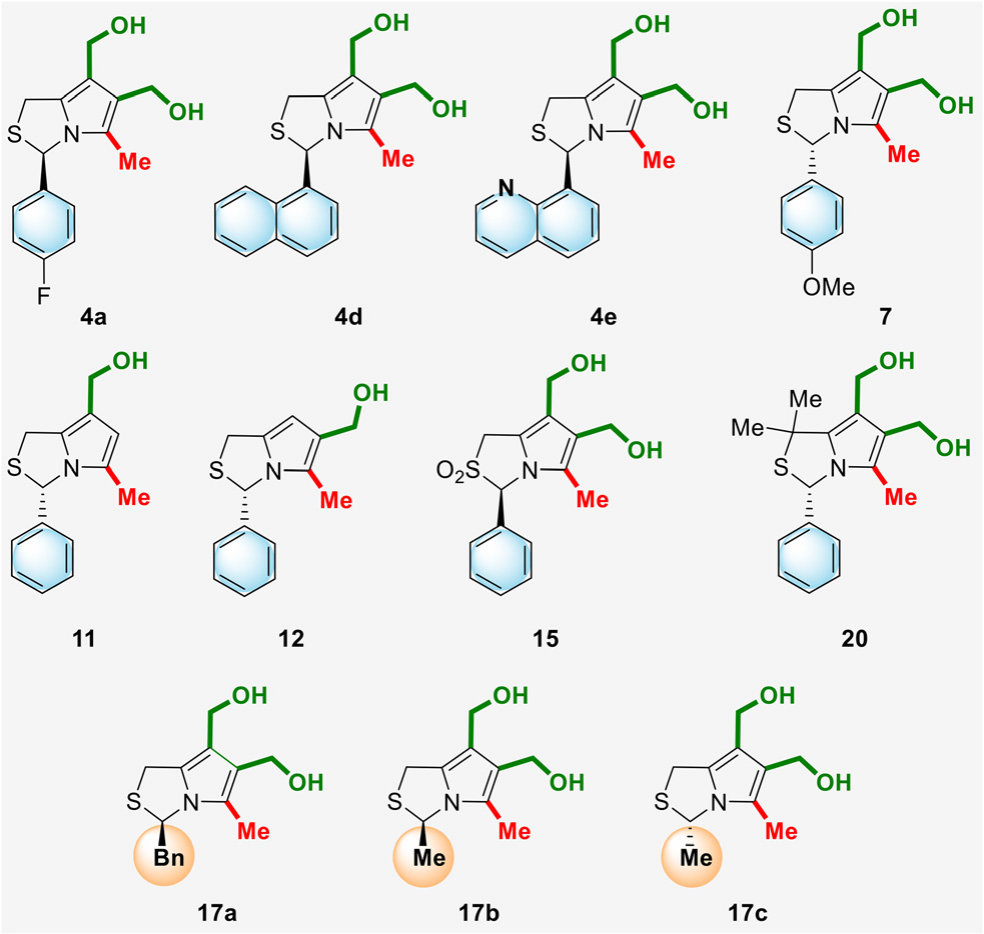
Chemical structures of new chiral hydroxymethyl- and bis(hydroxymethyl)-1*H*,3*H*-pyrrolo[1,2-*c*]thiazoles.

The antiproliferative activity of the newly synthesized 1*H*,3*H*-pyrrolo[1,2-*c*]thiazoles 4, 7, 11, 12, 15 and 17 as well as compound 20, whose synthesis was previously reported by our group,^10^ was evaluated in a panel of human colorectal cancer cells with different p53 status (Fig. 4, Table 2). (3*R*)-6,7-Bis(hydroxymethyl)-1*H*,3*H*-pyrrolo[1,2-*c*]thiazole 4a with a *p*-fluorophenyl substituent at position 3 showed good selectivity for HCT116 p53^+/+^ with an IC_50_ of 4.43 μM in contrast to the IC_50_ of 32.50 μM against HCT116 p53^−/−^ cells. Compound 4e, containing a quinolinyl group at C-3, had a slightly higher IC_50_ value in the HCT116 p53^+/+^ cells (7.50 μM) than 4a, however it showed higher selectivity for the p53 pathway, with the IC_50_ value obtained in HCT116 p53^−/−^ cells higher than 50 μM. Compound 15, the sulfone of the enantiomer of MANIO, showed higher IC_50_ values than MANIO enantiomer (compound 18a, Table 1), indicating that sulfur oxidation did not lead to an improvement of the anticancer activity. Compound 20, which differs from MANIO only by the presence of two methyl groups at position 1, showed moderate anticancer activity in HCT116 p53^+/+^ cells (IC_50_ = 24.34 μM) and no dependence on the p53 pathway (HCT116 p53^−/−^; IC_50_ = 22.15 μM).

**Table 2:** IC_50_ values of compounds 4a, 4d, 4e, 7, 11, 12, 15, 17 and 20 in WTp53-expressing HCT116 colon cells (HCT116 p53^+/+^) and p53-null isogenic derivative (HCT116 p53^−/−^)

Compound	IC_50_ (μM)	
	HCT116 p53^+/+^	HCT116 p53^−/−^
4a	4.43 ± 2.13	32.50 ± 1.30
4d	ND	ND
4e	7.50 ± 1.10	>50
7	1.47 ± 0.66	23.50 ± 2.50
11	0.14 ± 0.07	18.11 ± 1.62
12	0.99 ± 0.05	4.33 ± 1.10
15	>15	>50
17a	16.27 ± 1.15	29.11 ± 1.00
17b	33.28 ± 1.73	46.19 ± 2.31
17c	31.40 ± 3.51	42.73 ± 3.61
20	24.34 ± 2.86	22.15 ± 4.17

3-Alkyl-1*H*,3*H*-pyrrolo[1,2-*c*]thiazoles 17 showed moderate p53 dependence, with IC_50_ values in HCT116 p53^+/+^ (16–33 μM) only about 10 μM lower than those in p53 null isogenic cells (29–46 μM). As mentioned above, MANIO interacts with the WTp53 DBD protein backbone through hydrogen bonds between its hydroxyl groups and the amide backbone groups of the methionine residues of two different monomers, and through stacking interactions made with the bases of the DNA.^5^ This is in agreement with the results obtained for the alkylated derivatives, in particular for compounds 17b and 17c, where these aromatic stacking interactions are not possible.

The IC_50_ value of compound 7 in HCT116 p53^+/+^ (1.47 μM) was 3-fold lower than that obtained for its enantiomer, compound 18g (see Table 1). However, in contrast to its enantiomer, it showed lower selectivity for HCT116 p53^−/−^ cells (IC_50_ = 23.50 μM).

Mono-hydroxymethyl derivatives, regioisomers 11 and 12, showed excellent activity for HCT116 p53^+/+^ cells, with IC_50_ values of 0.11 and 0.99 μM, respectively. Remarkably, the IC_50_ value of 11 is about 200-fold lower than that obtained for its enantiomer (compound 18c, Table 1) but 7-fold lower than that obtained for MANIO. However, compounds 11 and 12 showed a low p53-dependent anti-proliferative effect with IC_50_ values of 18.11 and 4.33 μM for HCT116 p53^−/−^ cells, respectively.

### Antiproliferative effect on MDA-MB-231, PANC-1 and A375 cells of MANIO-like derivatives

The antiproliferative activity of the most active compounds against HCT116 colon cells (HCT116 p53^+/+^) was then evaluated in a panel of different human cancer cell lines, triple-negative breast cancer (MDA-MB-231), pancreatic adenocarcinoma (PANC-1) and melanoma (A375) (Table 3). However, none of the compounds showed relevant activity in any of the cell lines tested. Of note is the moderate activity of MANIO, its enantiomer and compound 12 in the human melanoma cell line (IC_50_ < 15 μM).

**Table 3:** IC_50_ values of most active compounds in HCT116 p53^+/+^ cells in PANC-1, A375 and MDA-MB-231 cell lines

Compound	IC_50_^a^ (μM)		
	MDA-MB-231	PANC-1	A375
1 (MANIO)	>50	42.15 ± 2.32	13.20 ± 0.71
18a (MANIO enantiomer)	>50	33.10 ± 1.27	14.49 ± 1.85
18b	>50	>50	39.50 ± 1.41
18g	>50	>50	>50
4a	>50	20.15 ± 2.76	36.14 ± 1.32
4e	>50	17.35 ± 2.48	24.35 ± 2.62
7	16.57 ± 1.86	>50	22.80 ± 0.91
12	24.11 ± 2.09	35.00 ± 2.57	11.78 ± 1.84
11	>50	>50	>50

a Values correspond to the media ± standard deviation of 3 independent experiments.

## Conclusion

Structural modulation of the lead compound MANIO was performed resulting in the synthesis of thirteen new 1*H*,3*H*-pyrrolo[1,2-*c*]thiazoles with *R* and *S* configuration. The antiproliferative activity of a library of twenty compounds was assessed in a panel of human colorectal cancer cells with different p53 status, providing relevant structural and pharmacophoric information.

Among the compounds tested, five, including the enantiomer of MANIO, all with aromatic or heteroaromatic substituents at C-3, showed selectivity towards p53-expressing cancer cells, with IC_50_ values in cancer cells expressing WTp53 (HCT116 p53^+/+^) ranging between 1.47 and 7.50 μM and IC_50_ > 23.50 μM in p53-null isogenic derivatives (HCT116 p53^−/−^). It was observed that the *p*-methoxyphenyl-substituted derivative with *S*-configuration showed significantly higher activity against HCT116 p53^+/+^ cells than its enantiomer, 1.47 *vs.* 4.61 μM.

Mono-hydroxymethyl derivatives with *S*-configuration stood out for their high antiproliferative activity in colon cancer cells expressing WTp53, with IC_50_ < 1 μM, higher activity than the corresponding enantiomers and MANIO, but no p53-dependent growth-inhibitory effect.

Collectively, these results indicate that the presence of aromatic substituents at C-3 and a methyl group at C-5 are essential to ensure effective p53-dependent growth inhibitory activity. Furthermore, the absolute configuration at the chiral center was shown to play an important role in the antiproliferative activity in WTp53-expressing colon cancer cells. It was also observed that the position of the hydroxymethyl group in mono-hydroxymethyl derivatives affects also the inhibitory activity. Interestingly, while for 1*H*,3*H*-pyrrolo[1,2-*c*]thiazoles with *R* configuration the 6-hydroxymethyl derivative was significantly more active than the 7-hydroxymethyl derivative, in the case of 1*H*,3*H*-pyrrolo[1,2-*c*]thiazoles with *S* configuration, both 6-hydroxymethyl and 7-hydroxymethyl derivative show outstanding antiproliferative activity in WTp53-expressing HCT116 colon cells, but p53-independent inhibitory activity.

Overall, the present work represents a successful strategy of modulation and rational structural optimization of a lead molecule, which allowed to gather pertinent structural and pharmacophoric data for the design of new p53-activating agents for precision therapy of CRC or other cancers harboring WT or mutp53.

## Experimental

### Chemistry

Thin-layer chromatography (TLC) analyses were performed using precoated silica gel plates. Column chromatography was performed with silica gel 60 as the stationary phase. ^1^H Nuclear magnetic resonance (NMR) spectra (400 MHz), ^13^C NMR spectra (100 MHz) and ^19^F spectra (376 MHz) were recorded in CDCl3, CD_3_OD or hexadeuterated dimethylsulfoxide (DMSO-*d*_6_). Chemical shifts are expressed in parts per million (ppm) relatively to internal tetramethylsilane (TMS), and coupling constants (*J*) are in hertz. Infrared (IR) spectra were recorded in a Fourier transform spectrometer using either a KBr matrix or diamond attenuated total reflectance (ATR) mode. Elemental analyses were carried out with an Elemental Vario Micro Cube analyser. High-resolution mass spectra (HRMS) were obtained on a TOF VG Autospect M spectrometer with electron impact (EI), on a Thermo Orbitrap Q-Exactive Focus spectrometer with electrospray ionization (ESI) or on a Bruker MicroTOF (APCI-FIA-TOF). Melting points were determined in open glass capillaries and are uncorrected. Optical rotations were measured on an Optical Activity AA-5 electrical polarimeter.

Thiazolidine-4-carboxylic acids 2a,^8^2c,^17^2d,^18^2f,^16^*N*-acetyl-thiazolidine 8,^10^ 1*H*,3*H*-pyrrolo[1,2-*c*]thiazoles 16,^14,15^ hydroxymethyl- and bis(hydroxymethyl)-1*H*,3*H*-pyrrolo[1,2-*c*]thiazoles 18,^10,11,16^ bis(hydroxymethyl)-1*H*,3*H*-pyrazolo[1,5-*c*]thiazole 19,^10^ and 1*H*,3*H*-pyrrolo[1,2-*c*]thiazole-2,2-dioxide 13^13^ were prepared as described in the literature.

#### General procedure for the synthesis of 1,3-thiazolidine-4-carboxylic acids 2

A solution of the corresponding aldehyde (29.0 mmol) in ethanol (22 mL) was added to a solution of l-cysteine (3.51 g, 29.0 mmol) in water (22 mL). The reaction was stirred for 5 h, the product was filtered and washed with diethyl ether.

##### 2-(4-(Trifluoromethoxy)phenyl)-1,3-thiazolidine-4-carboxylic acid (2b)

Obtained from 4-(trifluoromethoxy)benzaldehyde (5.51 g, 29.0 mmol) as described in the general procedure. Yield: 75% (6.39 g), white solid, mp 146.6–147.6 °C. The ^1^H NMR spectrum obtained at 25 °C showed the presence of the two diastereoisomers (2*R*,4*R*) and (2*S*,4*R*) (ratio 40 : 60); ^1^H NMR (400 MHz, DMSO-*d*_6_): *δ* = 3.08–3.14 (2 × m, 2H), 3.32 and 3.38 (2 × dd, *J* = 10.2, 7.0 Hz and *J* = 10.0, 7.0 Hz, 2H), 3.92 and 4.18 (2 × dd, *J* = 8.8, 7.0 Hz and *J* = 6.8, 5.1 Hz, 2H), 5.56 and 5.74 (2 × s, 2H), 7.32 and 7.36 (2 × d, *J* = 8.1 Hz and *J* = 8.1 Hz, ArH, 4H), 7.56–7.58 and 7.66–7.68 (2 × m, ArH, 4H). ^13^C NMR (100 MHz, DMSO-*d*_6_): *δ* = 38.0 and 38.3, 64.8 and 65.6, 69.9 and 70.6, 120.0 (q,^1^*J*_C–F_ = 254 Hz), 120.7 and 120.9, 128.8 and 129.4, 138.6 and 141.2, 147.5 and 148.0, 172.0 and 172.8. ^19^F NMR (376 MHz, DMSO-*d*_6_): *δ* = −56.79 and −56.78. IR (ATR): *ν* = 1572, 1509, 1437, 1381, 1251, 1208, 1163 cm^−1^. HRMS (ESI): calcd for C_11_H_11_NO_3_F_3_S [M + H]^+^ 294.0406; found 294.0401.

##### 2-(Quinolin-8-yl)thiazolidine-4-carboxylic acid (2e)

Obtained from quinoline-8-carbaldehyde (2.50 g, 15.9 mmol) as described in the general procedure. Yield: 95% (3.96 g), white solid, mp 129.8–131.1 °C. The ^1^H NMR spectrum obtained at 25 °C showed the presence of the two diastereoisomers (2*R*,4*R*) and (2*S*,4*R*) (ratio 50 : 50); ^1^H NMR (400 MHz, CD_3_OD): *δ* = 3.44–3.49 (2 × m, 2H), 3.59–3.68 (2 × m, 2H), 4.32 and 4.57 (2 × pseudo-t, *J* = 7.6 Hz and *J* = 6.5 Hz, 2H), 6.52 and 6.72 (2 × s, 2H), 7.58–7.66 (m, ArH, 4H), 7.95–8.02 (m, ArH, 4H), 8.39–8.42 (m, ArH, 2H), 8.96–8.98 (m, ArH, 2H). ^13^C NMR (100 MHz, DMSO-*d*_6_): *δ* = 37.3, 38.4, 65.3, 65.8, 66.2, 67.9, 121.4, 121.6, 124.6, 126.2, 126.3, 127.1, 127.4, 127.9, 130.0, 128.2, 136.3, 136.7, 140.2, 144.9, 145.2, 149.2, 149.6, 172.4, 172.9. IR (ATR): *ν* = 1606, 547, 1500, 1397, 1359, 1314 cm^−1^. HRMS (ESI): calcd for C_13_H_13_N_2_O_2_S [M + H]^+^ 261.0692; found 261.0688.

##### (2*S*,4*R*)-3-Acetyl-2-(4-methoxyphenyl)thiazolidine-4-carboxylic acid (5)

The compound was prepared according to a procedure described in the literature for the synthesis of (2*S*,4*R*)-3-acetyl-2-phenylthiazolidine-4-carboxylic acid.^10^ Yield: 61%, white solid, mp 148.4–149.9 °C (from ethanol). The ^1^H NMR spectrum obtained at 25 °C showed the presence of the two rotational isomers; ^1^H NMR (400 MHz, CD_3_OD): *δ* = 1.76 and 2.01 (2 × s, 3H), 3.15–3.47 (2 × m, 2H), 3.73 and 3.76 (2 × s, 3H), 5.10 and 5.32 (2 × d, *J* = 6.9 Hz and *J* = 6.0 Hz, 1H), 6.12 and 6.33 (2 × s, 1H), 6.83 and 6.92 (d, *J* = 8.7 Hz, 2H), 7.19 and 7.24 (d, *J* = 8.7 Hz, 2H). IR (ATR): *ν* = 1706, 1597, 1513, 1403, 1243, 1182, 1030 cm^−1^. HRMS (ESI): calcd for C_13_H_16_NO_4_S [M + H]^+^ 282.0795; found 282.0792. [*α*]^20^_D_ = −270 (*c* 0.5, DCM).

#### General procedure for the synthesis of 1*H*,3*H*-pyrrolo[1,2-*c*]thiazole carboxylates

A solution of the appropriate thiazolidine-4-carboxylic acid (18.0 mmol), dipolarophile (1.5 or 1.8 equiv.) and Ac_2_O (60 mL) was heated at 110 °C for the time indicated. The reaction was cooled to room temperature and was diluted with CH_2_Cl_2_ (150 mL). The organic phase was washed with saturated aqueous solution of NaHCO_3_ and with water, dried (Na_2_SO_4_) and the solvent evaporated off. The crude product was purified by column chromatography [hexane–ethyl acetate].

##### Dimethyl (3*R*)-3-(4-fluorophenyl)-5-methyl-1*H*,3*H*-pyrrolo[1,2-*c*]thiazole-6,7-dicarboxylate (3a)

Obtained from thiazolidine 2a (2.02 g, 8.8 mmol) and DMAD (1.88 g, 13.2 mmol, 1.5 equiv.) as described in the general procedure. Reaction time: 12 h. Purification by column chromatography [hexane/EtOAc (1 : 2), (1 : 1), then (2 : 1)] gave compound 3a as solid (2.30 g, 69%). Recrystallization from EtOAc/hexane gave the compound as a pale-yellow solid, mp 101.8–102.3 °C. ^1^H NMR (400 MHz, CDCl_3_): *δ* = 2.00 (s, 3H), 3.83 (s, 1H), 3.83 (s, 1H), 4.32 (d, *J* = 15.0 Hz, 1H), 4.47 (dd, *J* = 15.0, 1.6 Hz, 1H), 6.28 (d, *J* = 1.6 Hz, 1H), 7.03–7.08 (m, 4H). ^13^C NMR (100 MHz, CDCl_3_): *δ* = 11.4, 30.0, 51.4, 51.6, 64.4, 107.0, 116.3 (d,^2^*J*_C–F_ = 22.9 Hz), 117.6, 127.6 (d,^3^*J*_C–F_ = 8.4 Hz), 130.5, 136.0 (d,^4^*J*_C–F_ = 3.2 Hz), 140.3, 162.8 (d,^1^*J*_C–F_ = 247 Hz), 164.0, 165.2. ^19^F NMR (376 MHz, CDCl_3_): *δ* = −114.47. IR (ATR): *ν* = 2952, 1773, 1699, 1508, 1436, 1291, 1219, 1150, 1090 cm^−1^. HRMS (ESI): calcd for C_17_H_17_NO_4_FS [M + H]^+^ 350.0857; found 350.0853. [*α*]^20^_D_ = +185 (*c* 1, CH_2_Cl_2_).

##### Dimethyl (3*R*)-5-methyl-3-(4-(trifluoromethoxy)phenyl)-1*H*,3*H*-pyrrolo[1,2-*c*]thiazole-6,7-dicarboxylate (3b)

Obtained from thiazolidine 2b (2.00 g, 6.8 mmol) and DMAD (1.45 g, 10.2 mmol, 1.5 equiv.) as described in the general procedure. Reaction time: 12 h. Purification by column chromatography [hexane/EtOAc (1 : 1)] gave compound 3b as a colorless oil (1.85 g, 65%). ^1^H NMR (400 MHz, CDCl_3_): *δ* = 2.02 (s, 3H), 3.83 (s, 6H), 4.33 (d, *J* = 15.0 Hz, 1H), 4.47 (dd, *J* = 15.0, 1.6 Hz, 1H), 6.29 (d, *J* = 1.5 Hz, 1H), 7.06–7.12 (m, 2H), 7.20 (d, *J* = 8.2 Hz, 2H). ^13^C NMR (100 MHz, CDCl_3_): *δ* = 11.5, 30.0, 51.5, 51.6, 64.1, 107.1, 116.5, 120.3 (d,^1^*J*_C–F_ = 256 Hz), 121.7, 127.2, 130.5, 138.9, 140.3, 149.4 (d,^3^*J*_C–F_ = 1.5 Hz), 163.9, 165.2. ^19^F NMR (376 MHz, CDCl_3_): *δ* = −57.88. IR (ATR): *ν* = 2952, 1702, 1509, 1439, 1252, 1208, 1155, 1092 cm^−1^. HRMS (ESI): calcd for C_18_H_17_NO_5_F_3_S [M + H]^+^ 416.0774; found 416.0770. [*α*]^20^_D_ = +107 (*c* 0.5, CH_2_Cl_2_).

##### Dimethyl (3*R*)-5-methyl-3-(4-(trifluoromethyl)phenyl)-1*H*,3*H*-pyrrolo[1,2-*c*]thiazole-6,7-dicarboxylate (3c)

Obtained from thiazolidine 2c (2.00 g, 7.2 mmol) and DMAD (1.54 g, 10.8 mmol, 1.5 equiv.) as described in the general procedure. Reaction time: 4 h. Purification by column chromatography [hexane/EtOAc (3 : 1)] gave compound 3c as an oil (1.44 g, 50%). Recrystallization from EtOAc/hexane gave the compound as white solid, mp 105.6–106.6 °C. ^1^H NMR (400 MHz, CDCl_3_): *δ* = 2.03 (s, 3H), 3.83 (s, 3H), 3.84 (s, 3H), 4.34 (d, *J* = 14.9 Hz, 1H), 4.47 (dd, *J* = 1.5 and 14.9, 1H), 6.32 (d, *J* = 1.2 Hz, 1H), 7.16 (d, *J* = 8.1 Hz, 2H), 7.62 (d, *J* = 8.2 Hz, 2H). ^13^C NMR (100 MHz, CDCl_3_): *δ* = 11.5, 30.1, 51.5, 51.6, 64.1, 107.3, 117.8, 123.7 (q,^1^*J*_C–F_ = 272 Hz), 125.9, 126.4 (q,^3^*J*_C–F_ = 3.7 Hz), 130.4, 131.2 (q,^2^*J*_C–F_ = 32.7 Hz), 140.3, 144.2, 163.9, 165.1. ^19^F NMR (376 MHz, CDCl_3_): *δ* = −62.79. IR (ATR): *ν* = 2950, 1705, 1437, 1323, 1204, 1126, 1090, 1065 cm^−1^. HRMS (ESI): calcd for C_18_H_17_NO_4_F_3_S [M + H]^+^ 400.0825; found 400.0822. [*α*]^20^_D_ = +180 (*c* 0.5, CH_2_Cl_2_).

##### Dimethyl (3*R*)-5-methyl-3-(naphthalen-1-yl)-1*H*,3*H*-pyrrolo[1,2-*c*]thiazole-6,7-dicarboxylate (3d)

Obtained from thiazolidine 2d (2.00 g, 7.7 mmol) and DMAD (1.64 g, 11.6 mmol, 1.5 equiv.) as described in the general procedure. Reaction time: 4 h. Purification by column chromatography [hexane/EtOAc (3 : 1), then hexane/EtOAc (2 : 1)] gave compound 3d as a solid (1.03 g, 35%). Recrystallization from EtOAc/hexane gave the compound as pale-yellow solid, mp 192.9–194.8 °C. ^1^H NMR (400 MHz, CDCl_3_): *δ* = 2.08 (s, 3H), 3.85 (s, 6H), 4.36 (s, 2H), 6.51 (bs, 1H), 6.98 (s, 1H), 7.34 (pseudo-t, *J* = 7.7 Hz, 1H), 7.55–7.62 (m, 2H), 7.82 (d, *J* = 8.3 Hz, 1H), 7.88–7.93 (m, 2H). ^13^C NMR (100 MHz, CDCl_3_): *δ* = 11.5, 29.8, 51.4, 51.6, 61.1, 107.5, 117.2, 121.0, 122.1, 125.5, 126.4, 127.1, 129.3, 129.3, 131.1, 134.0, 135.4, 141.1, 164.0, 165.3. IR (ATR): *ν* = 2947, 1726, 1698, 1534, 1433, 1204, 1154, 1093 cm^−1^. HRMS (ESI): calcd for C_21_H_20_NO_4_S [M + H]^+^ 382.1108; found 382.1103. [*α*]^20^_D_ = +396 (*c* 0.5, CH_2_Cl_2_).

##### Dimethyl (3*R*)-5-methyl-3-(quinolin-8-yl)-1*H*,3*H*-pyrrolo[1,2-*c*]thiazole-6,7-dicarboxylate (3e)

Obtained from thiazolidine 2e (2.01 g, 7.7 mmol) and DMAD (1.64 g, 11.6 mmol, 1.5 equiv.) as described in the general procedure. Reaction time: 4 h. Purification by column chromatography [hexane/EtOAc (1 : 1)] gave compound 3e as a solid (1.35 g, 46%). Recrystallization from EtOAc/hexane gave the compound as white solid, mp 203.9–205.8 °C (with decomposition). ^1^H NMR (400 MHz, CDCl_3_): *δ* = 2.10 (s, 3H), 3.85 (s, 3H), 3.86 (s, 3H), 4.31 (d, *J* = 15.1 Hz, 1H), 4.36 (dd, *J* = 0.8 and 15.1 Hz, 1H), 6.79 (d, *J* = 7.0 Hz, 1H), 7.43 (pseudo-t, *J* = 7.7 Hz, 1H), 7.50 (dd, *J* = 4.2 and 8.3 Hz, 1H), 7.54 (br s, 1H), 7.80 (dd, *J* = 1.1 and 8.2 Hz, 1H), 8.20 (dd, *J* = 1.7 and 8.3 Hz, 1H), 8.97 (dd, *J* = 1.7 and 4.2 Hz, 1H). ^13^C NMR (100 MHz, CDCl_3_): *δ* = 11.5, 29.5, 51.4, 51.6, 60.1, 107.3, 117.0, 121.8, 123.8, 126.4, 128.5, 131.0, 136.3, 138.2, 141.5, 144.5, 150.0, 164.1, 165.4. IR (ATR): *ν* = 2946, 1725, 1696, 1534, 1444, 1385, 1303, 1204, 1156, 1092 cm^−1^. HRMS (ESI): calcd for C_20_H_19_N_2_O_4_S [M + H]^+^ 383.1060; found 383.1057. [*α*]^20^_D_ = +434 (*c* 0.5, CH_2_Cl_2_).

##### Dimethyl (3*S*)-3-(4-methoxyphenyl)-5-methyl-1*H*,3*H*-pyrrolo[1,2-*c*]thiazole-6,7-dicarboxylate (6)

Obtained from *N*-acetyl-thiazolidine 5 (0.81 g, 2.9 mmol) and DMAD (0.61 g, 4.3 mmol, 1.5 equiv.) as described in the general procedure. Reaction time: 12 h. Purification by column chromatography [hexane/EtOAc (1 : 1)] gave compound 6 as a solid (0.72 g, 69%). Recrystallization from EtOAc/hexane gave the compound as pale-yellow solid, mp 86.3–88.1 °C. ^1^H NMR (400 MHz, CDCl_3_): *δ* = 2.00 (s, 3H), 3.80 (s, 3H), 3.82 (s, 3H), 3.83 (s, 3H), 4.30 (d, *J* = 14.9 Hz, 1H), 4.47 (dd, *J* = 1.7 and 14.9, 1H), 6.27 (d, *J* = 1.5 Hz, 1H), 6.85–6.87 (m, 2H), 7.01–7.03 (m, 2H). ^13^C NMR (100 MHz, CDCl_3_): *δ* = 11.4, 30.0, 51.4, 51.6, 55.4, 64.8, 106.7, 114.6, 117.4, 127.2, 130.8, 131.9, 140.4, 160.1, 164.1, 165.5. IR (ATR): *ν* = 2958, 1687, 1513, 1447, 1230, 1169, 1091, 1027 cm^−1^. HRMS (ESI): calcd for C_18_H_20_NO_5_S [M + H]^+^ 362.1057; found 362.1053. [*α*]^20^_D_ = −183 (*c* 0.5, CH_2_Cl_2_).

##### Methyl (3*S*)-5-methyl-3-phenyl-1*H*,3*H*-pyrrolo[1,2-*c*]thiazole-7-carboxylate (9) and methyl (3*S*)-5-methyl-3-phenyl-1*H*,3*H*-pyrrolo[1,2-*c*]thiazole-6-carboxylate (10)

Obtained from *N*-acetyl-thiazolidine 8 (2.00 g, 7.96 mmol) and methyl propiolate (1.20 g, 14.33 mmol, 1.8 equiv.) as described in the general procedure. Reaction time: 16 h. Purification by column chromatography [hexane/EtOAc (2 : 1)] gave a mixture of 9 and 10 (50 : 50) (1.46 g, 67%). Compound 10 could be separated by selective crystallization with diethyl ether–hexane. *Methyl (3S)-5-methyl-3-phenyl-1H,3H-pyrrolo[1,2-c]thiazole-6-carboxylate* (10): pale-yellow solid, mp 89.7–91.7 °C. ^1^H NMR (400 MHz, CDCl_3_): *δ* = 2.18 (s, 3H), 3.78 (s, 3H), 4.02 (d, *J* = 13.1 Hz, 1H), 4.27 (d, *J* = 13.1 Hz, 1H), 6.25 (s, 1H), 6.33 (s, 1H), 7.01 (dd, *J* = 1.7 and 7.7 Hz, 2H), 7.29–7.35 (m, 3H). ^13^C NMR (100 MHz, CDCl_3_): *δ* = 11.8, 28.2, 50.8, 64.0, 101.7, 116.6, 125.4, 128.6, 129.1, 132.0, 133.2, 141.1, 165.8. IR (ATR): *ν* = 2923, 1688, 1523, 1363, 1222, 1169, 1068 cm^−1^. HRMS (ESI): calcd for C_15_H_16_NO_2_S [M + H]^+^ 274.0896; found 274.0892. [*α*]^20^_D_ = −320 (*c* 0.5, CH_2_Cl_2_). *Methyl (3S)-5-methyl-3-phenyl-1H,3H-pyrrolo[1,2-c]thiazole-7-carboxylate* (9): ^1^H NMR (400 MHz, CDCl_3_): *δ* = 1.82 (s, 3H), 3.81 (s, 3H), 4.34 (d, *J* = 14.7 Hz, 1H), 4.51 (dd, *J* = 1.8 and 14.7 Hz), 6.27 (d, *J* = 1.8 Hz, 1H), 6.36 (s, 1H), 7.06 (dd, *J* = 1.7 and 7.6 Hz, 2H), 7.30–7.35 (m, 3H). ^13^C NMR (100 MHz, CDCl_3_): *δ* = 12.1, 30.4, 51.0, 65.0, 106.9, 112.2, 125.7, 125.8, 128.8, 129.1, 140.3, 140.4, 165.2.

#### General procedure for the synthesis of hydroxymethyl-1*H*,3*H*-pyrrolo[1,2-*c*]thiazoles

A solution containing the appropriate 1*H*,3*H*-pyrrolo[1,2-*c*]thiazole (2.30 mmol) in dry dichloromethane (30 mL) was added dropwise to a suspension of lithium aluminium hydride (2.2 equiv., 192 mg, 5.06 mmol) in anhydrous diethyl ether at 0 °C, unless otherwise stated. After completing the addition, the reaction mixture was refluxed under nitrogen for 1.5 h and then cooled on an ice bath. The excess of hydride was carefully decomposed by addition of ethyl acetate followed by slow addition of water (0.2 mL), NaOH 15% (0.2 mL) and water (0.6 mL). The mixture was filtered through celite and the inorganic residue was washed with several portions of hot dichloromethane. The filtrate was dried (Na_2_SO_4_) and the solvent evaporated off. The crude product was purified by column chromatography [hexane–ethyl acetate] or recrystallisation and stored under nitrogen at −18 °C.

##### (3*R*)-6,7-Bis(hydroxymethyl)-3-(4-fluorophenyl)-5-methyl-1*H*,3*H*-pyrrolo[1,2-*c*]thiazole (4a)

Obtained from compound 3a (1.06 g, 2.80 mmol) as described in the general procedure. Purification by column chromatography [hexane/EtOAc (1 : 2), then hexane/EtOAc (1 : 3)] followed by trituration with diethyl ether gave compound 4a as a pale yellow solid (290 mg, 34%). mp ≥ 85 °C, with decomposition. ^1^H NMR (400 MHz, CDCl_3_): *δ* = 1.83 (s, 3H), 2.99 (bs, 2H), 4.07 (d, *J* = 12.8 Hz, 1H), 4.27 (d, *J* = 12.8 Hz, 1H), 4.45 (d, *J* = 12.3 Hz, 1H), 4.49 (d, *J* = 12.3 Hz, 1H), 4.55 (s, 2H), 6.21 (d, *J* = 0.8 Hz, 1H), 6.97–7.06 (m, 4H). ^13^C NMR (100 MHz, CDCl_3_): *δ* = 10.0, 27.6, 56.2, 56.5, 63.7, 113.5, 116.0 (d,^2^*J*_C–F_ = 21.9 Hz), 122.9, 123.7, 127.6 (d,^3^*J*_C–F_ = 8.4 Hz), 131.4, 137.5 (d,^4^*J*_C–F_ = 3.1 Hz), 162.6 (d,^1^*J*_C–F_ = 248 Hz). ^19^F NMR (376 MHz, CDCl_3_): *δ* = −113.04. IR (ATR): *ν* = 3326, 2863, 1604, 1508, 1354, 1226, 997 cm^−1^. HRMS (APCI): calcd for C_15_H_15_FNOS [M − OH]^+^ 276.0853; found 276.0855. [*α*]^20^_D_ = +221 (*c* 0.5, CH_2_Cl_2_).

##### (3*R*)-6,7-Bis(hydroxymethyl)-5-methyl-3-(4-(trifluoromethoxy)phenyl)-1*H*,3*H*-pyrrolo[1,2-*c*]thiazole (4b)

Obtained from compound 3b (1.72 g, 4.15 mmol) as described in the general procedure. Purification by column chromatography [hexane/EtOAc (1 : 2), then hexane/EtOAc (1 : 3)] gave compound 4b (60 mg, 4%) in a very impure form.

##### (3*R*)-6,7-Bis(hydroxymethyl)-5-methyl-3-(4-(trifluoromethyl)phenyl)-1*H*,3*H*-pyrrolo[1,2-*c*]thiazole (4c)

Obtained from compound 3c (0.93 g, 2.34 mmol) as described in the general procedure. Purification by column chromatography [hexane/EtOAc (1 : 2), hexane/EtOAc (1 : 5), then EtOAc] gave compound 4c in trace amount and in a very impure form.

##### (3*R*)-6,7-Bis(hydroxymethyl)-5-methyl-3-(naphthalen-1-yl)-1*H*,3*H*-pyrrolo[1,2-*c*]thiazole (4d)

Obtained from compound 3d (0.47 g, 1.15 mmol) as described in the general procedure. Purification by column chromatography [hexane/EtOAc (1 : 2), hexane/EtOAc (1 : 5), then EtOAc] gave compound 4d as a white solid (255 mg, 53%). mp ≥ 180 °C, with decomposition (triturated from diethyl ether). ^1^H NMR (400 MHz, CDCl_3_): *δ* = 1.92 (s, 3H), 2.68 (bs, 2H), 4.08 (d, *J* = 12.5 Hz, 1H), 4.22 (d, *J* = 12.8 Hz, 1H), 4.55 (d, *J* = 12.0 Hz, 1H), 4.60 (d, *J* = 12.2 Hz, 1H), 4.63 (s, 2H), 6.51 (bs, 1H), 6.91 (s, 1H), 7.33 (pseudo-t, *J* = 7.7 Hz, 1H), 7.53–7.60 (m, 2H), 7.79 (d, *J* = 8.2 Hz, 1H), 7.90 (d, *J* = 7.9 Hz, 2H). IR (ATR): *ν* = 3297, 2869, 1541, 1431, 1353, 979, 777 cm^−1^. HRMS (APCI): calcd for C_19_H_18_NOS [M − OH]^+^ 308.1104; found 308.1106. [*α*]^20^_D_ = +540 (*c* 0.5, CH_2_Cl_2_).

##### (3*R*)-6,7-Bis(hydroxymethyl)-5-methyl-3-(quinolin-8-yl)-1*H*,3*H*-pyrrolo[1,2-*c*]thiazole (4e)

Obtained from compound 3e (1.24 g, 3.24 mmol) as described in the general procedure. Recrystallization of the crude product from EtOAc/hexane followed by trituration with diethyl ether gave the compound 4e as a white solid (0.80 g, 75%). mp 148.3–150.1 °C. ^1^H NMR (400 MHz, CDCl_3_): *δ* = 1.95 (s, 3H), 2.52 (bs, 2H), 4.03 (d, *J* = 13.0 Hz, 1H), 4.20 (d, *J* = 13.0 Hz, 1H), 4.57 (d, *J* = 12.4 Hz, 1H), 4.61 (d, *J* = 12.4 Hz, 1H), 4.63 (d, *J* = 12.8 Hz, 1H), 4.66 (d, *J* = 12.8 Hz, 1H), 6.76 (d, *J* = 7.1 Hz, 1H), 7.39–7.43 (m, 1H), 7.44 (s, 1H), 7.48 (dd, *J* = 4.2 and 8.3, Hz, 1H), 7.76 (dd, *J* = 0.9 and 8.2 Hz, 1H), 8.18 (dd, *J* = 1.7 and 8.3 Hz, 1H), 8.98 (dd, *J* = 1.7 and 4.2 Hz, 1H). ^13^C NMR (100 MHz, CDCl_3_): *δ* = 9.9, 27.1, 56.6, 56.9, 59.6, 113.8, 121.6, 123.0, 123.2, 124.0, 126.5, 128.0, 128.4, 132.3, 136.2, 139.6, 144.7, 149.8. IR (ATR): *ν* = 3207, 2917, 2850, 1498, 1422, 1361, 984, 798 cm^−1^. HRMS (ESI): calcd for C_18_H_19_N_2_O_2_S [M − H]^+^ 327.1162; found 327.1156. [*α*]^20^_D_ = +570 (*c* 1, CH_2_Cl_2_).

##### (3*S*)-6,7-Bis(hydroxymethyl)-3-(4-methoxyphenyl)-5-methyl-1*H*,3*H*-pyrrolo[1,2-*c*]thiazole (7)

Obtained from compound 6 (0.51 g, 1.42 mmol) as described in the general procedure. Purification by trituration with diethyl ether yields compound 7 as a white solid (323 mg, 74%). mp 113–115 °C. ^1^H NMR (400 MHz, CDCl_3_): *δ* = 1.83 (s, 3H), 2.43 (bs, 1H), 2.57 (bs, 1H), 3.79 (s, 3H), 4.07 (d, *J* = 12.8 Hz, 1H), 4.28 (d, *J* = 12.8 Hz, 1H), 4.48 (d, *J* = 12.3 Hz, 1H), 4.52 (d, *J* = 12.2 Hz, 1H), 4.58 (s, 2H), 6.21 (d, *J* = 0.9 Hz, 1H), 6.82–6.86 (m, 2H), 7.00–7.04 (m, 2H). ^13^C NMR (100 MHz, CDCl_3_): *δ* = 10.0, 27.6, 55.3, 56.4, 56.7, 64.2, 113.3, 114.3, 123.1, 123.5, 127.2, 131.5, 133.4, 159.7. IR (ATR): *ν* = 3361, 2866, 1613, 1541, 1255, 1171, 1008, 819 cm^−1^. Anal. Calcd for C_16_H_19_NO_3_S: C, 62.93; H, 6.27; N, 4.59; S, 10.50. Found: C, 62.80; H, 6.11; N, 4.44; S, 10.81. [*α*]^20^_D_ = −280 (*c* 0.5, CH_2_Cl_2_).

##### (3*S*)-7-Hydroxymethyl-5-methyl-3-phenyl-1*H*,3*H*-pyrrolo[1,2-*c*]thiazole (11) and (3*S*)-6-hydroxymethyl-5-methyl-3-phenyl-1*H*,3*H*-pyrrolo[1,2-*c*]thiazole (12)

Obtained from the crude mixture of 9 and 10 [ratio (1 : 1), from the reaction of *N*-acetyl-thiazolidine 8 (6.16 mmol) and methyl propiolate, as described previously] and LiAlH_4_ (6.16 mmol), as described in the general procedure. In this case the reaction mixture was refluxed for 4 h after the addition was completed. Purification by column chromatography [hexane/EtOAc (2 : 1), then hexane/EtOAc (1 : 1)] gave in order of elution (3*S*)-7-hydroxymethyl-5-methyl-3-phenyl-1*H*,3*H*-pyrrolo[1,2-*c*]thiazole (11) (159 mg, 10.5%) and (3*S*)-6-hydroxymethyl-5-methyl-3-phenyl-1*H*,3*H*-pyrrolo[1,2-*c*]thiazole (12) (247 mg, 16%). *(3S)-7-Hydroxymethyl-5-methyl-3-phenyl-1H,3H-pyrrolo[1,2-c]thiazole* (11). White solid. mp 91–93 °C (triturated with diethyl ether). ^1^H NMR (400 MHz, CDCl_3_): *δ* = 1.36 (bs, 1H),1.83 (s, 3H), 4.09 (d, *J* = 12.8 Hz, 1H), 4.31 (d, *J* = 12.8 Hz, 1H), 4.52 (pseudo-t, *J* = 12.2 Hz, 2H), 5.98 (s, 1H), 6.22 (s, 1H), 6.90–7.14 (m, 2H), 7.28–7.35 (m, 3H). ^13^C NMR (100 MHz, CDCl_3_): *δ* = 12.1, 27.9, 58.3, 64.3, 111.3, 114.1, 125.1, 125.7, 128.4, 129.0, 131.8, 141.7. IR (ATR): *ν* = 3376, 2907, 1438, 1377, 1344, 1004, 990, 708 cm^−1^. HRMS (ESI): calcd for C_14_H_15_NOSNa [M + Na]^+^ 268.0767; found 268.0761. [*α*]^20^_D_ = −280 (*c* 0.5, CH_2_Cl_2_). *(3S)-6-Hydroxymethyl-5-methyl-3-phenyl-1H,3H-pyrrolo[1,2-c]thiazole* (12). Pale pink solid. mp 61–63 °C (triturated with diethyl ether). ^1^H NMR (400 MHz, CDCl_3_): *δ* = 1.26 (bs, 1H), 1.87 (s, 3H,), 4.05 (d, *J* = 12.8 Hz, 1H), 4.30 (d, *J* = 12.8 Hz, 1H), 4.45 (s, 2H), 5.93 (s, 1H), 6.22 (d, *J* = 1.4 Hz, 1H), 7.03–7.05 (m, 2H), 7.28–7.32 (m, 3H). ^13^C NMR (100 MHz, CDCl_3_): *δ* = 10.0, 28.8, 58.2, 64.2, 100.1, 122.9, 124.9, 125.7, 128.4, 129.0, 133.0, 141.9. IR (ATR): *ν* = 3363, 2899, 1337, 1110, 986, 695 cm^−1^. HRMS (APCI): calcd for C_14_H_14_NS [M − OH]^+^ 228.0841; found 228.0843. [*α*]^20^_D_ = −300 (*c* 0.5, CH_2_Cl_2_).

##### (3*S*)-6-Hydroxymethyl-5-methyl-3-phenyl-1*H*,3*H*-pyrrolo[1,2-*c*]thiazole (12)

Obtained from compound 10 (0.45 g, 1.64 mmol) and LiAlH_4_ (3.94 mmol) as described in the general procedure. In this case the reaction mixture was refluxed for 4 h after the addition was completed. Purification by column chromatography [hexane/EtOAc (1 : 1)] followed by trituration with diethyl ether yields compound 12 as a pale pink solid (221 mg, 55%). Compound 12 was identified by comparison with the specimen previously prepared (see above).

##### (3*R*)-6,7-Bis(hydroxymethyl)-3-benzyl-5-methyl-1*H*,3*H*-pyrrolo[1,2-*c*]thiazole (17a)

Obtained from compound 16a (0.63 g, 1.83 mmol) as described in the general procedure. Purification by column chromatography [hexane/EtOAc (1 : 3), then hexane/EtOAc (1 : 5)] gave compound 17a as a colorless oil (363 mg, 68%). ^1^H NMR (400 MHz, CDCl_3_): *δ* = 2.27 (s, 3H), 2.62 (bs, 2H), 3.15–3.21 (m, 2H), 3.52 (d, *J* = 12.9 Hz, 1H), 3.65 (d, *J* = 12.9 Hz, 1H), 4.40 (s, 2H), 4.50 (d, *J* = 12.6 Hz, 1H), 4.54 (d, *J* = 12.7 Hz, 1H), 5.41 (t, *J* = 4.9 Hz, 1H), 7.03–7.05 (m, 2H), 7.23–7.25 (m, 3H). ^13^C NMR (100 MHz, CDCl_3_): *δ* = 10.1, 26.8, 44.5, 56.3, 56.5, 63.5, 113.2, 121.9, 123.2, 127.2, 128.2, 130.0, 131.5, 135.5. IR (ATR): *ν* = 3306, 2913, 2861,1429, 1355, 1237, 1030, 978, 696 cm^−1^. HRMS (APCI): calcd for C_16_H_18_NOS [M − OH]^+^ 272.1104; found 272.1107. [*α*]^20^_D_ = +200 (*c* 1, CH_2_Cl_2_).

##### (3*R*)-6,7-Bis(hydroxymethyl)-3,5-dimethyl-1*H*,3*H*-pyrrolo[1,2-c]thiazole (17b)

Obtained from compound 16b (1.21 g, 4.50 mmol) as described in the general procedure. Purification by trituration with diethyl ether yields compound 17b as a white solid (549 mg, 57%). mp 65–66 °C. ^1^H NMR (400 MHz, CDCl_3_): *δ* = 1.70 (d, *J* = 6.1 Hz, 1H), 2.23 (s, 3H), 2.44 (bs, 2H), 3.92 (d, *J* = 12.9 Hz, 1H), 4.21 (d, *J* = 12.9 Hz, 1H), 4.48–4.55 (m, 4H), 5.33 (q, *J* = 5.9 Hz, 1H). ^13^C NMR (100 MHz, CDCl_3_): *δ* = 10.0, 25.7, 26.7, 56.3, 56.6, 58.1, 113.2, 122.0, 123.0, 130.6. IR (ATR): *ν* = 3272, 2913, 2858, 1528, 1437, 1367, 1325, 1241, 971 cm^−1^. HRMS (EI): calcd for C_10_H_15_NO_2_S [M]^+^ 213.0824; found 213.0828. [*α*]^20^_D_ = +110 (*c* 1, CH_2_Cl_2_).

##### (3*S*)-6,7-Bis(hydroxymethyl)-3,5-dimethyl-1*H*,3*H*-pyrrolo[1,2-*c*]thiazole (17c)

Obtained from compound 16c (1.12 g, 4.17 mmol) as described in the general procedure. Purification by trituration with diethyl ether yields compound 17c as a white solid (669 mg, 75%). mp 65–66 °C. ^1^H NMR (400 MHz, CDCl_3_): *δ* = 1.70 (d, *J* = 6.2 Hz, 1H), 2.23 (s, 3H), 2.69 (bs, 2H), 3.91 (d, *J* = 12.9 Hz, 1H), 4.20 (d, *J* = 12.9 Hz, 1H), 4.46–4.52 (m, 4H), 5.33 (q, *J* = 5.9 Hz, 1H). ^13^C NMR (100 MHz, CDCl_3_): *δ* = 10.0, 25.7, 26.7, 56.3, 56.6, 58.1, 113.2, 122.0, 123.0, 130.6. IR (KBr): *ν* = 3286, 2913, 2877, 1529, 1442, 1327, 985 cm^−1^. HRMS (APCI): calcd for C_10_H_14_NOS [M − OH]^+^ 196.0791; found 196.0791. [*α*]^20^_D_ = −110 (*c* 1, CH_2_Cl_2_).

#### Synthesis of hydroxymethyl-1*H*,3*H*-pyrrolo[1,2-*c*]thiazole-2,2-dioxides 14 and 15

The synthesis was performed based on a procedure described in the literature.^19^ A suspension of compound 13 (0.37 g, 1.03 mmol) and finely powdered NaBH_4_ (16 equiv., 0.62 g, 16.48 mmol) in anhydrous THF (20 mL) was stirred for 20 min at 70 °C, followed by the dropwise addition of methanol (2.1 mL). The reaction mixture was left stirring for 24 h at 70 °C under nitrogen atmosphere. The reaction was cooled to room temperature, quenched by adding saturated aqueous NH_4_Cl solution (3 mL) and stirred for 1 h. The aqueous phase was extracted with ethyl acetate (3 × 20 mL), the organic extracts were dried (Na_2_SO_4_) and the solvent evaporated. Purification by column chromatography [hexane/EtOAc (1 : 3), hexane/EtOAc (1 : 5), then EtOAc] gave in order of elution methyl (3*R*)-7-hydroxymethyl-5-methyl-3-phenyl-1*H*,3*H*-pyrrolo[1,2-*c*]thiazole-6-carboxylate-2,2-dioxide (14) (107 mg, 31%) and (3*R*)-6,7-bis(hydroxymethyl)-5-methyl-3-phenyl-1*H*,3*H*-pyrrolo[1,2-*c*]thiazole-2,2-dioxide (15) (166 mg, 53%).

##### Methyl (3*R*)-7-hydroxymethyl-5-methyl-3-phenyl-1*H*,3*H*-pyrrolo[1,2-*c*]thiazole-6-carboxylate-2,2-dioxide (14)

mp 112–114 °C. ^1^H NMR (400 MHz, CDCl_3_): *δ* = 2.27 (s, 3H), 3.87 (s, 3H), 4.22 (d, *J* = 15.2 Hz, 1H), 4.48 (d, *J* = 15.2 Hz, 1H), 4.61 (d, *J* = 13.2 Hz, 1H), 4.69 (d, *J* = 13.2 Hz, 1H), 5.91 (s, 1H), 7.02–7.04 (m, 2H), 7.41–7.47 (m, 3H). ^13^C NMR (100 MHz, CDCl_3_): *δ* = 12.6, 48.2, 51.5, 57.1, 77.8, 113.6, 118.9, 122.2, 126.8, 129.6, 130.62. 130.64, 135.7, 166.2. IR (KBr): *ν* = 3132, 1712, 1672, 1454, 1404, 1317, 1138 cm^−1^. HRMS (EI): calcd for C_16_H_17_NO_5_S [M]^+^ 335.0827; found 335.0827. [*α*]^20^_D_ = 0.0 (*c* 1, CH_2_Cl_2_).

##### (3*R*)-6,7-Bis(hydroxymethyl)-5-methyl-3-phenyl-1*H*,3*H*-pyrrolo[1,2-*c*]thiazole-2,2-dioxide (15)

mp > 170 °C (with decomposition). ^1^H NMR (400 MHz, CDCl_3_): *δ* = 2.00 (s, 3H), 2.50 (bs, 1H), 2.77 (bs, 1H), 4.22 (d, *J* = 15.0 Hz, 1H), 4.43 (d, *J* = 15.0 Hz, 1H), 4.59–4.65 (m, 4H), 5.88 (s, 1H), 7.05–7.07 (m, 2H), 7.41–7.45 (m, 3H). ^13^C NMR (100 MHz, CDCl_3_): *δ* = 10.1, 48.4, 55.8, 56.6, 78.2, 118.2, 119.2, 121.6, 126.2, 127.1, 129.4, 130.4, 131.3. IR (KBr): *ν* = 3532, 3454, 2981, 2923, 2873, 1311, 1130, 987, 700 cm^−1^. HRMS (ESI): calcd for C_15_H_17_NO_4_SNa [M + Na]^+^ 330.0771; found 330.0764. [*α*]^20^_D_ = +20.0 (*c* 0.25, CH_2_Cl_2_).

### Biological evaluation

#### Human cell lines

Human colorectal adenocarcinoma HCT116 cell lines expressing p53 and its p53-null isogenic derivative (HCT116 p53^−/−^) were provided by B. Vogelstein (The JohnsHopkins Kimmel Cancer Center, Baltimore, MD, USA), triple-negative metastatic breast adenocarcinoma (MDA-MB-231), pancreatic ductal adenocarcinoma (PANC-1), and melanoma (A375) cell lines were purchased from ATCC (Rockville, MD, USA). HCT116 and A375 cell lines were cultured in monolayer in RPMI-1640 medium with 2 mM l-glutamine from Biowest (Biowest, VWR, Carnaxide, Portugal), supplemented with 10% heat-inactivated fetal bovine serum (FBS; Gibco, Alfagene, Lisboa, Portugal) and PANC-1 and MDA-MB-231, in DMEM high glucose (4.5 g L^−1^ glucose) with l-glutamine and sodium pyruvate, supplemented with 10% heat-inactivated FBS, at 37 °C, 5% CO_2_.

#### Sulforhodamine B (SRB) assay

5 × 10^3^ cells per well were seeded in 96-well plates for 24 h and then treated with serial dilutions of the appropriate compound ranging from 0.2 to 50 μM and incubated for 48 h. IC_50_ values for each cell line were determined by the sulforhodamine B (SRB) assay, as previously described.^20^

## Author contributions

MH, AMP, ABP, MS: chemical synthesis; CC, JR: cell biology studies; MS, LS, TPM: project conception and supervision; MS, LS, TPM: manuscript preparation.

## Conflicts of interest

One patent application protecting the compounds disclosed in this manuscript has been filed by the following authors M. I. L. S., L. S., and T. M. V. D. P. M.

## Supplementary Material

MD-015-D4MD00076E-s001
