# Supplementary material for: Chiral hydroxymethyl-1H,3H-pyrrolo[1,2-c]thiazoles: the search for selective p53-activating agents for colorectal cancer therapy
Source: RSC Med Chem. 2024 Apr 12;15(5):1652–63. doi: 10.1039/d4md00076e (PMC11110798; doi:10.1039/d4md00076e)
Supplement: MD-015-D4MD00076E-s001 [file MD-015-D4MD00076E-s001.pdf]

Electronic Supplementary Material (ESI) for RSC Medicinal Chemistry.
This journal is © The Royal Society of Chemistry 2024

Electronic Supporting Information

Chiral Hydroxymethyl-1*H*,3*H*-pyrrolo[1,2-*c*]thiazoles: the Search for Selective p53-Activating Agents for Colorectal Cancer Therapy

Mees M. Hendrikx,[a] Adelino M. R. Pereira,[a] Ana B. Pereira,[a] Carla S. C. Carvalho,[b] João L. P. Ribeiro,[a] Maria I. L. Soares,[a] Lucília Saraiva,[b,*] Teresa M. V. D. Pinho e Melo [a,*]

[a]University of Coimbra, Coimbra Chemistry Centre – Institute of Molecular Sciences and Department of Chemistry, 3004-535 Coimbra, Portugal.

[b]LAQV/REQUIMTE, Laboratório de Microbiologia, Departamento de Ciências Biológicas, Faculdade de Farmácia, Universidade do Porto, Porto, Portugal

Table of Contents

Copies of ^{1}H, ^{13}C and ^{19}F NMR Spectra for New Compounds **S3**

Copies of ^{1}H, ^{13}C and ^{19}F NMR Spectra for New Compounds

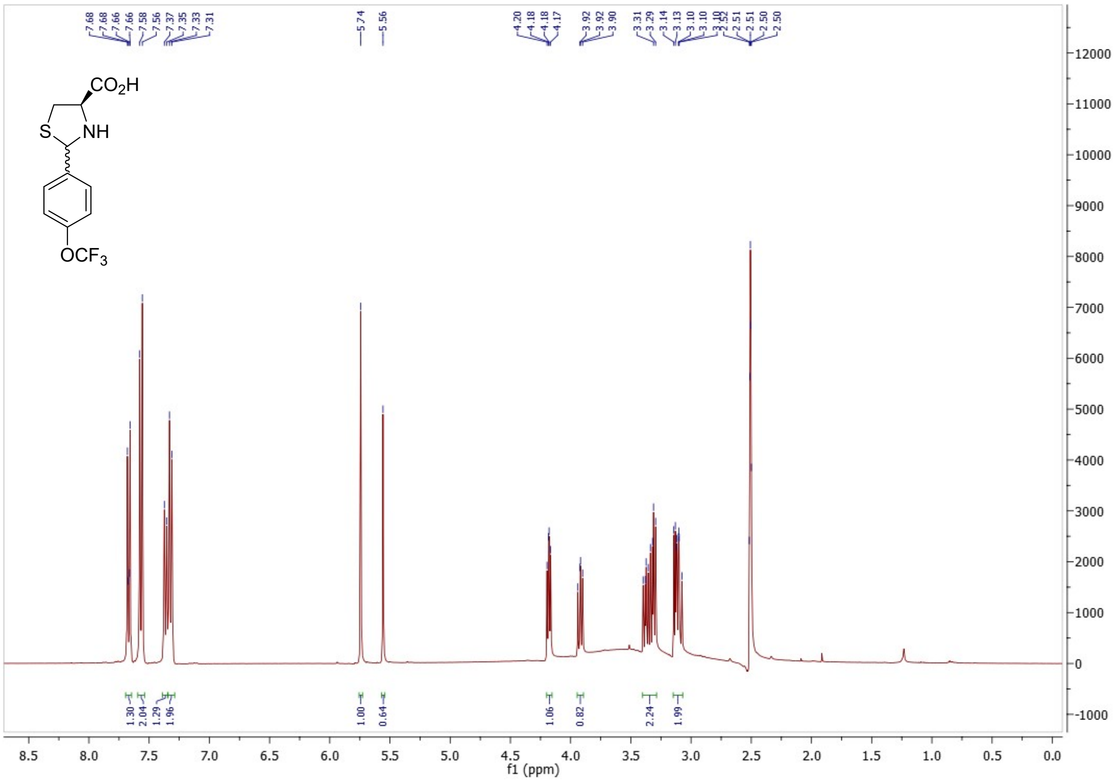

Figure S1: ^{1}H NMR spectrum of compound **2c** (400 MHz, DMSO-d_6).

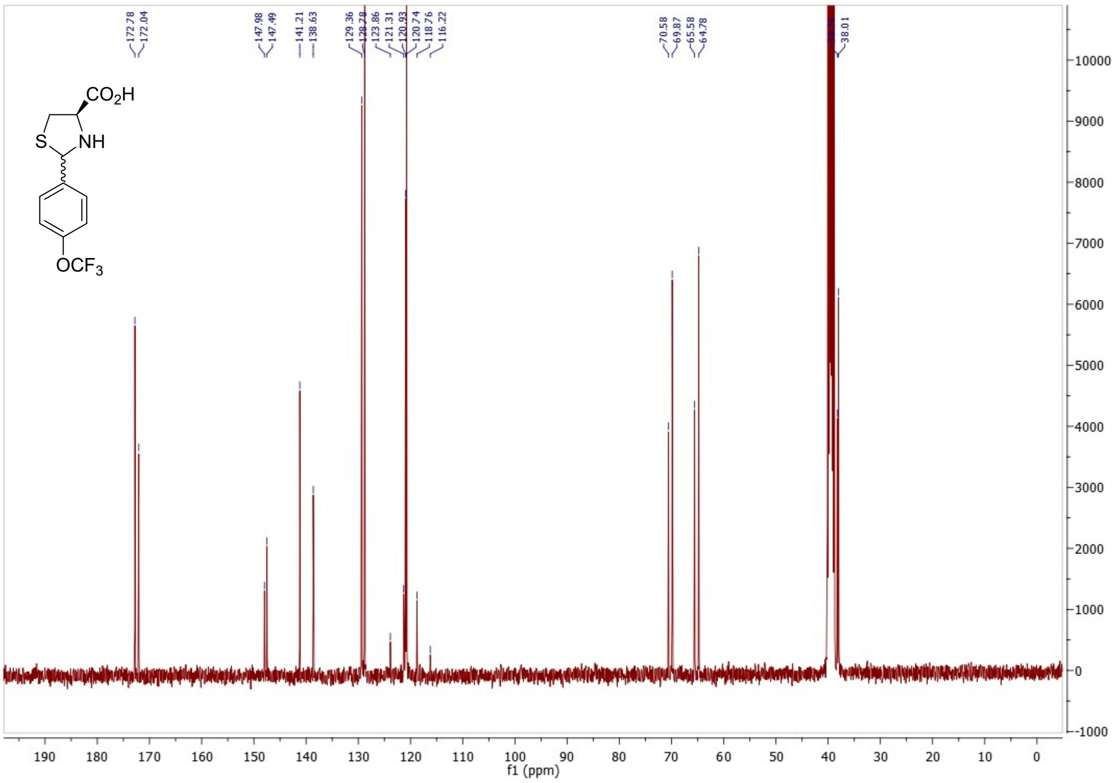

Figure S2: ^{13}C NMR spectrum of compound **2c** (100 MHz, DMSO-d_6).

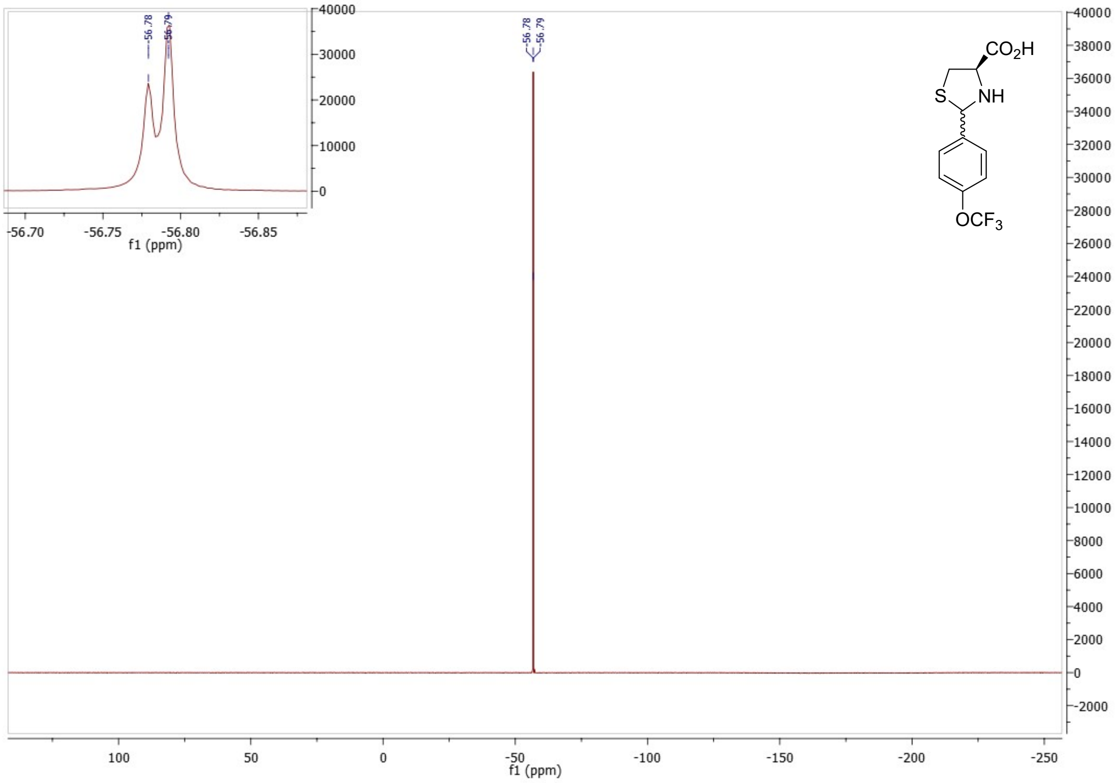

Figure S3: ^{19}F NMR spectrum of compound **2c** (376 MHz, DMSO-d_6).

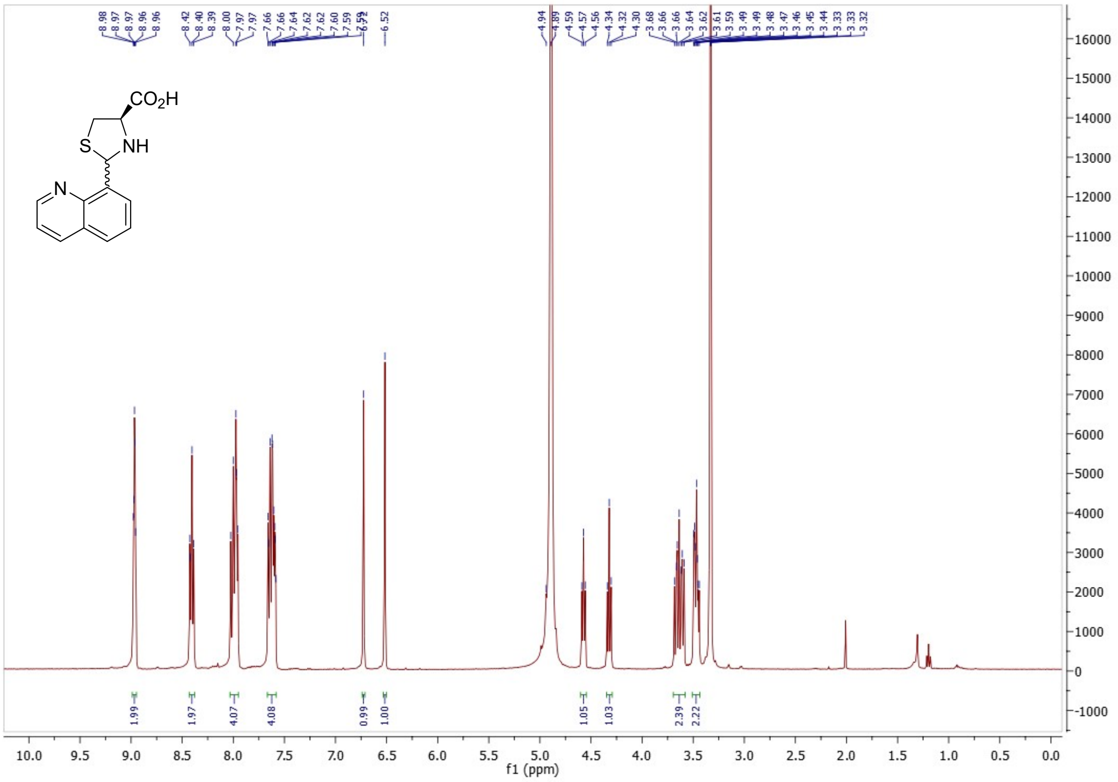

Figure S4: ^{1}H NMR spectrum of compound **2e** (400 MHz, CD_3OD).

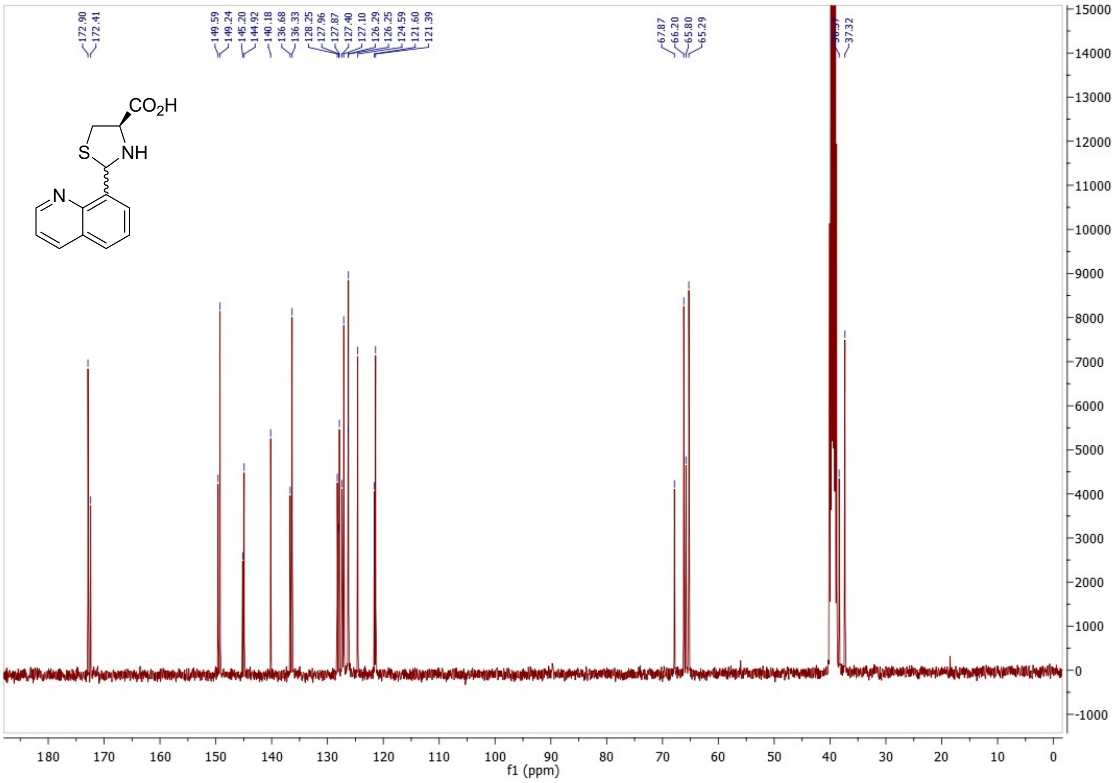

Figure S5: ^{13}C NMR spectrum of compound **2** (100 MHz, DMSO-d_6).

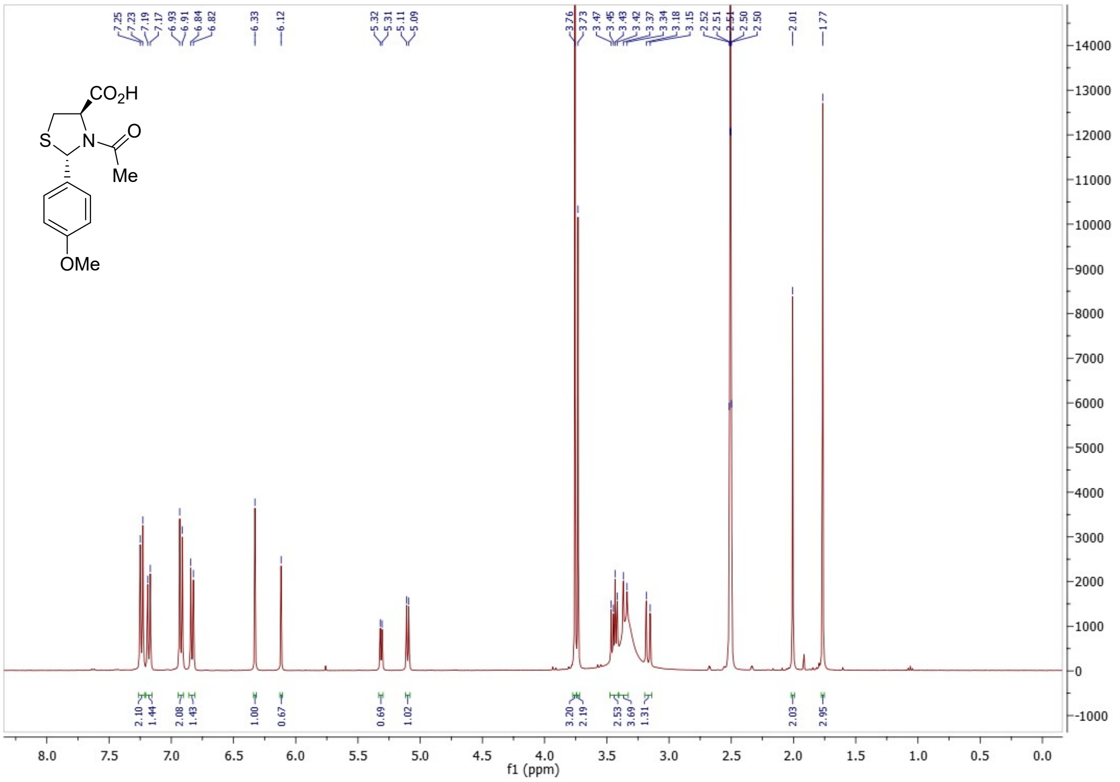

Figure S6: ^{1}H NMR spectrum of compound **5** (400 MHz, DMSO-d_6).

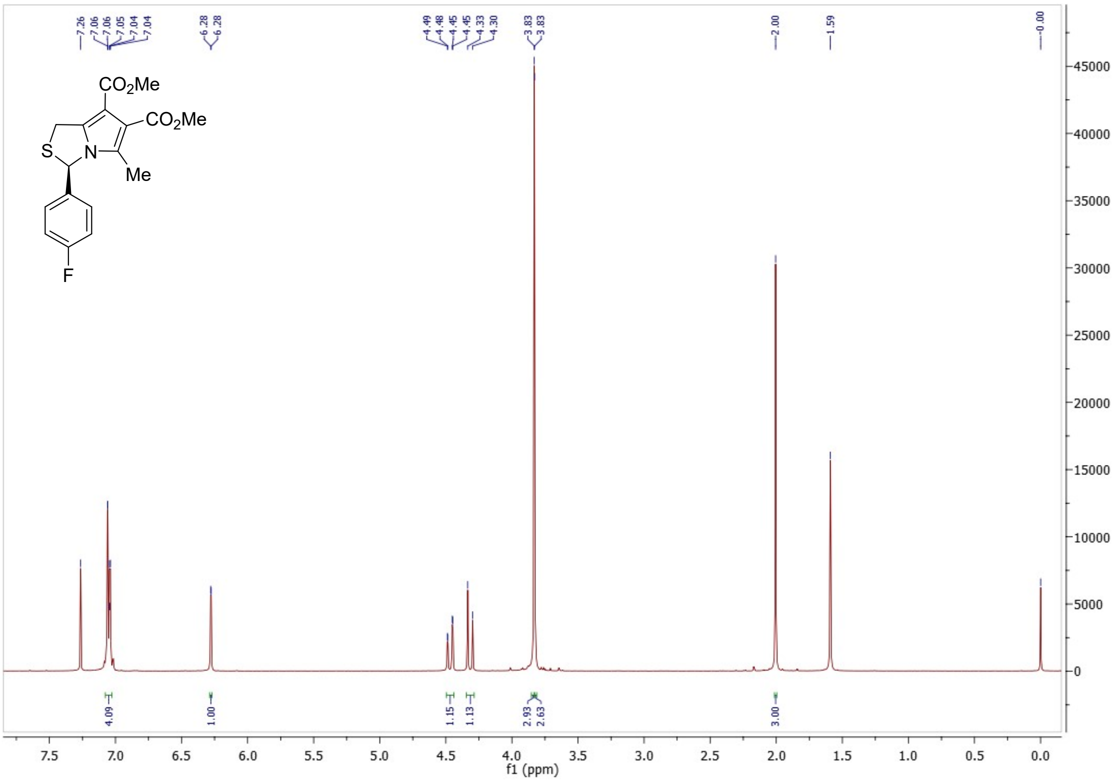

Figure S7: ^{1}H NMR spectrum of compound **3a** (400 MHz, $CDCl_3$).

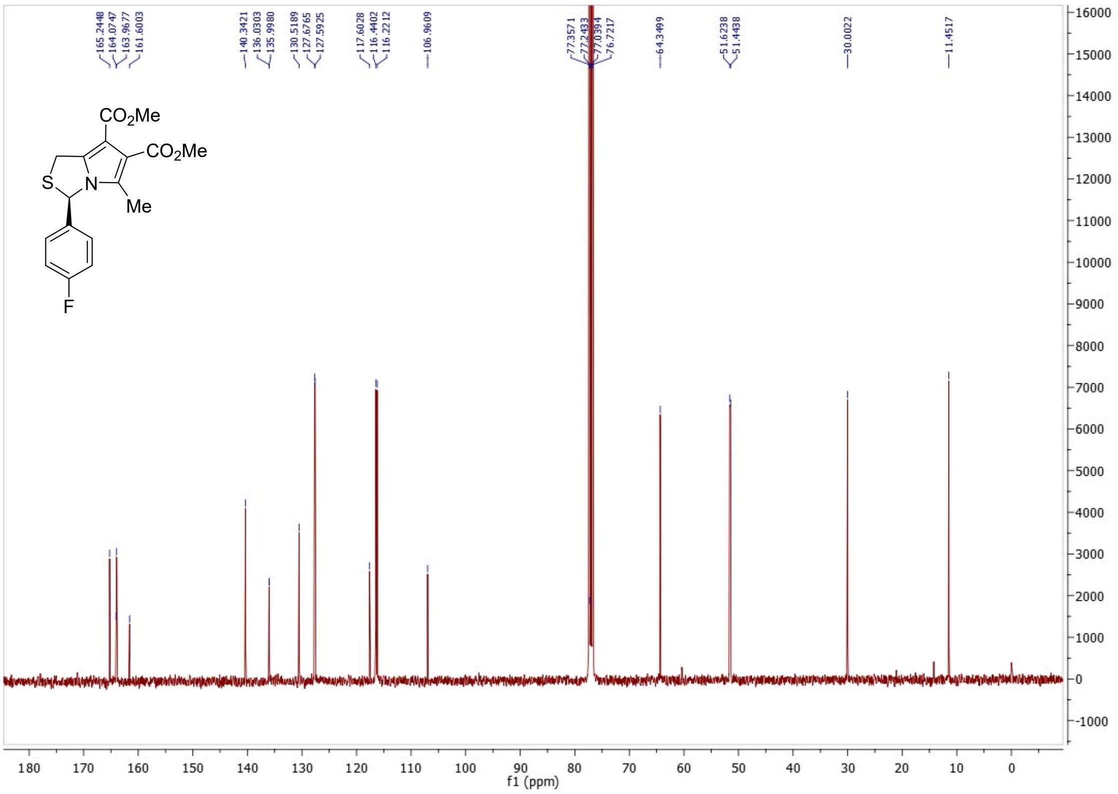

Figure S8: ^{13}C NMR spectrum of compound **3a** (100 MHz, $CDCl_3$).

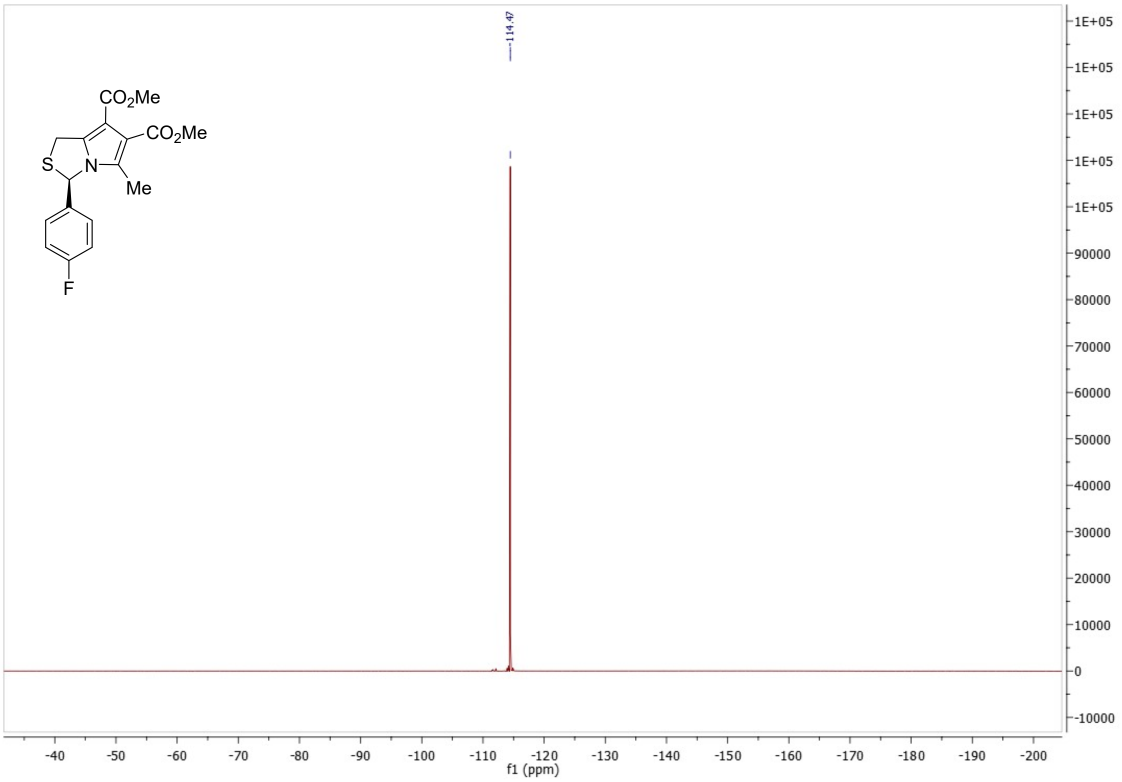

Figure S9: ^{19}F NMR spectrum of compound **3a** (376 MHz, $CDCl_3$).

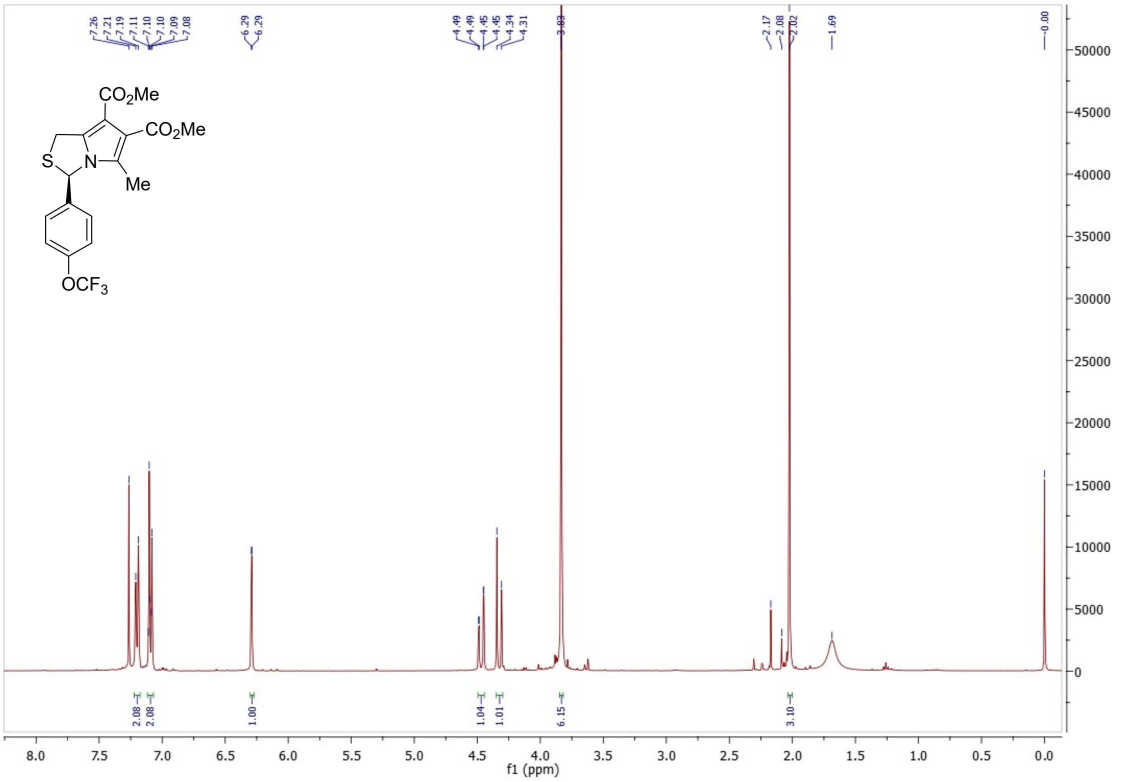

Figure S10: ^{1}H NMR spectrum of compound **3b** (400 MHz, $CDCl_3$).

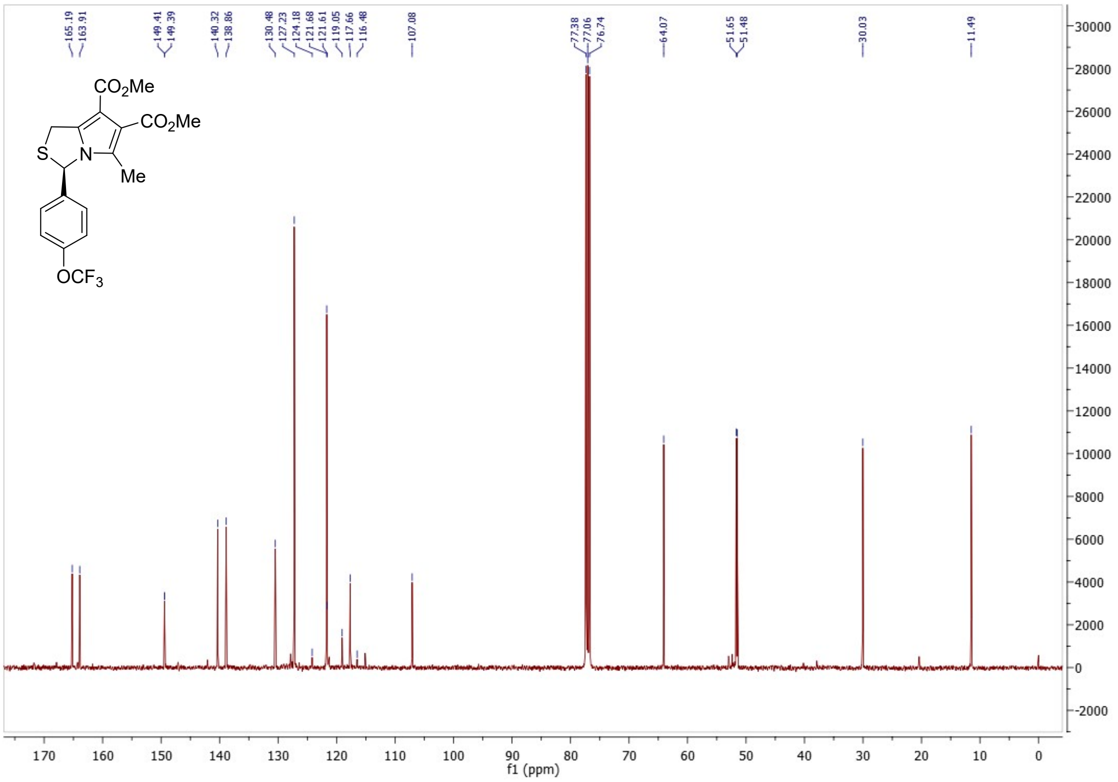

Figure S11: ^{13}C NMR spectrum of compound **3b** (100 MHz, $CDCl_3$).

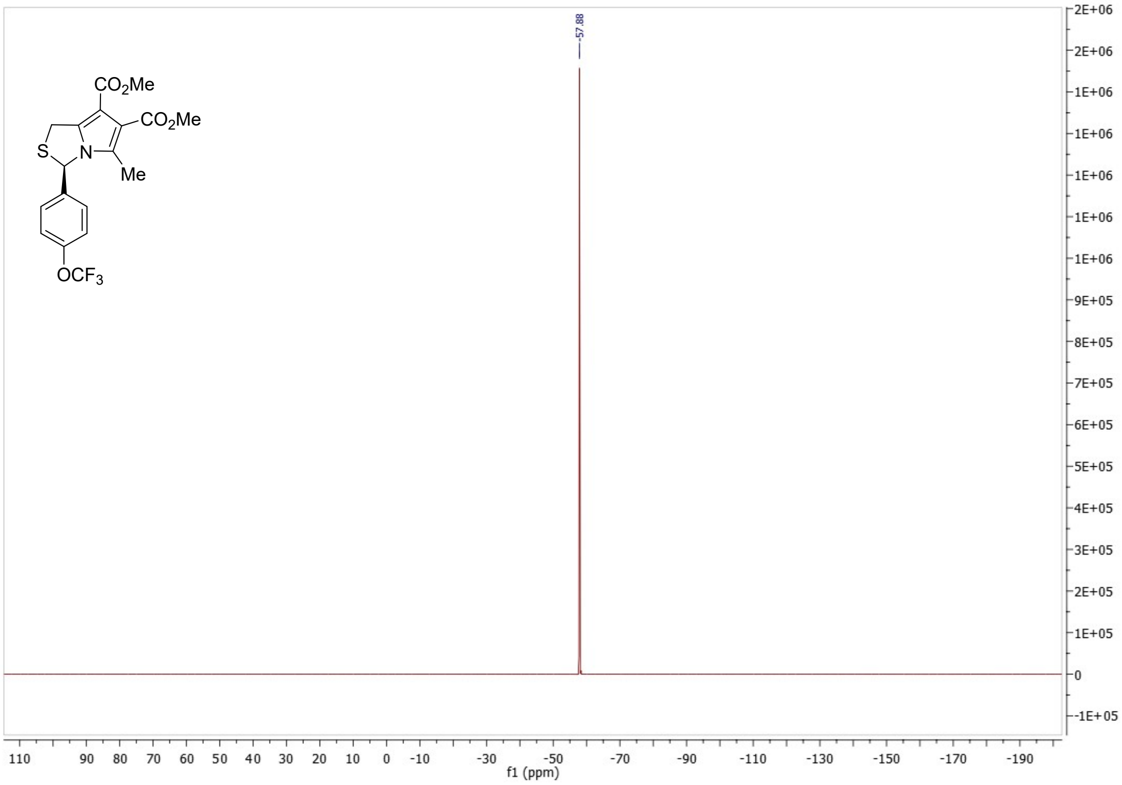

Figure S12: ^{19}F NMR spectrum of compound **3b** (376 MHz, $CDCl_3$).

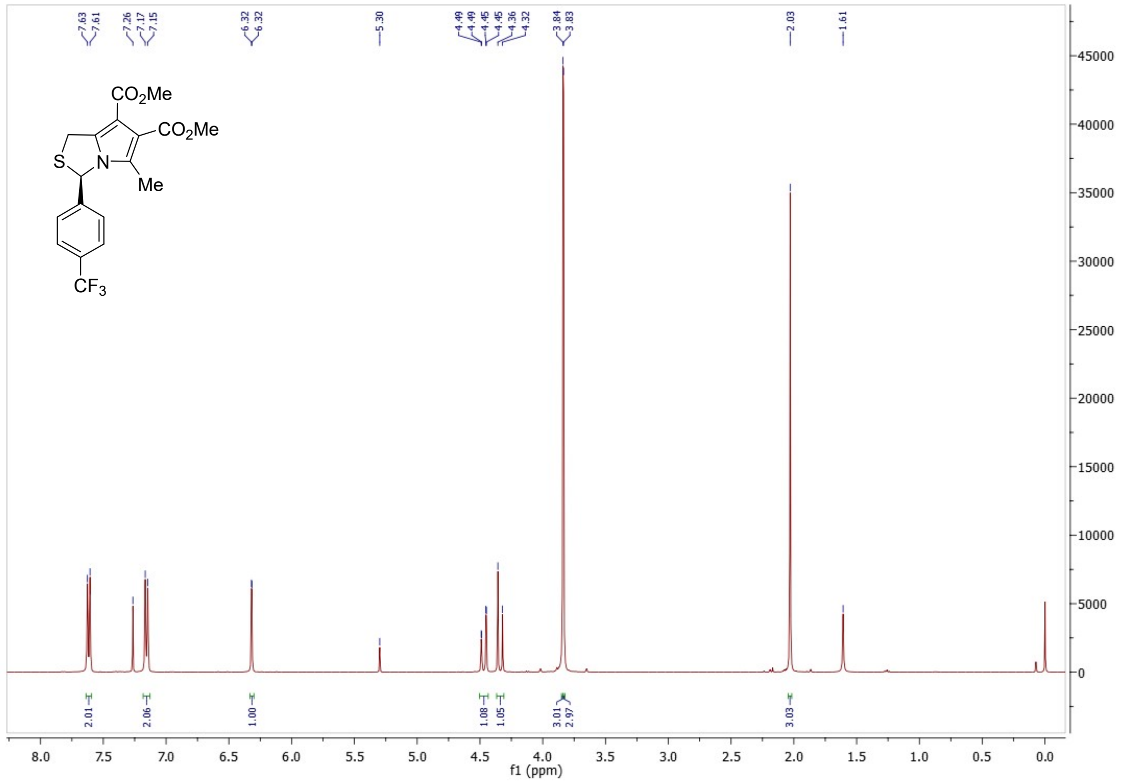

Figure S13: ^{1}H NMR spectrum of compound **3c** (400 MHz, $CDCl_3$).

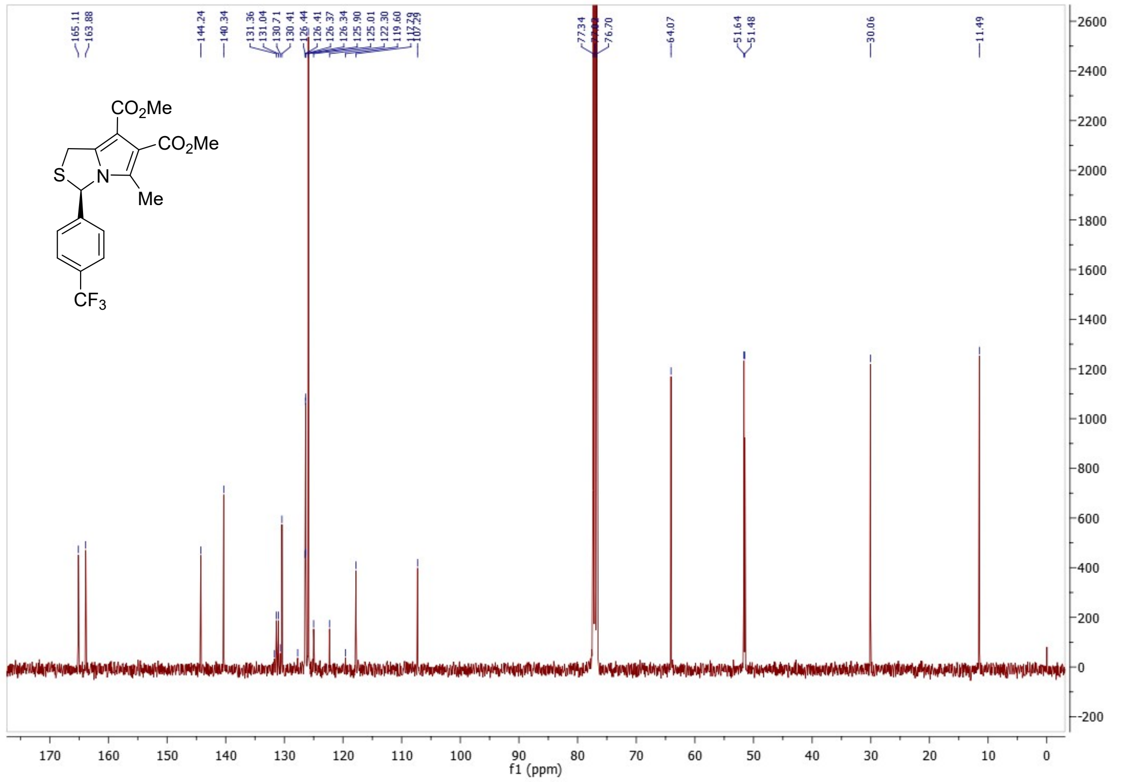

Figure S14: ^{13}C NMR spectrum of compound **3c** (100 MHz, $CDCl_3$).

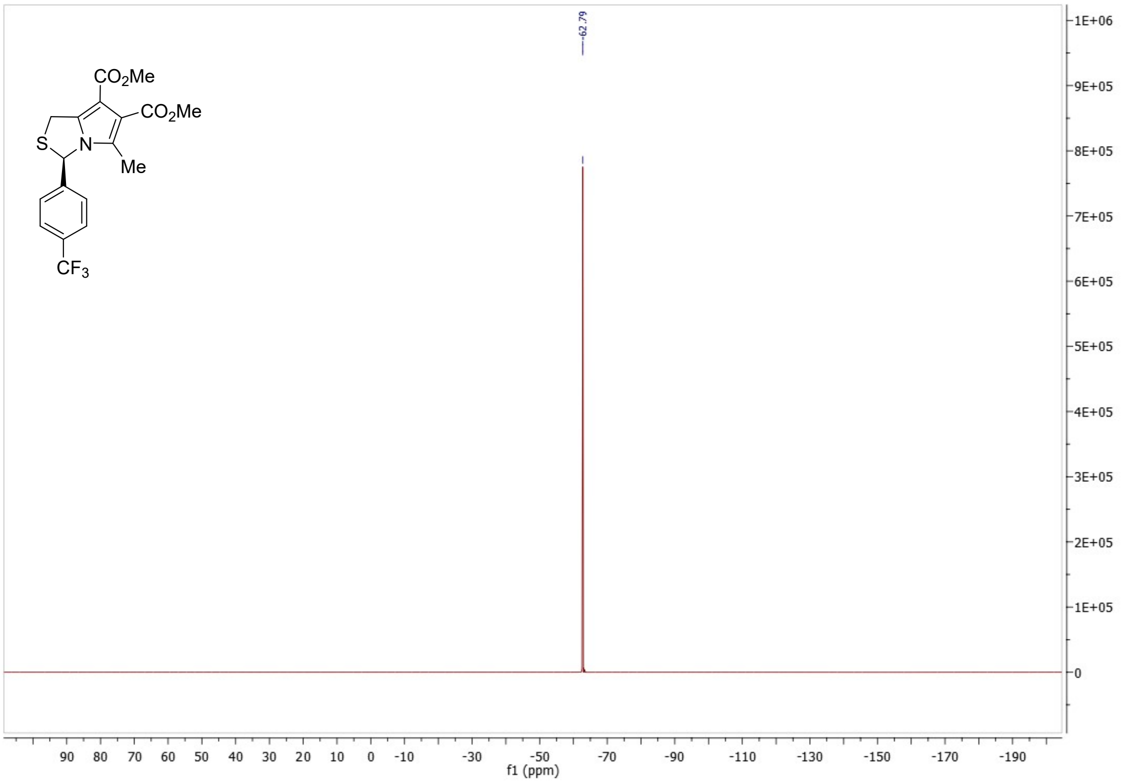

Figure S15: ^{19}F NMR spectrum of compound **3c** (376 MHz, $CDCl_3$).

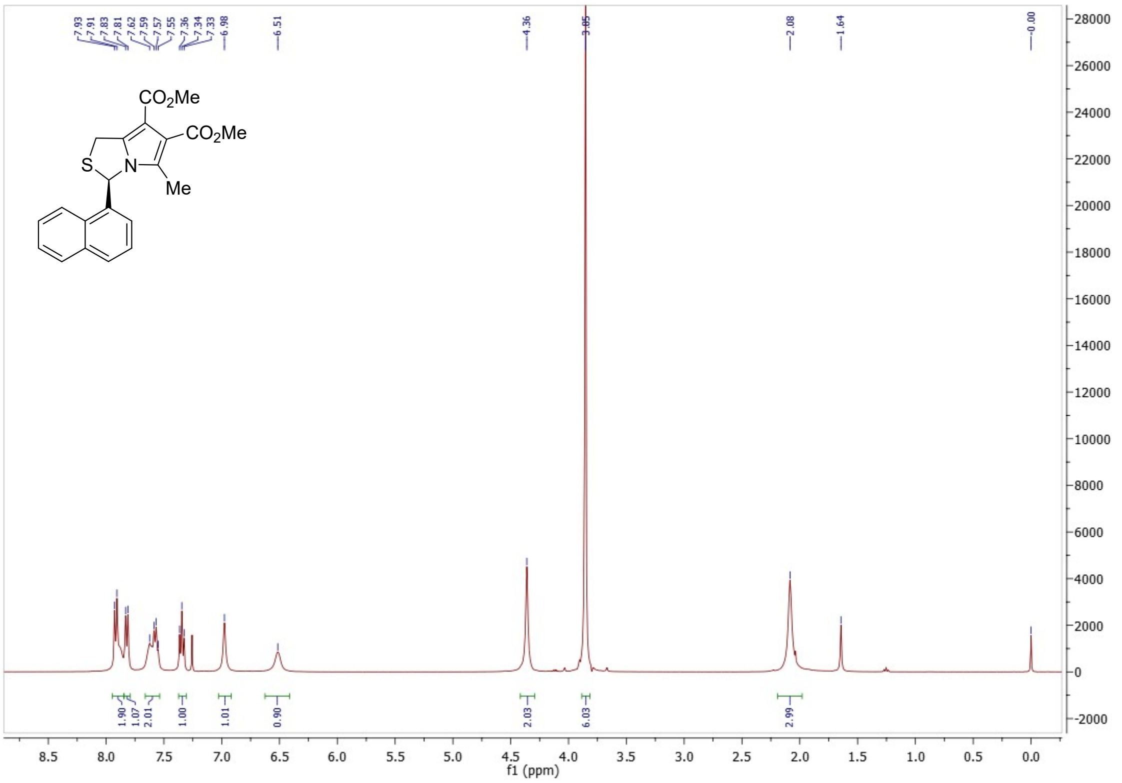

Figure S16: ^{1}H NMR spectrum of compound **3d** (400 MHz, $CDCl_3$).

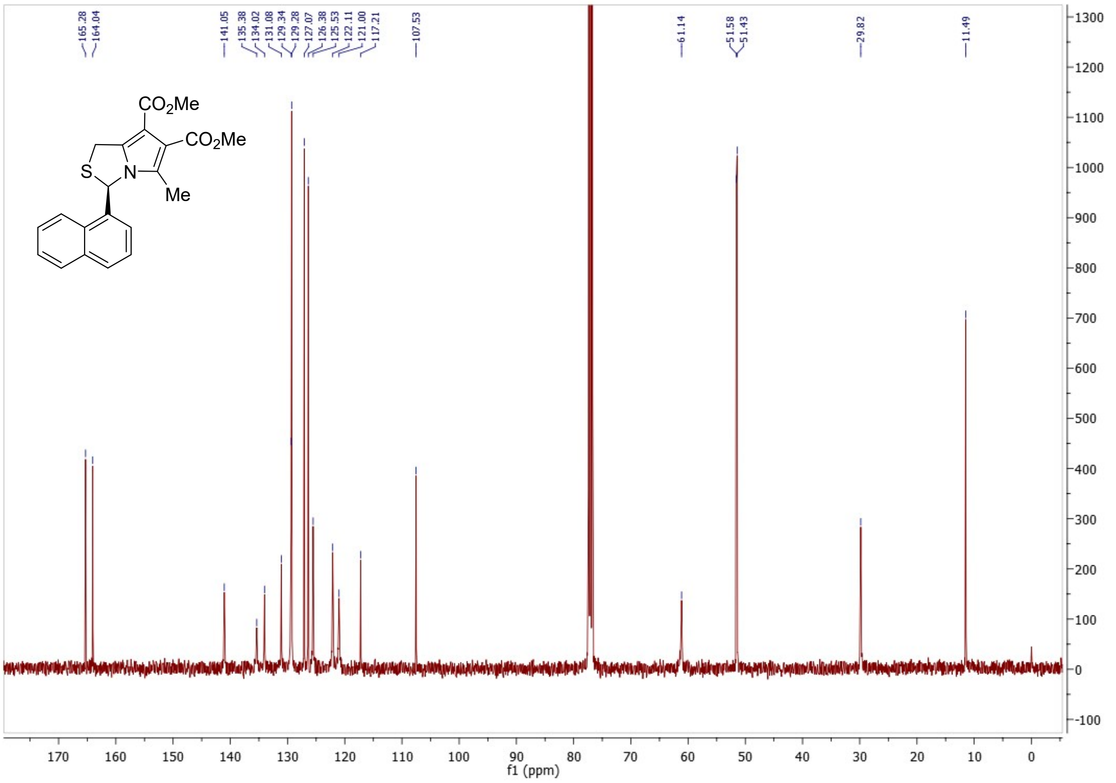

Figure S17: ^{13}C NMR spectrum of compound **3d** (100 MHz, $CDCl_3$).

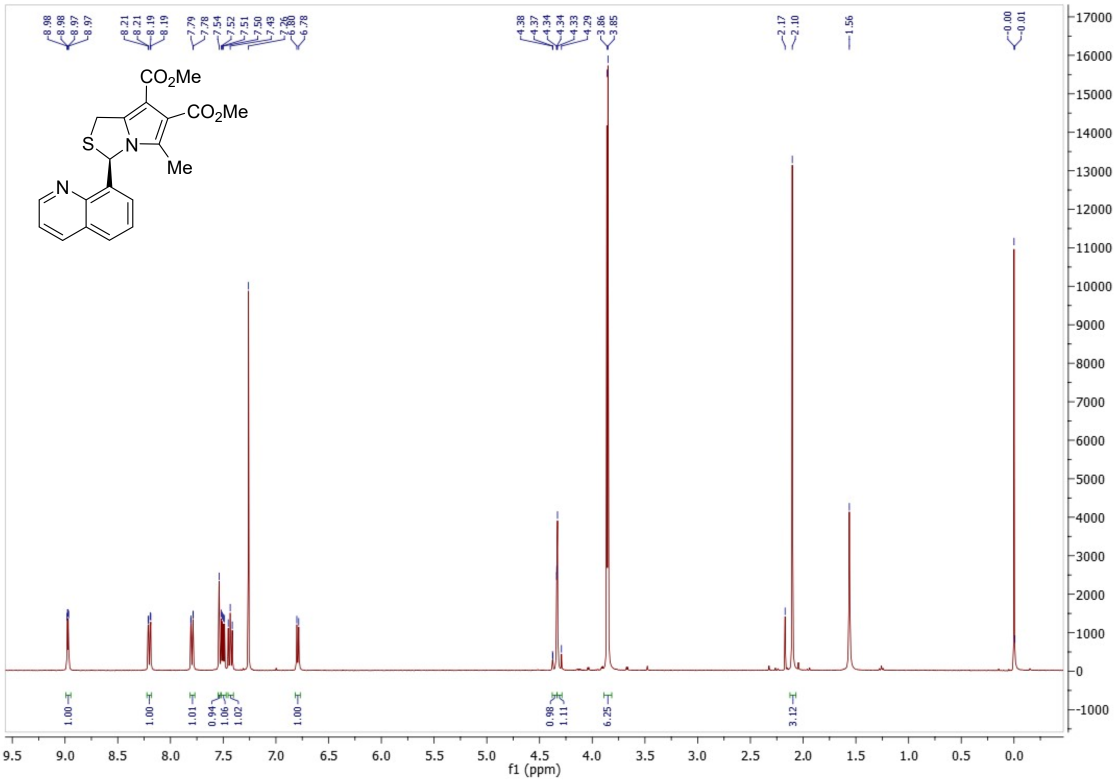

Figure S18: ^{1}H NMR spectrum of compound **3e** (400 MHz, $CDCl_3$).

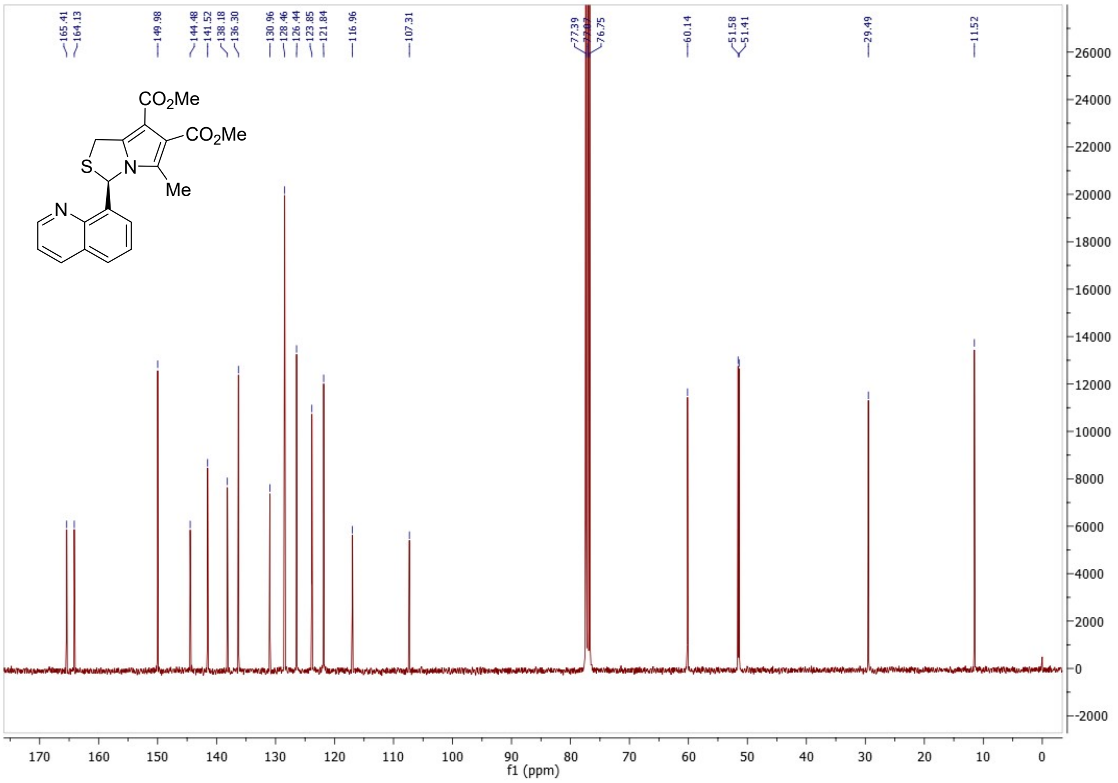

Figure S19: ^{13}C NMR spectrum of compound **3e** (100 MHz, $CDCl_3$).

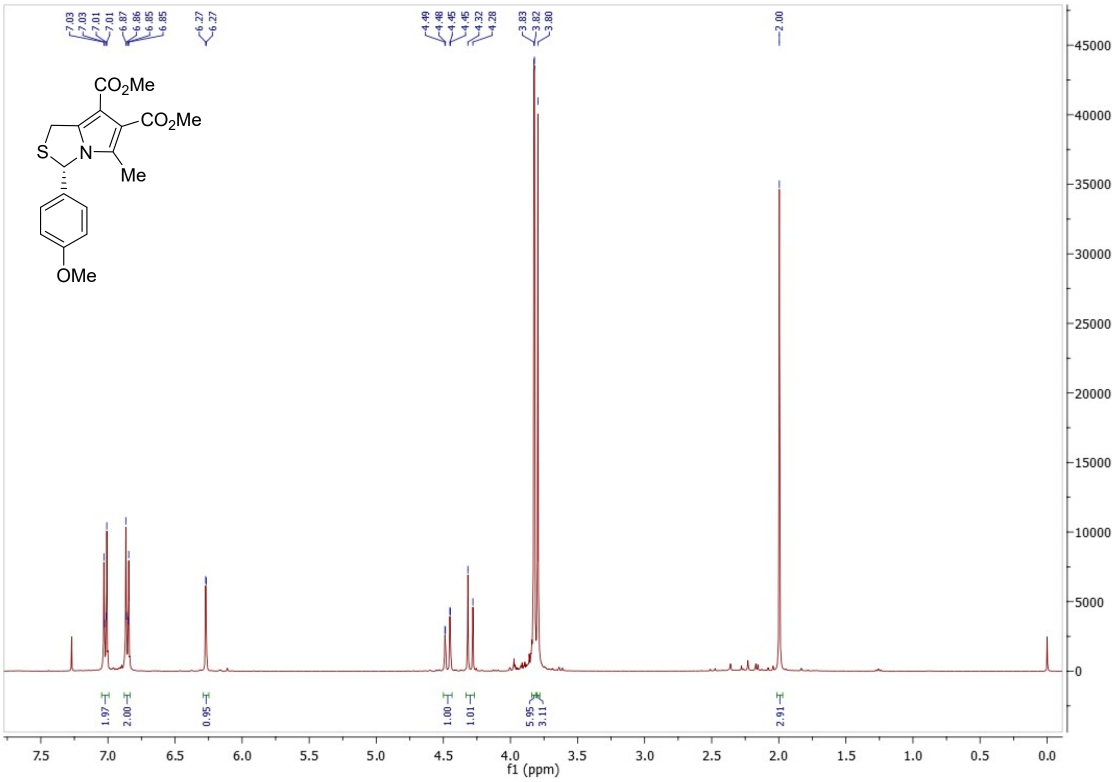

Figure S20: ^{1}H NMR spectrum of compound **6** (400 MHz, $CDCl_3$).

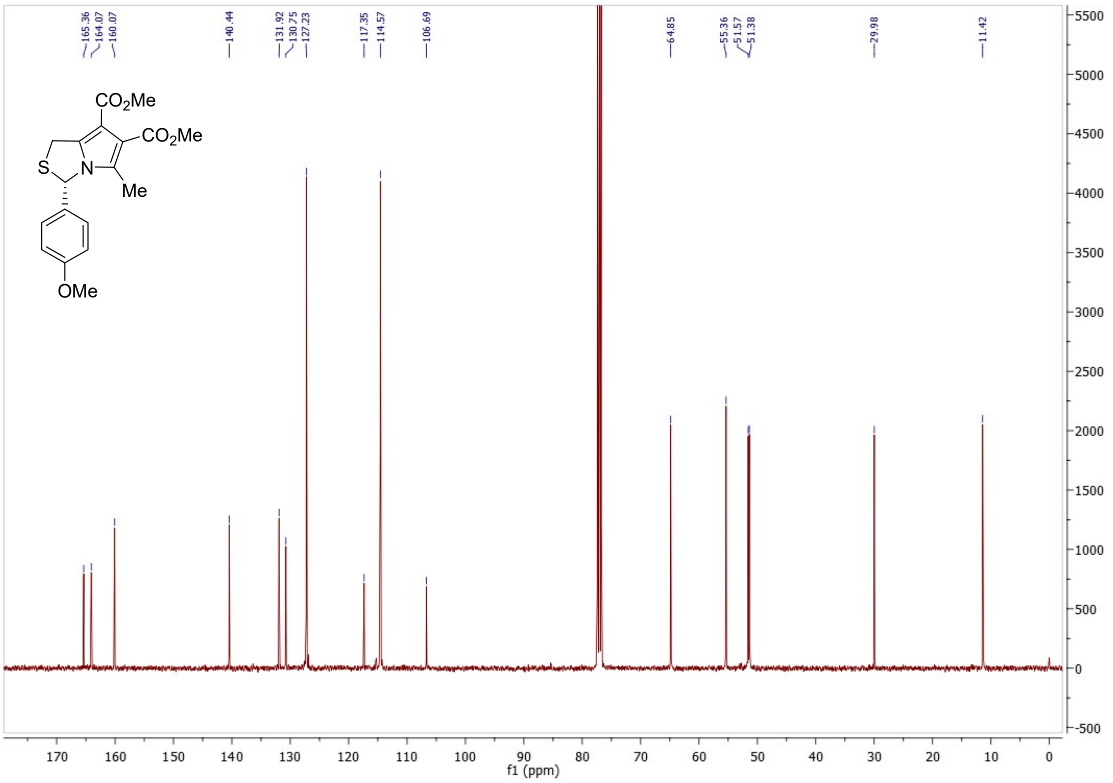

Figure S21: ^{13}C NMR spectrum of compound **6** (100 MHz, $CDCl_3$).

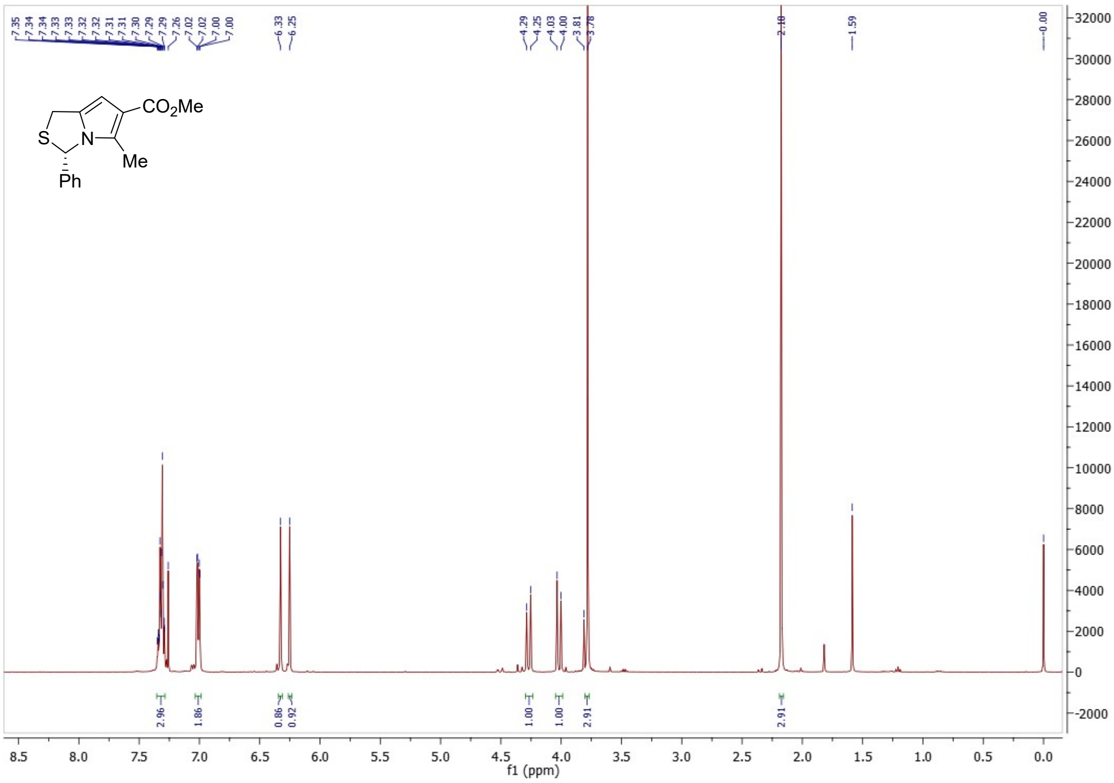

Figure S22: ^{1}H NMR spectrum of compound **10** (400 MHz, $CDCl_3$).

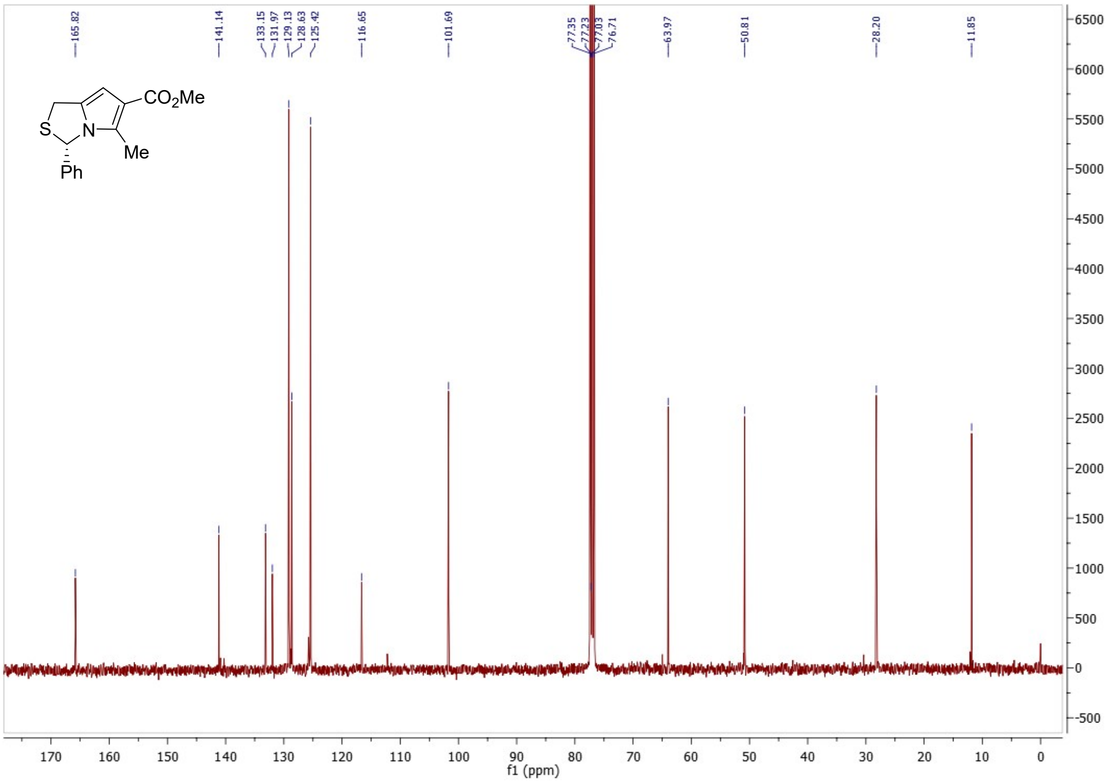

Figure S23: ^{13}C NMR spectrum of compound **10** (100 MHz, $CDCl_3$).

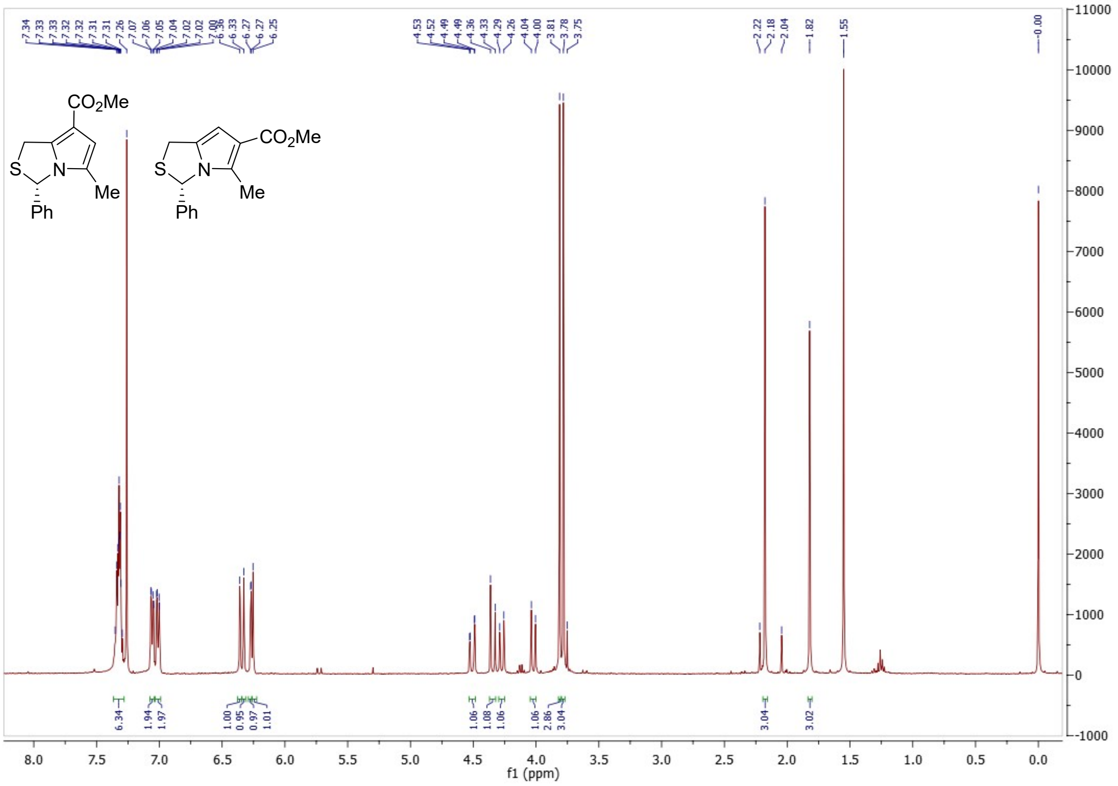

Figure S24: ^{1}H NMR spectrum of the mixture of compounds **9** and **10** (400 MHz, $CDCl_3$).

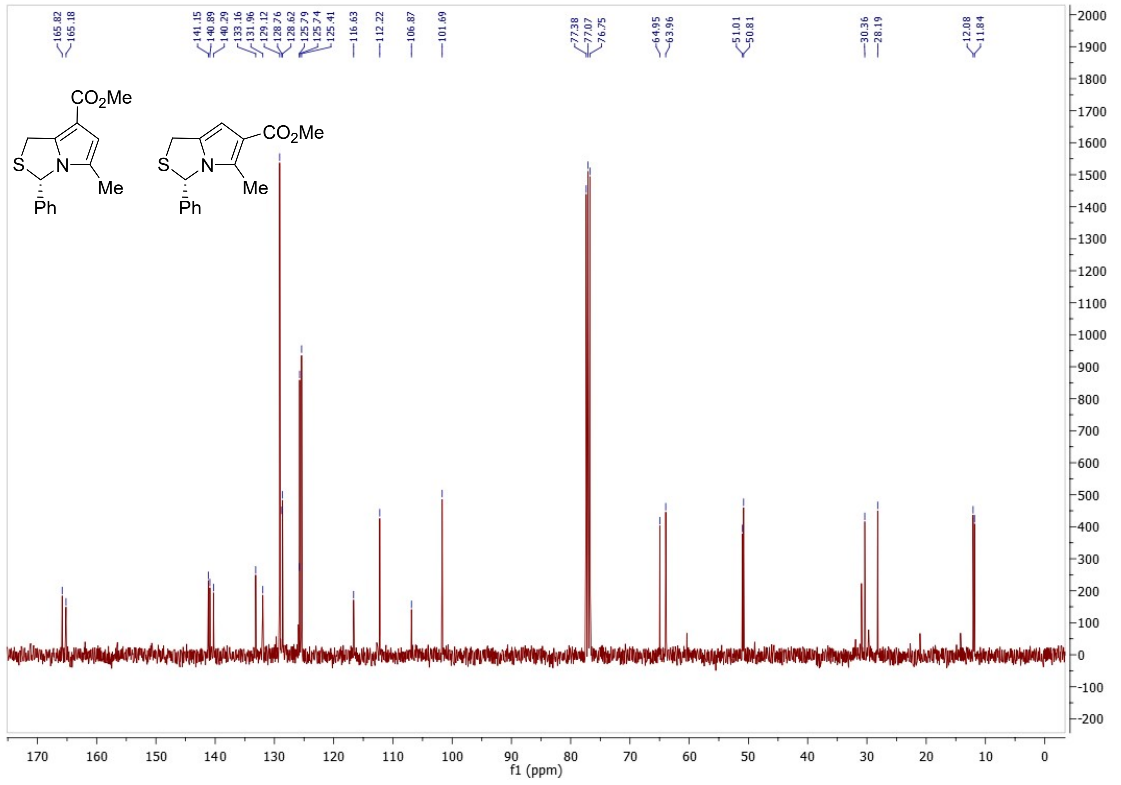

Figure S25: ^{13}C NMR spectrum of the mixture of compounds **9** and **10** (100 MHz, $CDCl_3$).

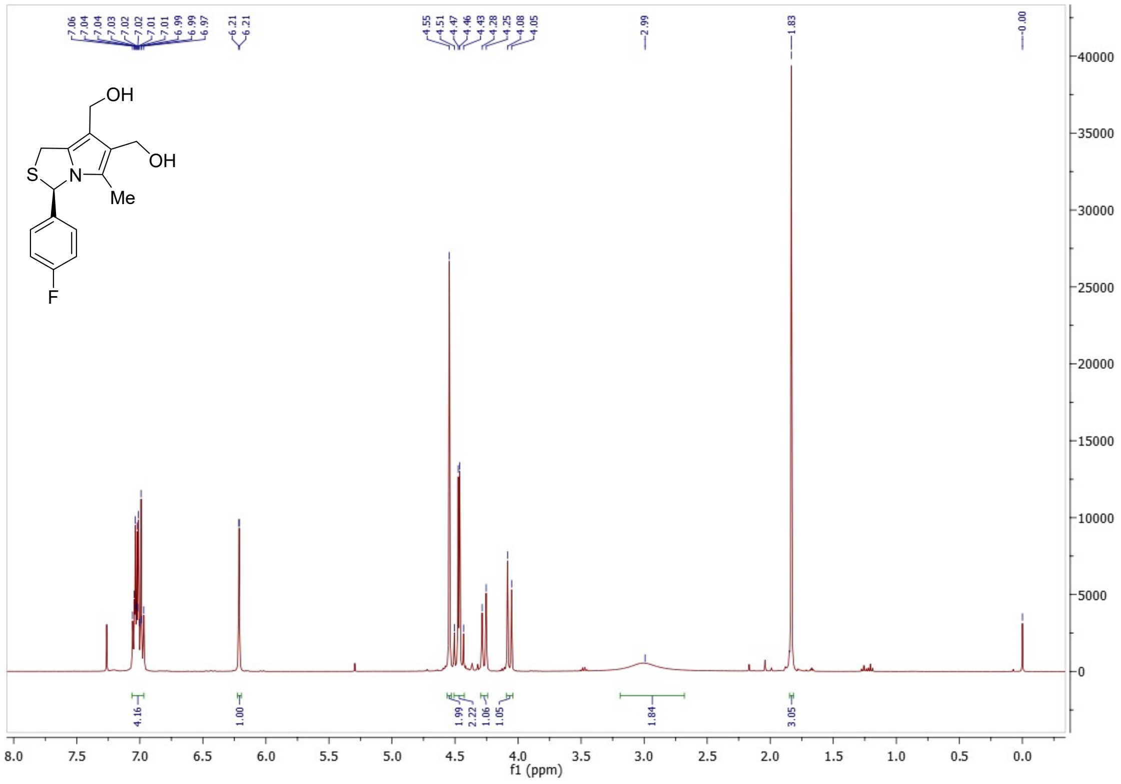

Figure S26: ^{1}H NMR spectrum of compound **4a** (400 MHz, $CDCl_3$).

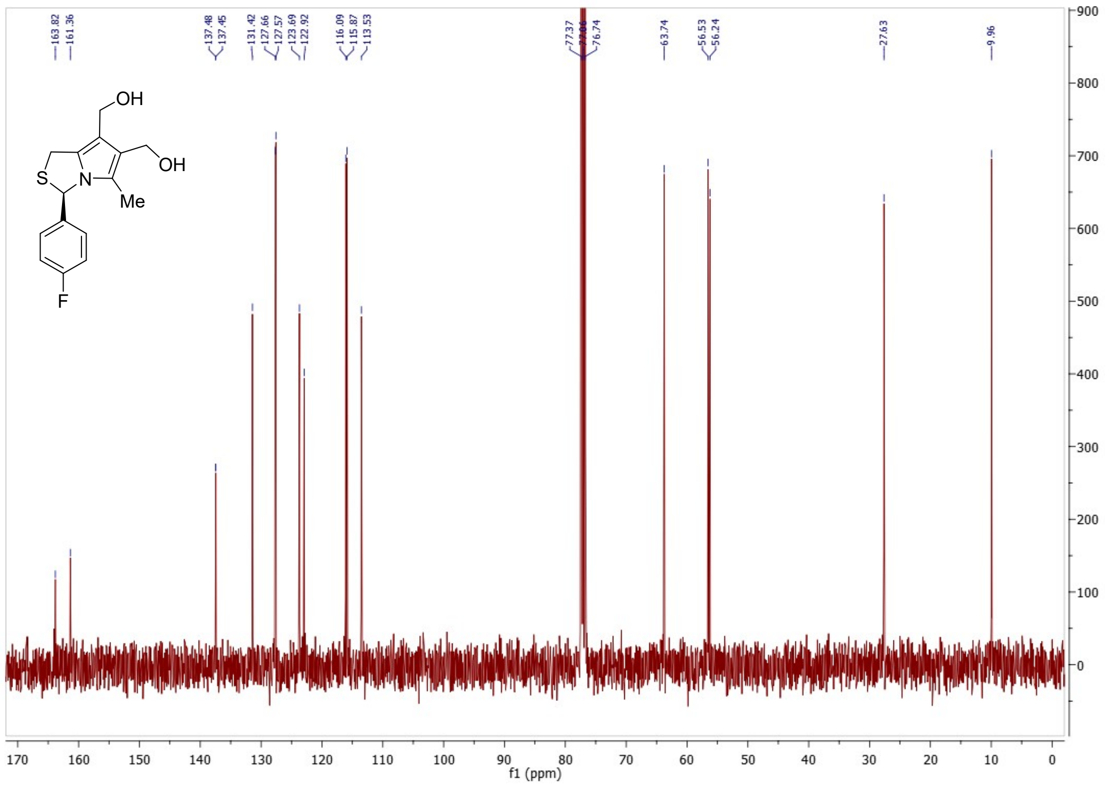

Figure S27: ^{13}C NMR spectrum of compound **4a** (100 MHz, $CDCl_3$).

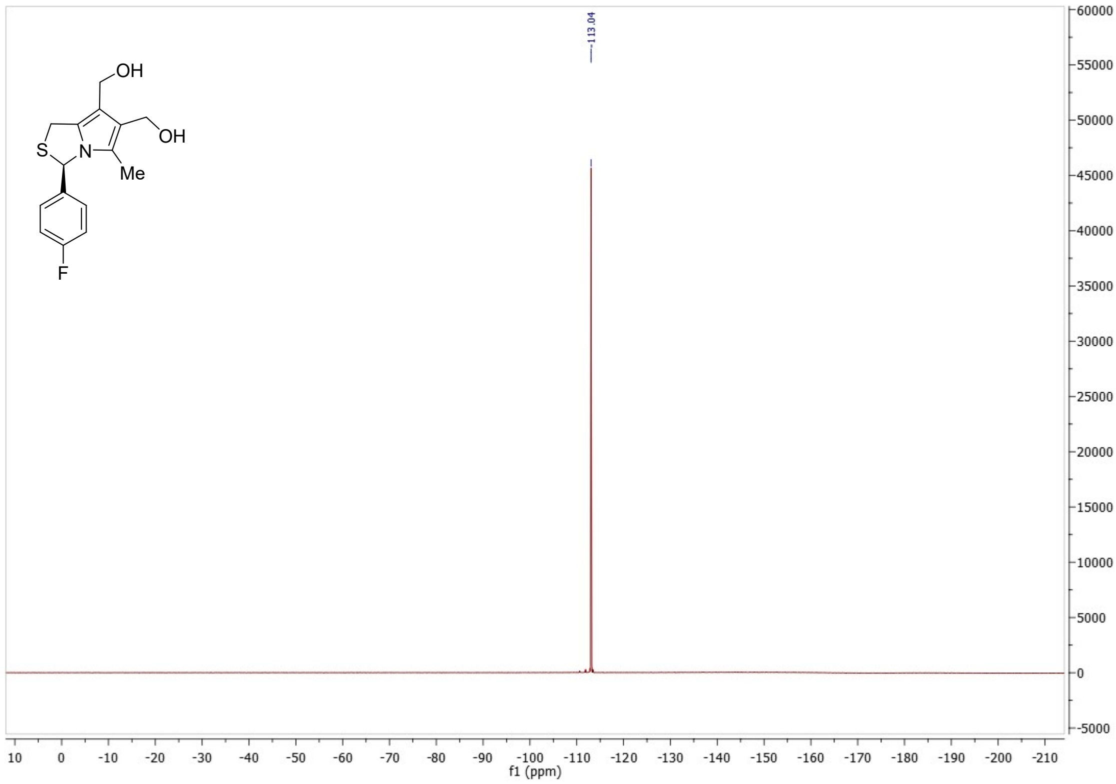

Figure S28: ^{19}F NMR spectrum of compound **4a** (376 MHz, $CDCl_3$).

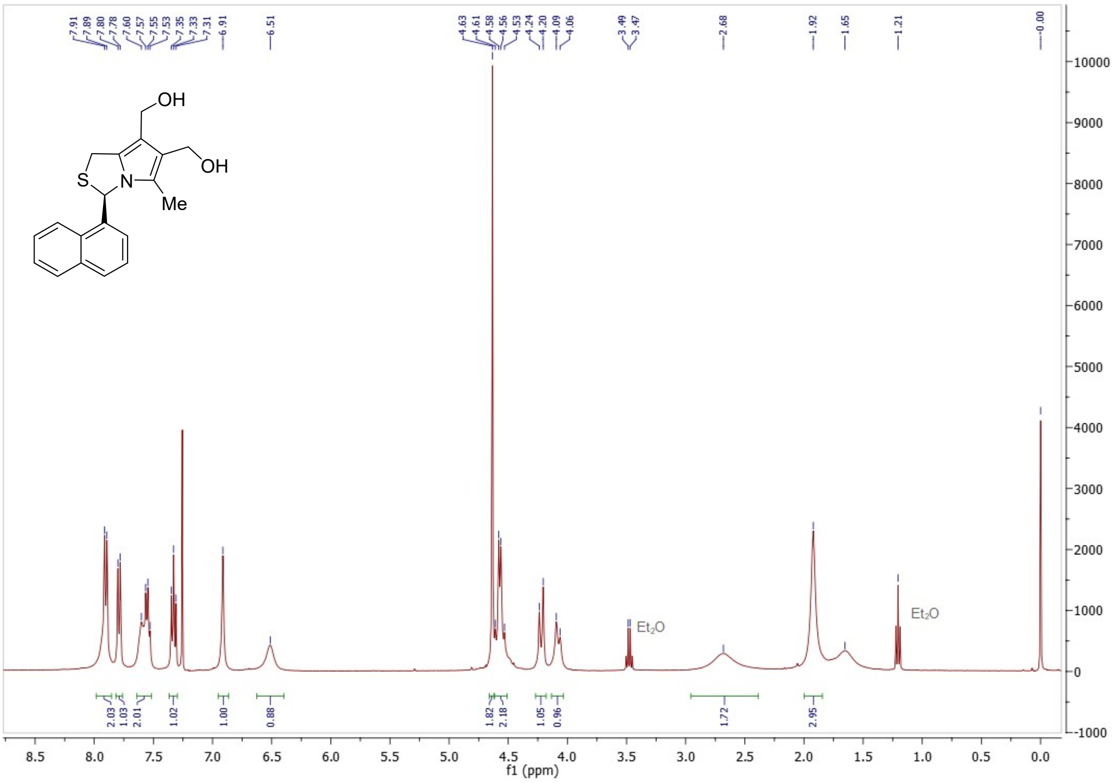

Figure S29: ^{1}H NMR spectrum of compound **4d** (400 MHz, $CDCl_3$).

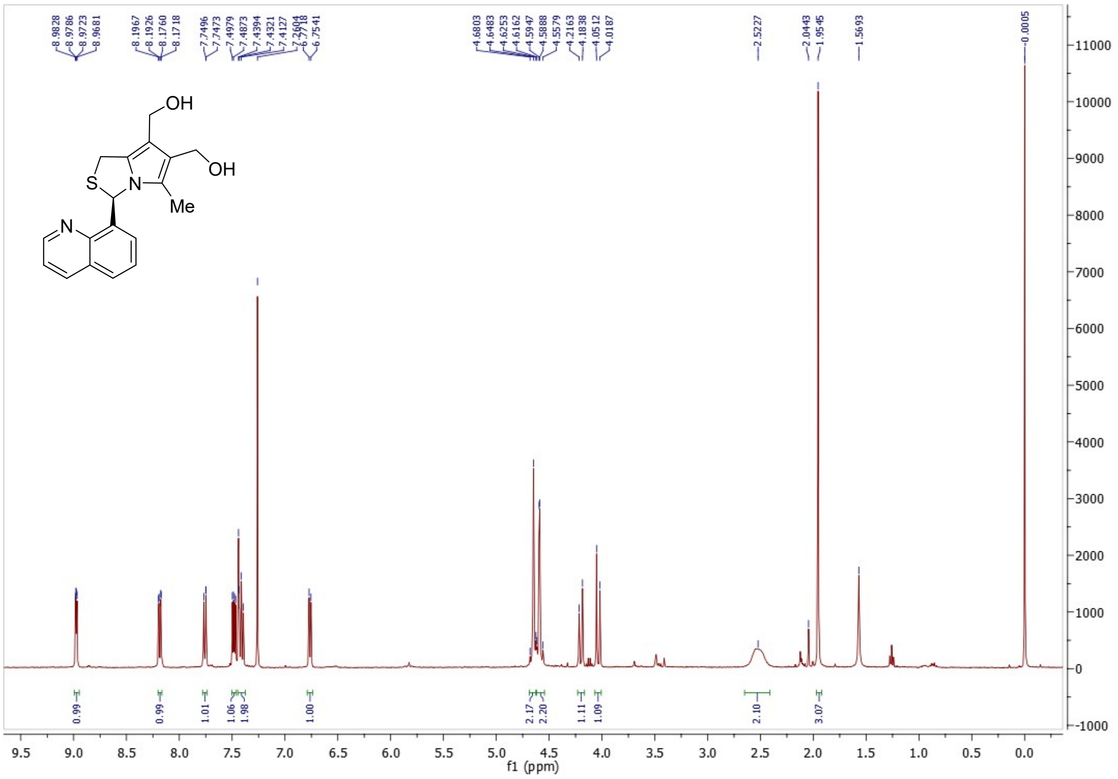

Figure S30: ^{1}H NMR spectrum of compound **4e** (400 MHz, $CDCl_3$).

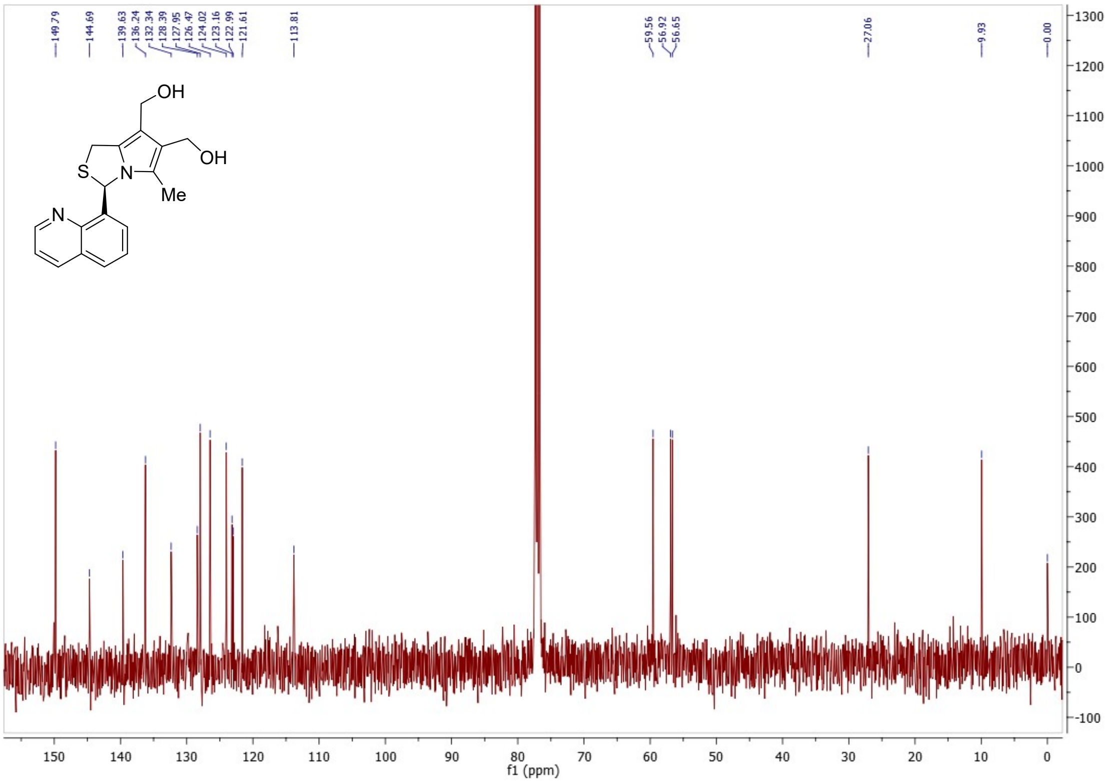

Figure S31: ^{13}C NMR spectrum of compound **4e** (100 MHz, $CDCl_3$).

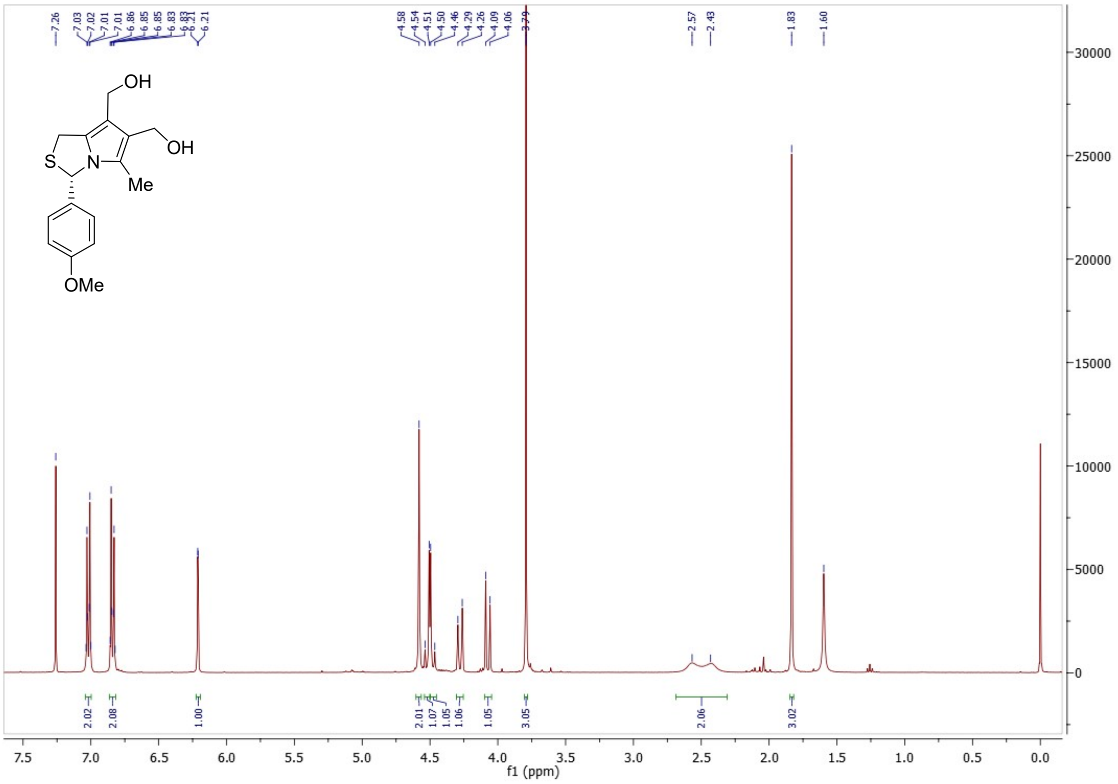

Figure S32: ^{1}H NMR spectrum of compound **7** (400 MHz, $CDCl_3$).

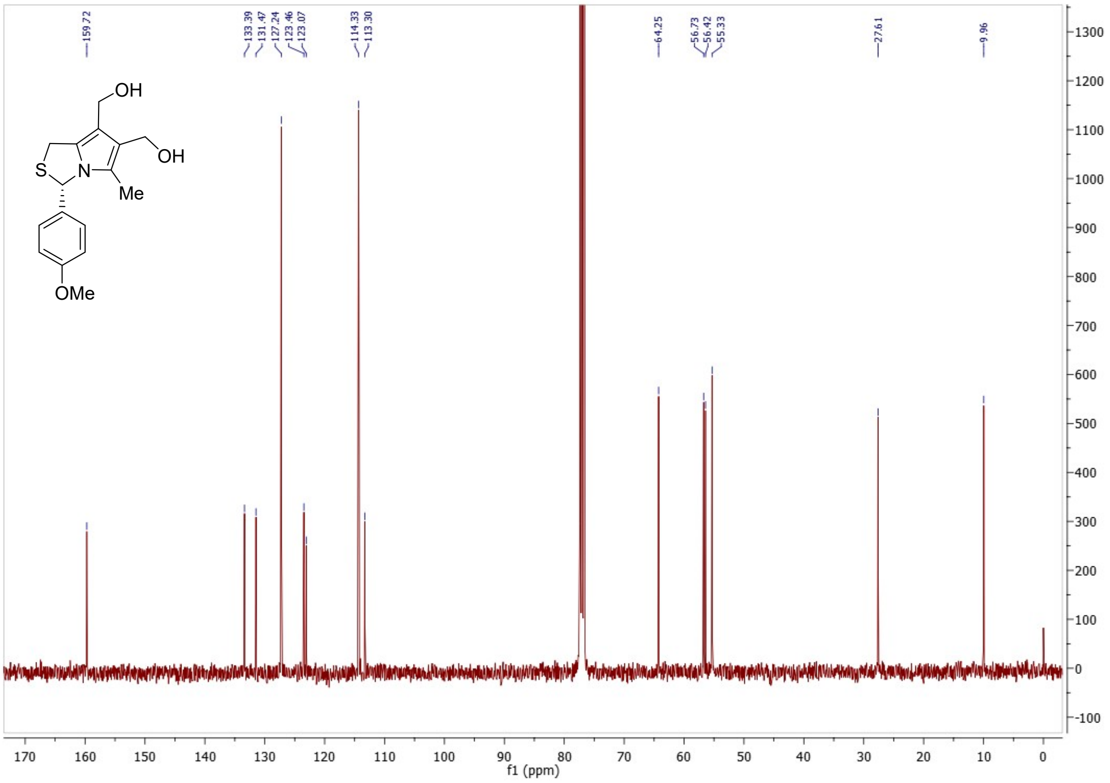

Figure S33: ^{13}C NMR spectrum of compound **7** (100 MHz, $CDCl_3$).

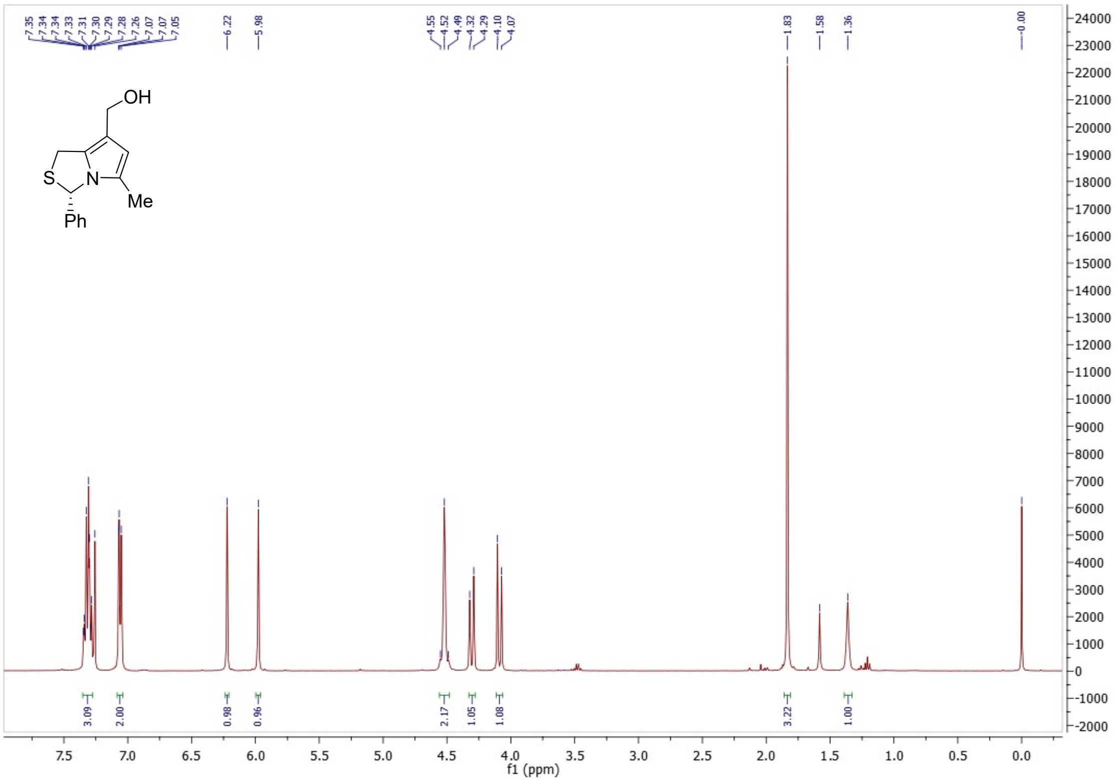

Figure S34: ^{1}H NMR spectrum of compound **11** (400 MHz, $CDCl_3$).

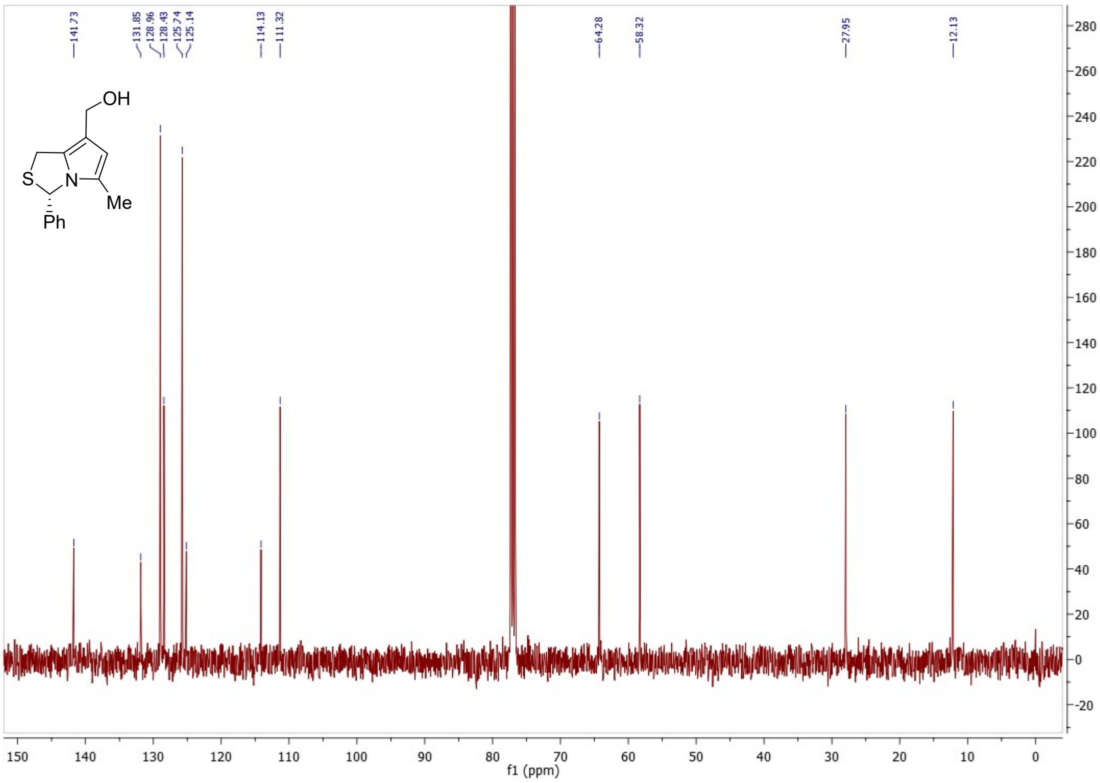

Figure S35: ^{13}C NMR spectrum of compound **11** (100 MHz, $CDCl_3$).

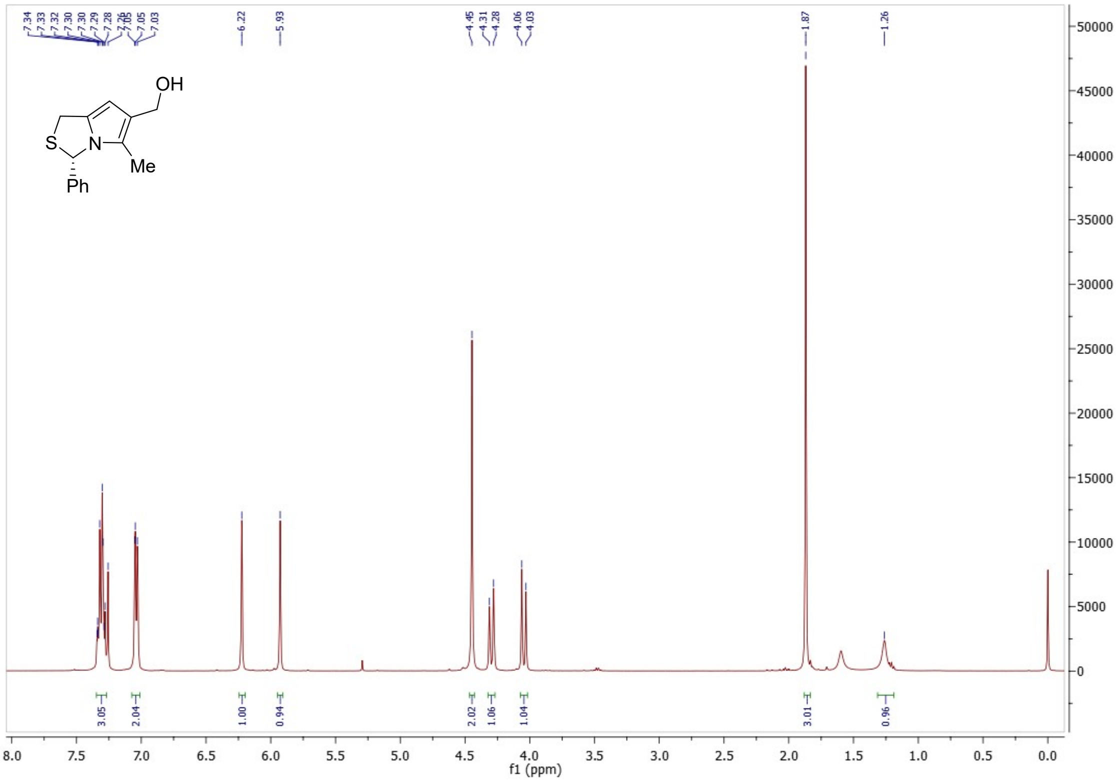

Figure S36: ^{1}H NMR spectrum of compound **12** (400 MHz, $CDCl_3$).

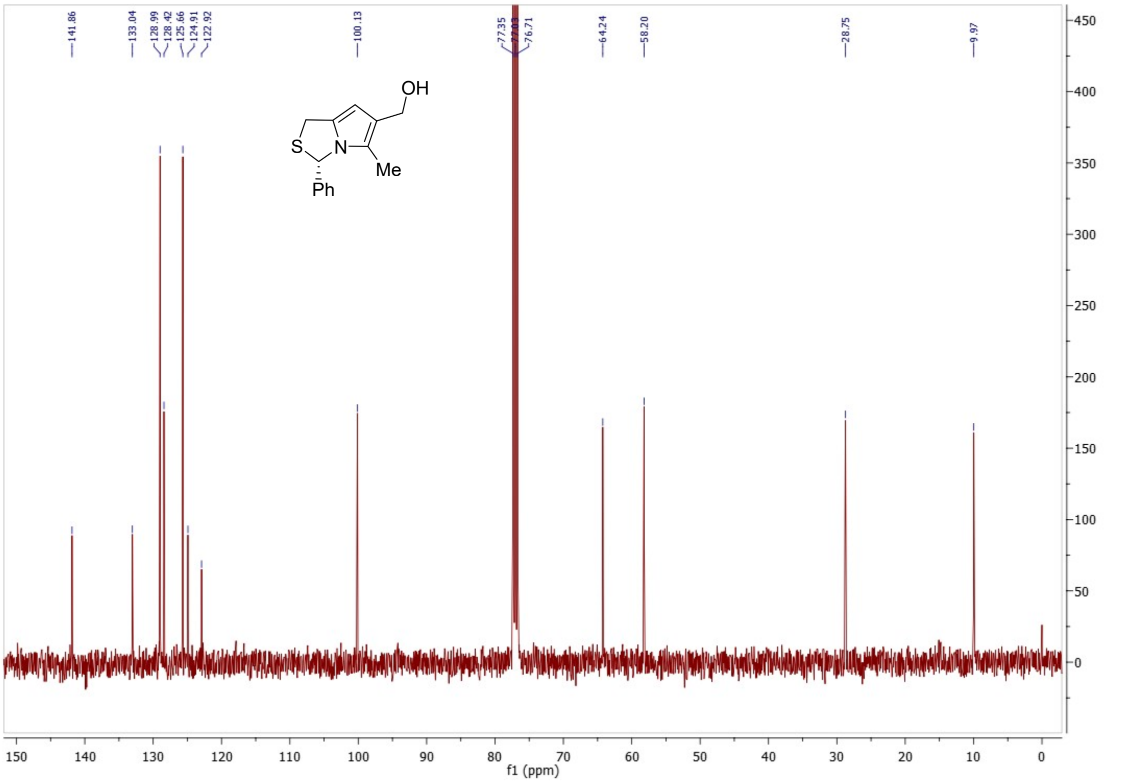

Figure S37: ^{13}C NMR spectrum of compound **12** (100 MHz, $CDCl_3$).

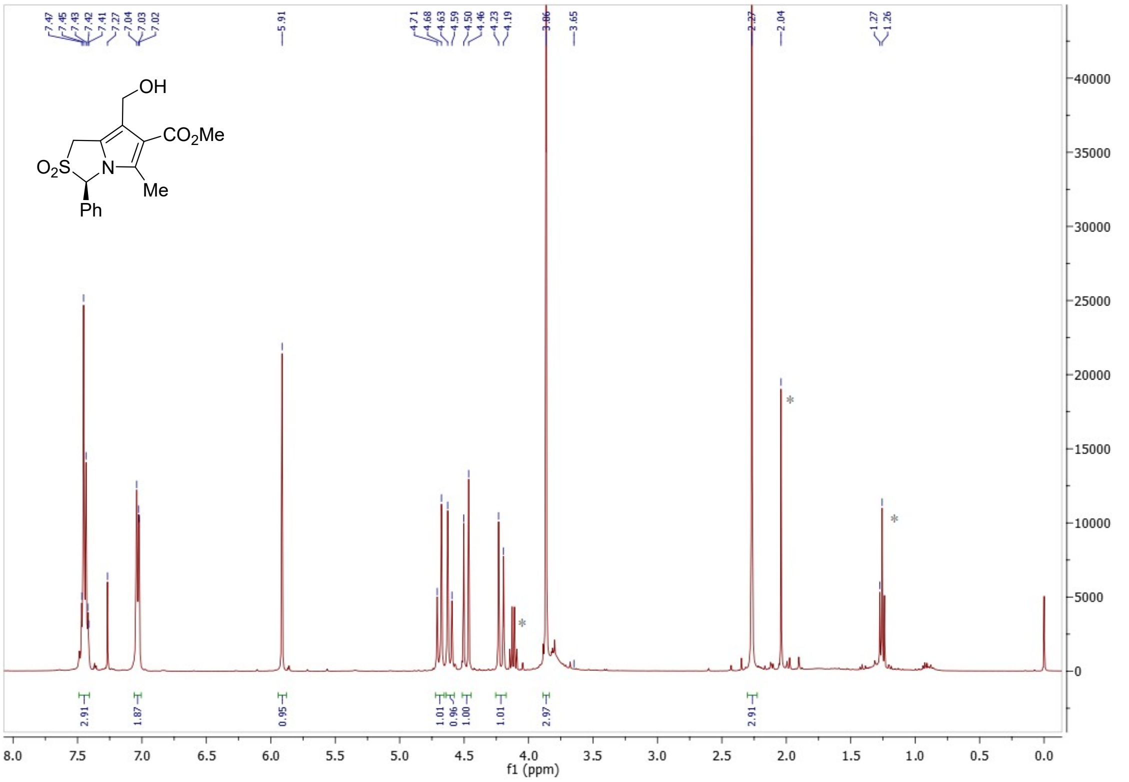

Figure S38: ^{1}H NMR spectrum of compound **14** (400 MHz, $CDCl_3$).*ethyl acetate

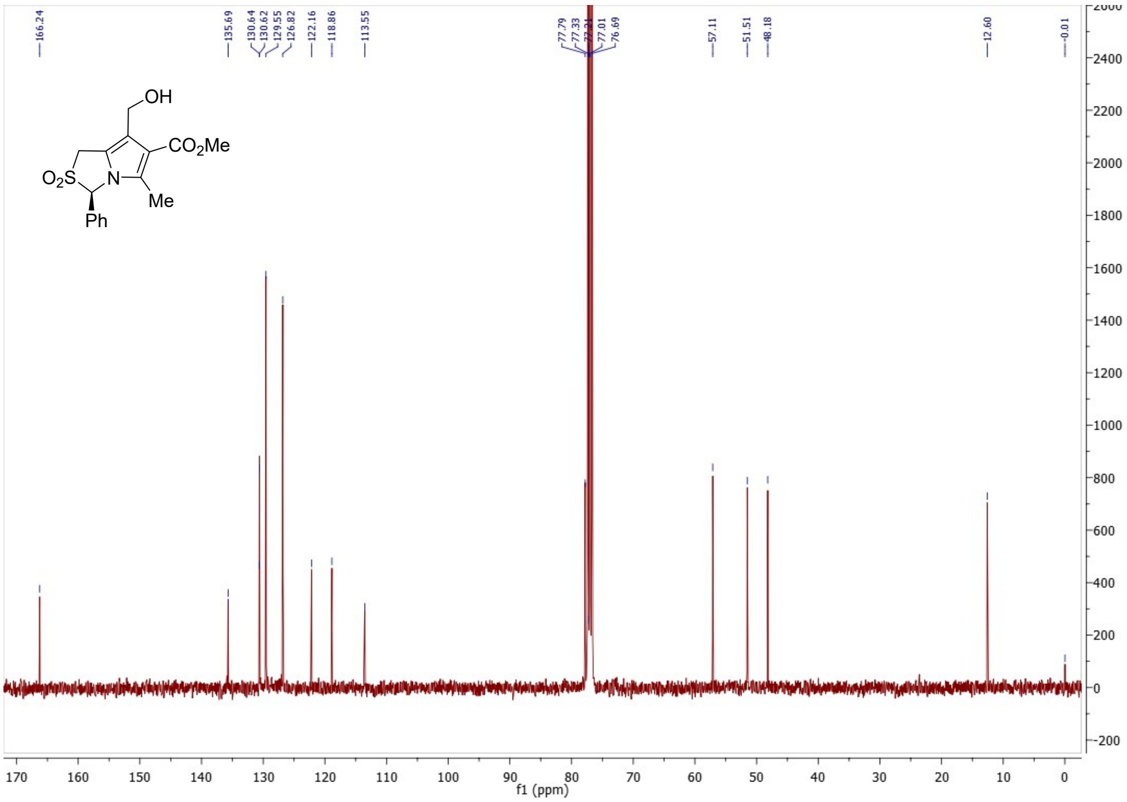

Figure S39: ^{13}C NMR spectrum of compound **14** (100 MHz, $CDCl_3$).

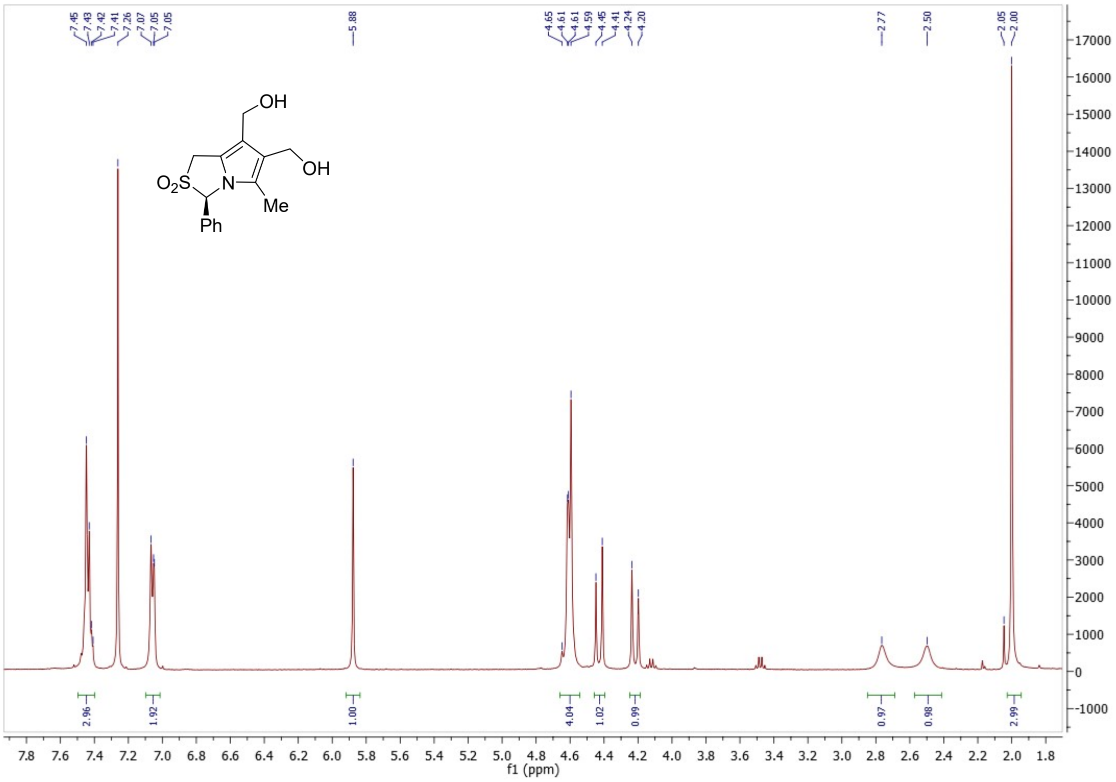

Figure S40: ^{1}H NMR spectrum of compound **15** (400 MHz, $CDCl_3$).

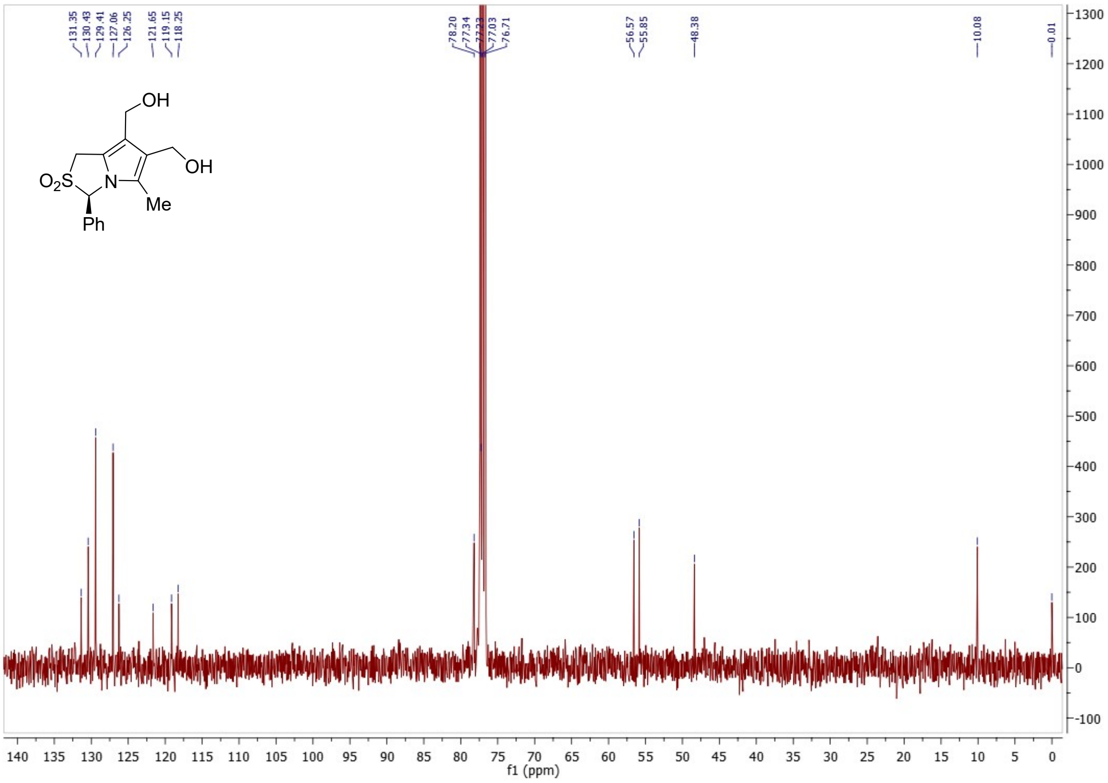

Figure S41: ^{13}C NMR spectrum of compound **15** (100 MHz, $CDCl_3$).

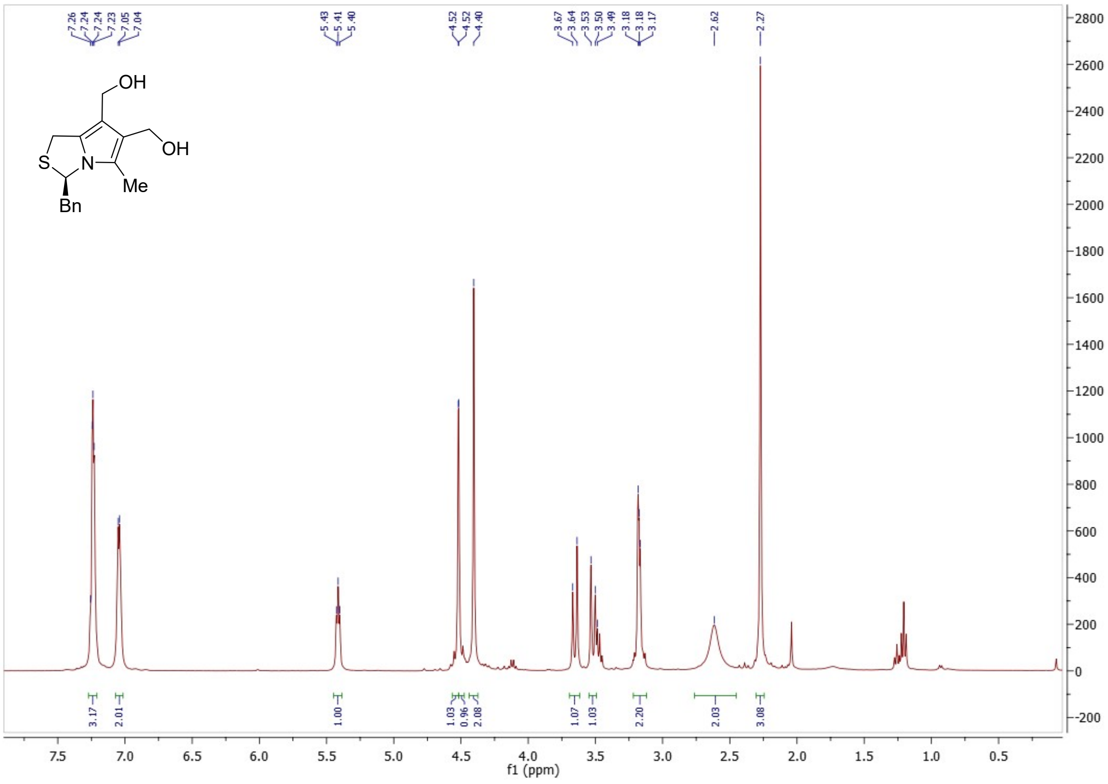

Figure S42: ^{1}H NMR spectrum of compound **17a** (400 MHz, $CDCl_3$).

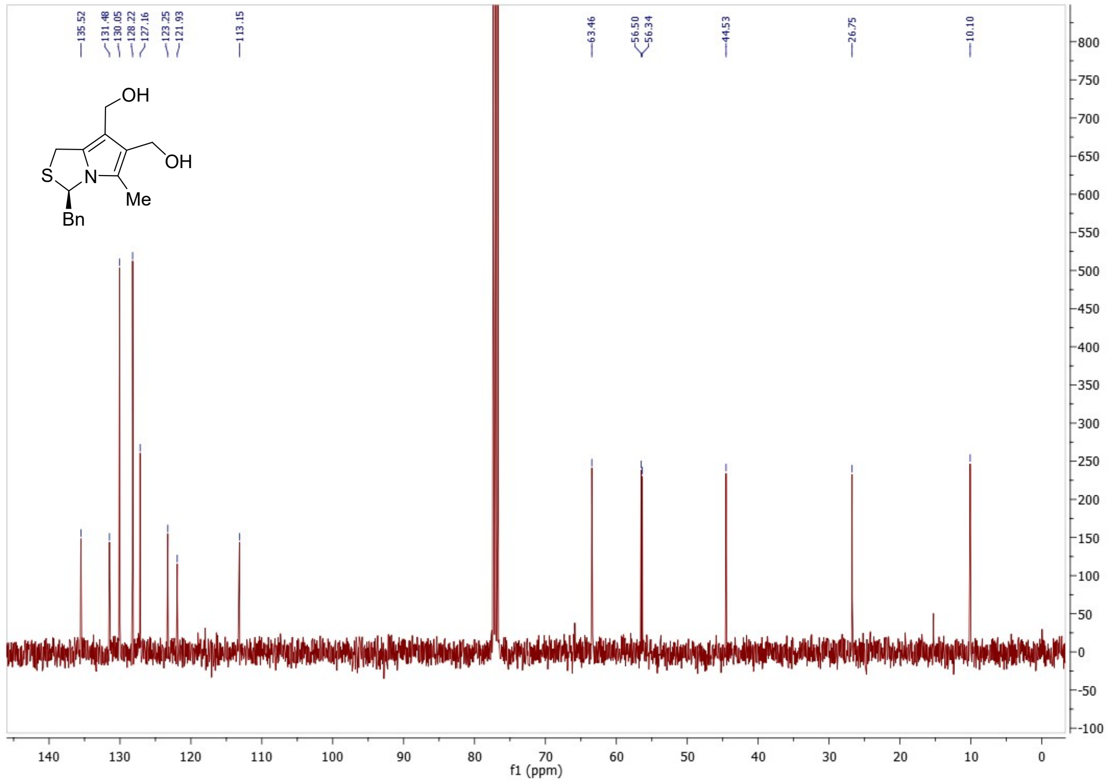

Figure S43: ^{13}C NMR spectrum of compound **17a** (100 MHz, $CDCl_3$).

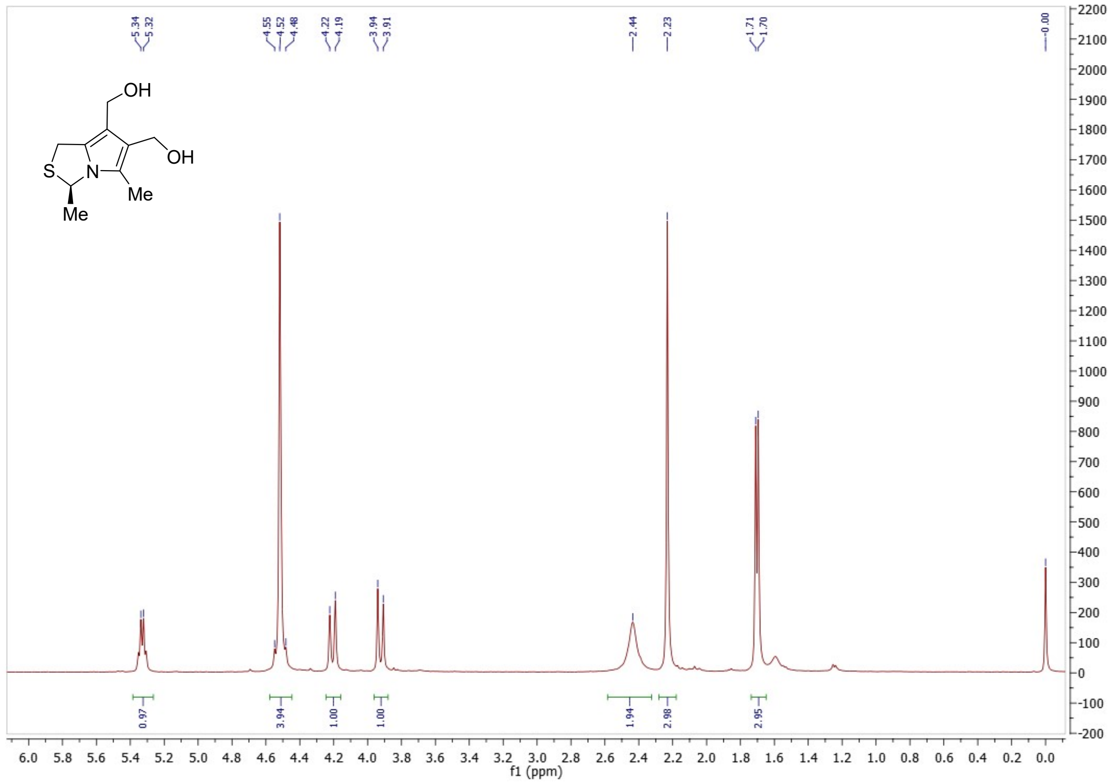

Figure S44: ^{1}H NMR spectrum of compound **17b** (400 MHz, $CDCl_3$).

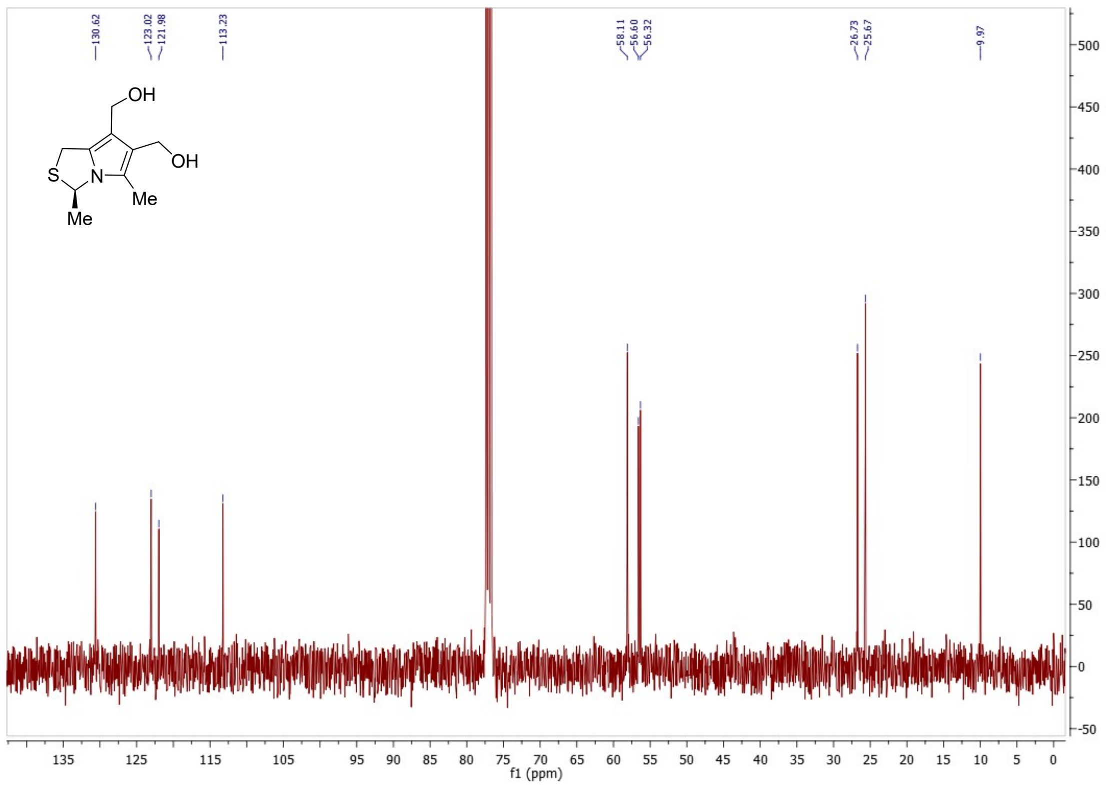

Figure S45: ^{13}C NMR spectrum of compound **17b** (100 MHz, $CDCl_3$).

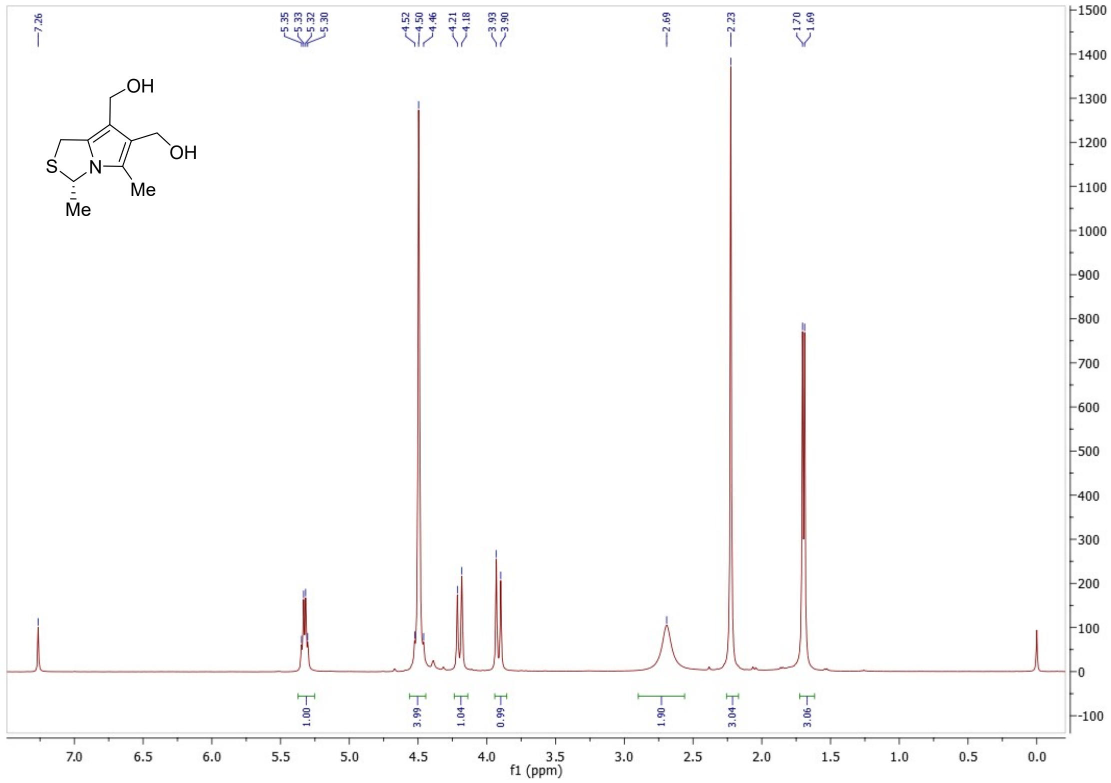

Figure S46: ^{1}H NMR spectrum of compound **17c** (400 MHz, $CDCl_3$).

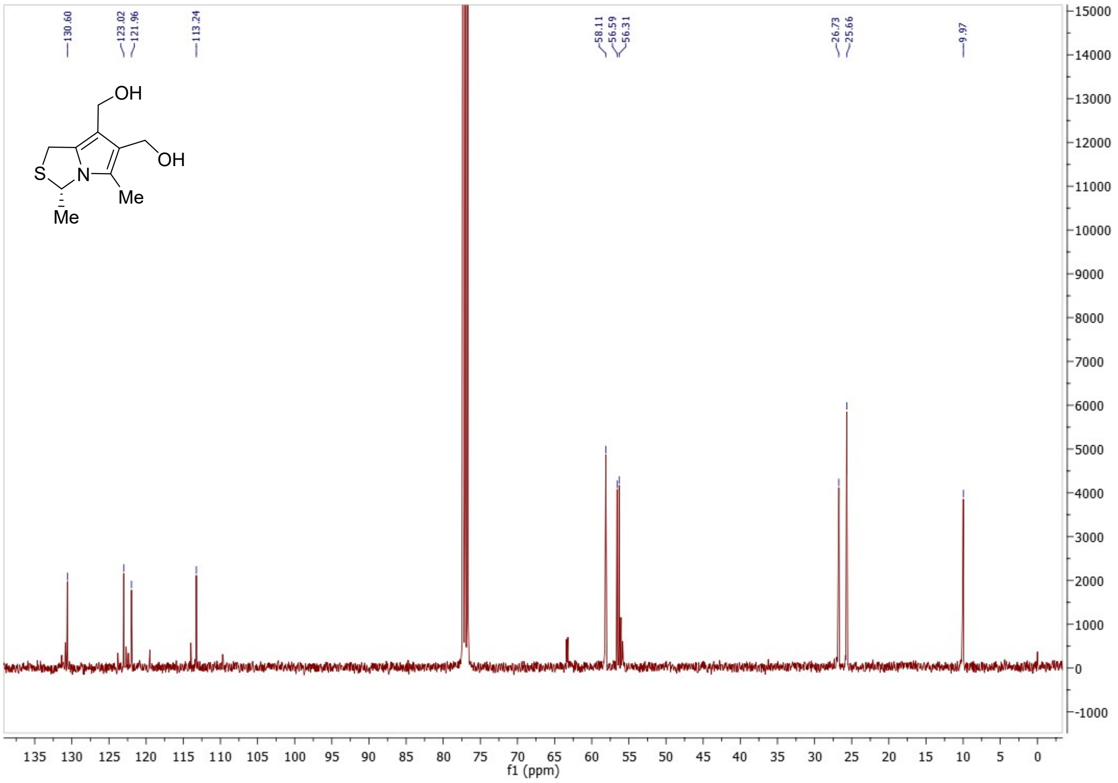

Figure S47: ^{13}C NMR spectrum of compound **17c** (100 MHz, $CDCl_3$).
